# A Bis‐Perylene Diimide Macrocycle Chiroptical Switch

**DOI:** 10.1002/anie.202501122

**Published:** 2025-02-10

**Authors:** Denis Hartmann, Samuel E. Penty, Martijn A. Zwijnenburg, Robert Pal, Timothy A. Barendt

**Affiliations:** ^1^ School of Chemistry University of Birmingham, Edgbaston Birmingham B15 2TT United Kingdom; ^2^ Department of Chemistry University of Durham South Road Durham DH1 3LE United Kingdom; ^3^ Department of Chemistry University College London 20 Gordon Street London WC1H 0AJ United Kingdom

**Keywords:** Macrocycle, Chiral Materials, Supramolecular Chemistry, Chiroptical Switch, Polycyclic Aromatic Hydrocarbons

## Abstract

Helical assemblies of organic dyes are ubiquitous chiral organic materials, with valuable properties including chiroptical switching due to the dynamic nature of supramolecular chirality. Herein, we report a novel chiral bis‐perylene diimide macrocycle, which acts as a discrete molecular model for a chiral supramolecular assembly. Point chirality is installed through amino acid‐derived imide groups which, upon macrocyclization, is translated into helical chirality in the perylene diimide dimer. In solution, the macrocycle's chiroptical properties are switchable, with both the sign (+/−) and amplitude (on/off) of the signal tuned using solvent and molecular recognition stimuli respectively. The chiral structure–property relationships identified from this macrocycle are important for the design of high fidelity supramolecular chiroptical switches.

## Graphical abstract

A chiral bis‐perylene diimide macrocycle has been prepared in which point chiral imide groups yield a discrete helical dimer, to mimic a helical supramolecular assembly. The macrocycle exhibits three‐way switching of its chiroptical properties in solution (+/−/off). This behaviour can be controlled by various stimuli, including the binding of polycyclic aromatic hydrocarbons, and identifies important structure‐property relationships for chiral materials.

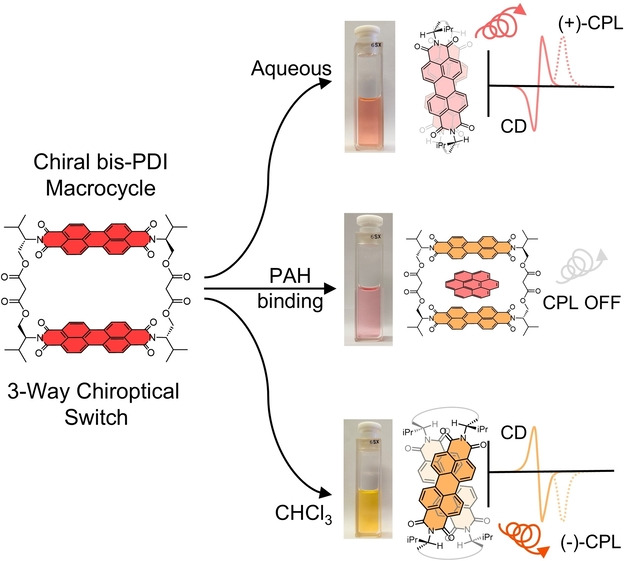

## Introduction

Chiral organic materials are under investigation for advancing the fields of chiral recognition,^[1]^ separation,^[2]^ and catalysis,^[3]^ while those containing organic chromophores^[4]^ unlock applications in chiroptical sensing,^[5]^ chiral optoelectronics,^[6]^ and spintronics.^[7]^ Among these materials, supramolecular assemblies are of great interest^[8]^ due to strong chiroptical properties arising from the amplification of chirality.^[9]^ These chiroptical properties include the absorption or emission of circularly polarised light,^[10]^ with respective circular dichroism (CD) or circularly polarised luminescence (CPL) quantified by the dissymmetry factors g_abs_ or g_lum_.

The adaptability of supramolecular systems to environmental factors enables the development of chiroptical switches,^[11]^ which have significant potential in molecular sensing, secure communications and information storage.[^12^, ^13^, ^14^] Chiroptical switches may turn the CD/CPL signal on or off.[^15^, ^16^] They may also invert the +/− sign of the CD or CPL signal, i.e. reverse the left/right handedness of the absorbed[^11^, ^17^] or emitted[^18^, ^19^, ^20^, ^21^, ^22^, ^23^, ^24^] circularly polarised light. However, to the best of our knowledge, there are no examples of a discrete organic molecule in which both the CPL signal (on/off) and sign (+/−) can be switched.

**Figure 1:**
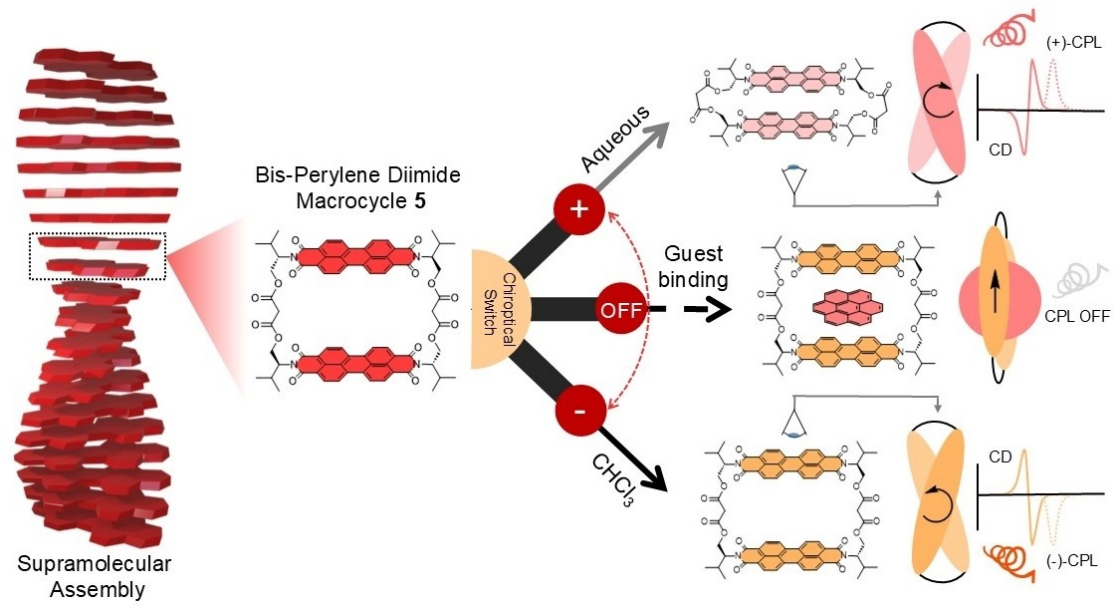
A chiral bis‐perylene diimide macrocycle (**5**) that mimics a helical supramolecular assembly and exhibits three‐way chiroptical switching, including circular dichroism (CD) and circularly polarised luminescence (CPL).

A common strategy to realising chiral supramolecular materials is to append point chiral side chain(s) to planar (achiral) organic dyes to direct their self‐assembly into helical superstructures (Figure 1).[^25^, ^26^, ^27^, ^28^] In a typical assembly, the dyes exhibit co‐facial π–π stacking with a rotational displacement (α).[^18^, ^20^, ^29^] This type of chiral supramolecular self‐assembly is essential for amplifying the CD or CPL signal through excitonic coupling between the dye monomers.^[10]^ Importantly, chiroptical properties will be diminished at smaller rotational displacements because, in the absence of a supramolecular helix, the chromophores are no longer in a chiral environment.^[30]^

Perylene diimides (PDIs) are organic dye molecules with outstanding photophysical, electrochemical and supramolecular properties.^[31]^ Although the parent PDI is achiral, the synthetic implementation of fixed point chirality from an auxiliary provides an efficient route to introducing supramolecular helical chirality.[^32^, ^33^, ^34^, ^35^, ^36^, ^37^, ^38^, ^39^, ^40^, ^41^] This auxiliary may be derived from the chiral pool and indeed many chiral PDIs have been reported with amino acid‐derivatives at the imide positions.[^2^, ^5^, ^42^, ^43^, ^44^, ^45^, ^46^, ^47^, ^48^, ^49^, ^50^, ^51^, ^52^, ^53^, ^54^, ^55^] However, a limited number of PDI systems report switchable chiroptical properties. These include chiral PDI assemblies which exhibit +/− CD switching[^55^, ^56^] and on/off CPL switching in a chiral PDI–binaphthol bisimide cyclophane.^[57]^ A fundamental understanding of the (supra)molecular factors that impact the sign and amplitude of the CD/CPL signal in switchable systems is critical for the design of functional chiroptical materials.

While most amino acid‐functionalized PDIs are used in extended supramolecular aggregates,[^42^, ^46^, ^48^, ^53^, ^55^, ^58^] we targeted a discrete intramolecular PDI dimer, a suitable model to establish chiral structure–property relationships upon conformational switching. Building on the success of using macrocyclic architectures to tune interactions involving PDIs for chiroptics,[^57^, ^59^, ^60^, ^61^, ^62^, ^63^] we designed a new imide‐connected bis‐PDI macrocycle, the first of its kind to use amino‐acid based linkers (Figure 1). We chose L‐valinol (macrocycle **5**) or D‐valinol (**7**) as amino acid derivatives for the imide residues because their sterically bulky isopropyl groups inhibit macrocycle aggregation, affording a discrete chiral PDI dimer. Macrocyclization by condensation with malonyl chloride affords a short, yet flexible, bis‐ester linkage,^[64]^ enabling the dimer's chiral conformation to be tuned via π–π interactions. This includes both intramolecular interactions between the PDI units and intermolecular π–π stacking through the binding of polycyclic aromatic hydrocarbon (PAH) guests in the macrocycle's cavity.

The resulting bis‐PDI macrocycle acts as an unprecedented three‐way chiroptical molecular switch, in which solvent stimuli are used to invert the sign of the CD/CPL signal, while PAH guest binding controls its amplitude (Figure 1). Furthermore, we use this preorganised, discrete, and switchable bis‐PDI dimer to explain how the changes in the CD and CPL are related to changes in the macrocycle conformation arising from tuneable π–π interactions. This knowledge underpins important design features concerning excitonic coupling, helicity, and rigidity, for switching the chiroptical properties of supramolecular materials.

## Results and Discussion

### Synthesis of Macrocycle and Characterisation

**Figure 2:**
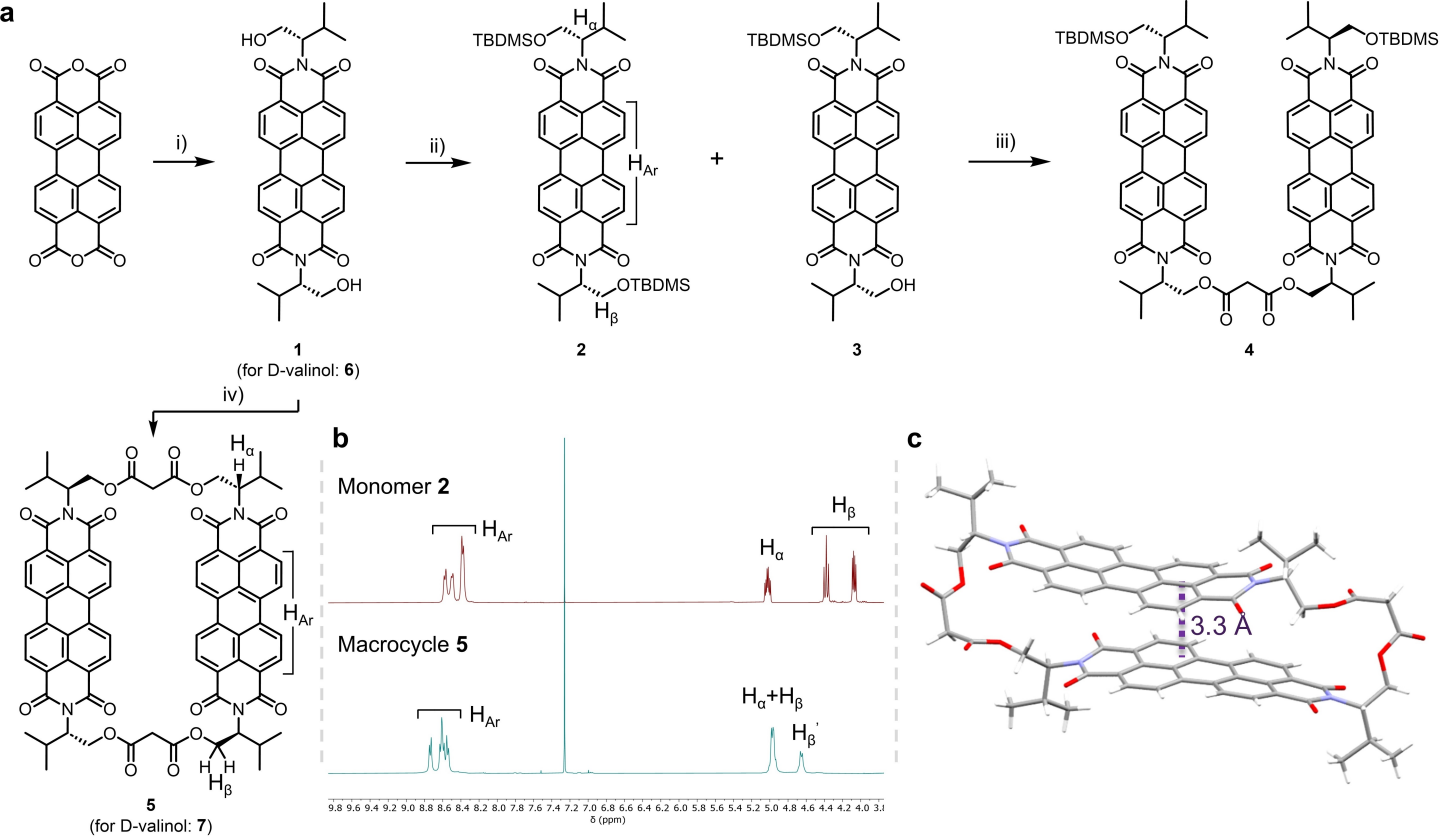
**a**) Synthesis of enantiomeric valinol‐bis‐PDI macrocycles **5** and **7**; i) L/D‐valinol, DMA, dioxane, 140 °C. 79 %. ii) TBDMSCl, imidazole, DMF. 12 % (**2**) and 36 % (**3**). iii) Malonyl dichloride, pyridine, DCM. 20 %. iv) Malonyl dichloride, pyridine, DCM. 6 %. **b**) Comparison of the ^1^H NMR spectra of L‐valinol‐monomer **2** and L‐valinol‐bis‐PDI macrocycle **5** in CDCl_3_, (298 K, 400 MHz). **c**) Single crystal structure of L‐valinol‐macrocycle **5**, with the π–π distance shown. Solvent molecules have been removed for clarity.

We set out to synthesise the envisioned chiral bis‐PDI macrocycles **5** and **7** that contained L‐ and D‐valinol respectively as the imide substituents. The synthesis of the L‐valinol‐derived macrocycle is shown in Figure 2a, and the procedure was the same for both enantiomers (see the Supporting Information). The chiral imide groups were installed through reaction of L/D‐valinol with perylene tetracarboxylic acid dianhydride in dimethylacetamide:1,4‐dioxane at 140 °C. These conditions were previously reported to allow for installation of imide groups with free alcohols,^[65]^ and in our case gave an excellent yield of the desired L‐ or D‐valinol‐PDI (**1** or **6**). From here, the target macrocycles **5** and **7** could be prepared directly via a [2+2] macrocyclisation reaction.^[64]^ Slow addition of malonyl chloride into a solution of **1**/**6** at high dilution in DCM with pyridine as the base enabled the synthesis of **5**/**7**, which was isolated through purification by silica gel column chromatography, followed by preparative thin layer chromatography.

We also synthesised an acyclic L‐valinol‐PDI‐dimer, **4**, to use as a control compound and enable comparisons with the more preorganised L‐valinol‐bis‐PDI macrocycle **5** (Figure 2a). To synthesise this, a statistical silyl protection using 1.1 eq. *tert*‐butyldimethylsilyl chloride in DMF with imidazole as the base was employed on **1**, which yielded the mono‐silylated PDI **3**, as well as small amounts of fully protected PDI **2**, which served as a useful monomeric control compound due its high solubility. Following on, the mono‐silylated PDI **3** was then reacted with malonyl chloride with pyridine as the base to yield the desired acyclic L‐valinol‐PDI‐dimer **4**.

In both the ^1^H NMR spectra of L‐valinol‐bis‐PDI macrocycle **5** (Figure 2b) and acyclic dimer **4**, the PDI aromatic protons (H_Ar_) show four sets of doublets due to the chirality of the imide groups, which gives the macrocycle and dimer *D*_2_ and *C*_2_ symmetry respectively. The chemical shift of the PDI proton signals of macrocycle **5** (and acyclic dimer **4**) are comparable to monomer **2** (Δδ=0.2 ppm, Figure 2b), suggesting that the PDI π‐surfaces are solvated and so reasonably far apart. Indeed, the magnitude of this shift is much smaller than in our previous bis‐PDI macrocycle design (Δδ up to 2 ppm),^[59]^ suggesting macrocycle **5** has a larger interchromophore separation in CDCl_3_ (i.e., >3.7 Å).

While chlorinated solvents are known to be ‘good’ solvents for solvating PDIs, their self‐association may be favoured in lower polarity solvents such as PhMe and cyclohexane,[^59^, ^66^] due to a stronger electrostatic contribution to the π–π interactions.^[67]^ However, when recorded in PhMe‐*d8*, the ^1^H NMR spectrum of macrocycle **5** is comparable to that in CDCl_3_, exhibiting sharp signals in both the aromatic region as well as for the macrocycle straps, in line with the spectrum of PDI monomer **2**. Therefore, solvation of the macrocycle's cavity by both PhMe and CHCl_3_ affords an ‘open’ conformation with a significant interchromophore separation. This outcome is consistent with the weak intramolecular excitonic coupling seen by UV/Vis spectroscopy and the PAH recognition properties of **5** in both these organic solvents (see below).

We were able to grow single crystals of macrocycle **5** from both CHCl_3_ and CH_2_Cl_2_ that were suitable for X‐ray diffraction (Figure 2c, Supplementary (Supp.) Figures 5‐1 and 5‐5). The crystal structures from both solvents showed minimal differences (Supp. Figure 5‐3) and revealed a ‘closed’ macrocycle conformation, due to π–π stacking between the two planar PDI surfaces. The PDI units exhibit a close π–π distance of 3.3 Å (Supp. Figure 5‐2). However, this distance is expected to be larger in PhMe and CHCl_3_ since the macrocycle's cavity is no longer empty (see above). In the solid state, the PDIs are displaced from a perfect co‐facial, co‐linear orientation along the long‐axis of the PDI units. The long‐axis displacement is 4.0 Å, while the short‐axis displacement is smaller (1.2 Å). These distances are consistent with an H‐type excitonically coupled PDI dimer.[^68^, ^69^]

Macrocycle **5** does not pack through intermolecular π‐stacking, likely due to the shielding of π‐surfaces by sterically demanding isopropyl‐groups of the L–Val‐derived imide residues. This intermolecular space is also filled with CHCl_3_ or CH_2_Cl_2_ solvent molecules (Supp. Figures 5‐1 and 5‐5), an arrangement that prevents aggregate‐caused quenching^[70]^ and ensures the crystals are visibly fluorescent (Supp. Figure 5‐4).

### Photophysical Properties

**Figure 3:**
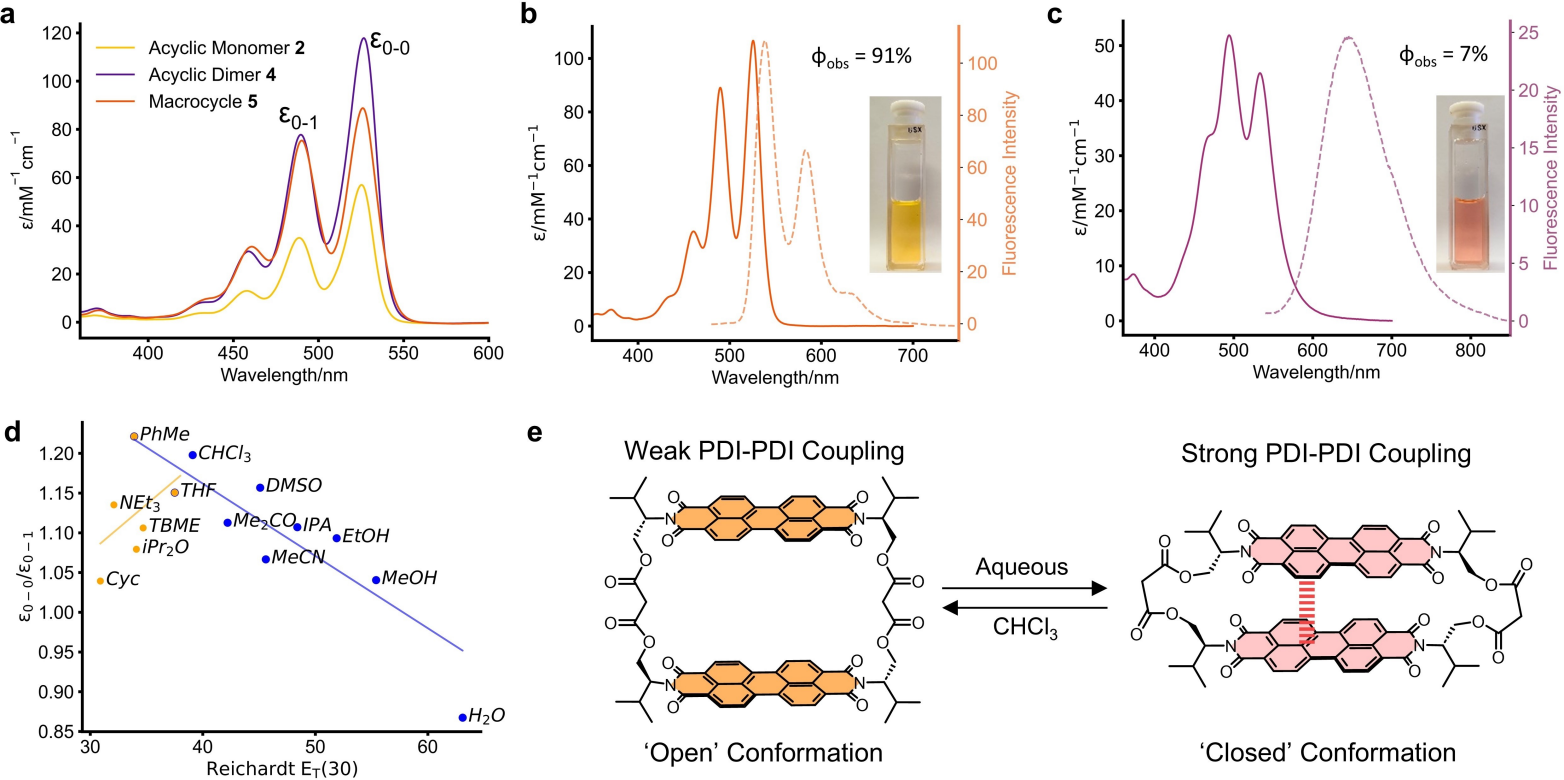
Photophysical properties of L‐valinol‐derived PDIs (298 K)**. a)** UV/Vis absorption spectra of **2**, **4** and **5** in CHCl_3_. **b**) Absorption and emission spectra of bis‐PDI macrocycle **5** in CHCl_3_ (λ_ex_=493 nm). **c**) Absorption and emission spectra of bis‐PDI macrocycle **5** in H_2_O with 0.1 % DMSO (λ_ex_=570 nm). **d**) A plot of ϵ_0‐0_/ϵ_0‐1_ of **5** in various solvents versus the Reichardt E_T_(30) scale of solvent polarity. **e**) Graphic of the solvent‐dependent conformation of bis‐PDI macrocycle **5**.

We next investigated the photophysical properties of PDI monomer **2**, acyclic dimer **4** and bis‐PDI macrocycle **5**, to identify evidence for PDI–PDI excitonic coupling and understand its dependence on dimer structure and conformation. The electronic absorption and emission spectra of **2**, **4** and **5** were initially measured in CHCl_3_ (Figure 3a, b). As seen in Figure 3a, the lowest energy PDI absorption band (S_0_→S_1_) is notable for a significant decrease in the ratio between the 0–0 and 0–1 vibronic peak intensities on going from monomer **2** (ϵ_0‐0_/ϵ_0‐1_=1.64), to acyclic dimer **4** (1.51), to bis‐PDI macrocycle **5** (1.18). The same trend is also seen in the vibrational progression of the fluorescence emission spectra (Supp. Figure 2‐1). This trend evidences an increase in H‐type excitonic coupling of the dyes,^[31]^ both in ground and excited states, upon their increasing preorganisation through covalent linkage via the PDI's L‐valinol imide groups.

The relative flexibility of the linkers in bis‐PDI macrocycle **5** motivated us to investigate the impact of solvent‐induced conformational change on intramolecular excitonic coupling. The absorption and fluorescence spectra of **5** in PhMe (Supp. Figures 2‐2 and 2‐3) are consistent with those in CHCl_3_ which, in line with ^1^H NMR spectroscopy, indicates a comparable interchromophore separation and so an ‘open’ dimer conformation in CHCl_3_/PhMe solvents (Figure 3e). This outcome contrasts with the CHCl_3_/PhMe conformational change of our previous bay‐connected bis‐PDI macrocycle,^[59]^ for which the smaller cavity is not solvated by PhMe and so intramolecular interactions are enhanced in this solvent.^[71]^

Macrocycle **5** can be solubilised in H_2_O with 0.1 % DMSO/THF, with no evidence of precipitation after at least ten days (Supp. Figures 2‐3 and 2‐4). This allowed us to examine the effect of aqueous solvent since π–π interactions are often stronger in polar, protic solvents.^[67]^ On changing the solvent from CHCl_3_ to aqueous there is a colour change of **5** that is visible to the naked eye (Figure 3b,c). As such, we observed a drastic change in the absorption spectrum under aqueous conditions at 5 μM (Figure 3c), most notably an inversion of the 0–0 and 0–1 vibronic peak intensities (ϵ_0‐0_/ϵ_0‐1_=0.87), leading to a blue shift of the S_0_→S_1_ absorption band maximum (Δλ=−33 nm).

These spectral changes are evidence for a large increase in H‐type excitonic coupling in H_2_O.^[31]^ Importantly, we confirmed that this is an intramolecular effect through Beer–Lambert plots in H_2_O‐DMSO/THF (Supp. Figures 2‐6 to 2‐9), where deviation from linearity only occurred at much higher concentrations of **5** (>150 μM). Therefore, the increase in excitonic coupling in H_2_O arises from a smaller distance between the two PDI chromophores, meaning that the macrocycle collapses in on itself (Figure 3e), now adopting a ‘closed’ conformer, most likely with a d_π–π_ closer to that in the crystal structure (Figure 2c). This conformational change was also confirmed by ^1^H NMR spectroscopy, whereby the addition of D_2_O to a sample of **5** in DMSO‐*d6* leads to a shifting and broadening of PDI aromatic signals as well as stronger through‐space interactions in the NOESY NMR spectrum (Supp. Figures 7‐1 to 7‐3).

Intramolecular PDI coupling is also stronger in the excited state of the bis‐PDI macrocycle **5** in H_2_O, due to the formation of a PDI excimer. Compared to the relatively monomeric emission profile of **5** in CHCl_3_ (Figure 3b), the fluorescence spectrum in H_2_O (Figure 3c) contains a broad, featureless emission band with a larger Stokes‐shift (Δλ=130 vs. 13 nm) and quenched emission (ϕ_obs_=7 % vs. 91 %, Supp.Table 2‐1).^[72]^ In addition, the average lifetime was reduced and showed more complex behaviour in H_2_O compared to CHCl_3_ (Supp. Table 2‐2, Supp. Figure 2‐10). Therefore, **5** demonstrates that red‐shifted fluorescence emission in bis‐PDI macrocycles may occur from an H‐type coupled dimer due to excimer formation.^[31]^ From an organic solvent into aqueous solvent (DMSO‐H_2_O) UV/Vis spectroscopic titration, isosbestic points indicate that the conformation‐photophysical switching behaviour of **5** is reversible, with the ‘open’ and ‘closed’ conformations in equilibrium (Figure 3e, Supp. Figure 2‐11). Further analysis allowed us to estimate the free energy of intramolecular aggregation of bis‐PDI macrocycle **5** to be Δ*G*_agg_=−9 kJ mol^−1^ in H_2_O (Supp. Figures 2‐12 and 2‐13).^[73]^

To further investigate the solvent dependence of this intramolecular aggregation, we measured the UV/Vis spectrum of **5** in a wide range of solvents (Supp Figures 2‐14 and 2‐15). Overall, we found that the excitonic coupling, which is proportional to the degree of intramolecular π–π aggregation, follows a solvent trend seen for intermolecular PDI aggregates (Figure 3d).^[66]^ As such, intramolecular π–π aggregation in **5** is favoured in low polarity, aprotic solvents (e.g., cyclohexane) or polar, protic solvents (e.g., H_2_O) due to the respective importance of electrostatic or dispersion/solvophobic forces to the π–π interactions.^[67]^ Indeed, dissolving **5** in cyclohexane (with 0.1 % THF or 2 % CHCl_3_) also causes population of the ‘closed’ macrocycle conformation (Supp. Figures 2‐16 and 2‐17). However, the effect of H_2_O is more pronounced, which, in line with previous results on intermolecular PDI aggregates, is indicative of a hydrophobic effect in aqueous media.^[66]^

### Chiroptical Properties

**Figure 4:**
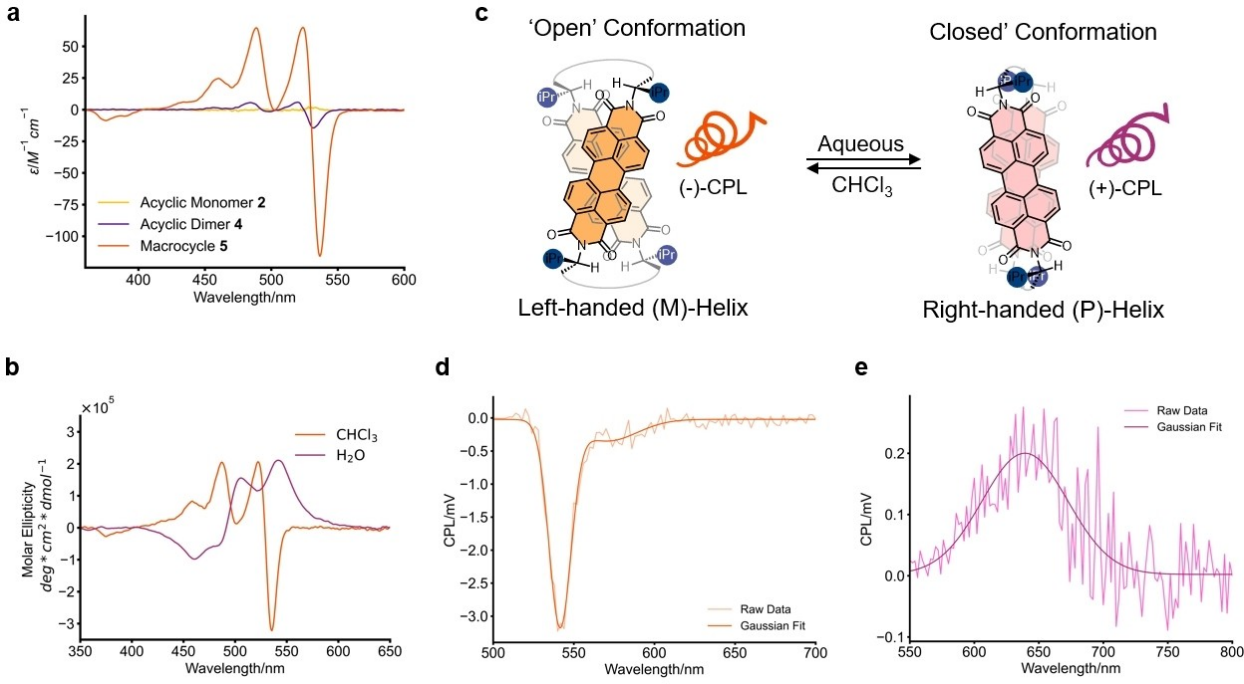
Chiroptical properties of L‐valinol‐derived PDIs (298 K). **a**) CD spectra of **2**, **4** and **5** in CHCl_3_ (**2** and **4** at 15 μM, 1 mm pathlength; **5** at 15 μM, 1 cm pathlength). **b**) CD spectra of bis‐PDI macrocycle **5** in CHCl_3_ and H_2_O (both with 0.1 % DMSO at 5 μM). **c**) Graphic of the solvent‐dependent helicity of bis‐PDI macrocycle **5**. **d**) CPL spectrum of bis‐PDI macrocycle **5** in CHCl_3_ (5 μM, λ_ex_=450 nm). **e**) CPL spectrum of bis‐PDI macrocycle **5** in H_2_O with 0.4 % DMSO (20 μM, λ_ex_=491 nm).

The introduction of point‐chiral, amino‐acid‐derived imide groups was expected to impart supramolecular helical chirality onto the enantiomeric bis‐PDI macrocycles **5** and **7**. This warranted investigation by circular dichroism (CD) and circularly polarised luminescence (CPL) spectroscopy, to understand the impact of PDI dimer preorganisation and conformational change on chiroptical properties. While the monomeric PDI **2** exhibited very weak CD in CHCl_3_ (g_abs_=4×10^−5^), the magnitude of this dissymmetry factor increased substantially upon increasing excitonic coupling^[10]^ in acyclic dimer **4** (−2×10^−4^) and ultimately L‐valinol‐bis‐PDI macrocycle **5** (−2×10^−3^), as seen in Figure 4a. This trend was consistent in other organic solvents, including PhMe (Supp. Figure 2‐18), yet previous amino acid‐functionalised PDIs have required aggregation in polar, protic solvents to elicit a CD spectrum.[^42^, ^55^] Therefore, macrocycle **5** demonstrates the value of preorganisation to amplifying g_abs_ in any solvent, for systems that translate point chirality into supramolecular helical chirality. This includes the differential absorption of circularly polarised light across a wider spectral window, courtesy of a new CD signal for the S_0_→S_2_ transition of **5** (g_abs_=−2×10^M‐>3^ at 375 nm), which is absent in the control compounds **2** and **4** (Figure 3a).

Importantly, both acyclic dimer **4** and macrocycle **5** exhibit a (−)‐Cotton effect at λ_max_ of the S_0_→S_1_ absorption band (λ=525 and 530 nm, respectively), which is absent for monomer **2** (Figure 3a). This bisignate profile arises because the co‐facial H‐type PDI dimer exhibits a rotational displacement of the two chromophores, with the sign of the Cotton effect indicating a left‐handed, or M‐helix (Figure 3c).^[30]^ We confirmed this helical chirality assignment using (time‐dependent) density functional theory ([TD]‐DFT) calculations. We optimised the structure of L‐valinol‐bis‐PDI macrocycle **5** obtained from the crystal structure using DFT (B97‐3c),[^74^, ^75^] as well as an alternative low‐energy conformation for **5** obtained from a conformer search (using the CREST code^[76]^ and the GFN2‐xTB tight‐binding method^[77]^), and subsequently predicted their UV/Vis and CD spectra using TD‐DFT (TD‐ωB97x) in CHCl_3_.[^74^, ^75^] These calculations show that; (i) the structure obtained from the crystal structure becomes more helical upon optimisation (Supp. Figure 6‐1), (ii) both structures are predicted to be an M‐helical intramolecular PDI dimer (Supp. Figures 6‐1 and 6‐2), and (iii) both structures are essentially isoenergetic. Furthermore, the predicted UV/Vis and CD spectra show H‐type excitonic coupling and a (−)‐Cotton effect (Supp.Tables 6‐1 and 6‐2, Supp. Figures 6‐3 and 6‐4), both consistent with experiment. We note that the structures of **5** predicted by DFT have a smaller interchromophore separation than that expected in organic solvent from experiments but this difference will not impact the sign of the Cotton effect.^[78]^ This smaller separation is most likely because of the use of an implicit solvent model for CHCl_3_ in the calculations such that, as in the crystal structure, the macrocycle's cavity is not explicitly solvated.

The CD spectrum of **5** is weakened upon increasing the temperature (Supp. Figure 2‐19), since increased molecular motion likely leads to the unwinding of helicity. We exploited the dynamic nature of the macrocycle's supramolecular helicity by using a solvent stimulus to trigger CD switching. While the (−)‐Cotton effect was consistent for **5** in other solvents (Supp. Figure 2‐20), in H_2_O (with 0.1 % DMSO or THF), the sign of the Cotton effect is reversed and blue‐shifted (to the 0–1 transition λ_max_), the overall effect being a near inversion of the CD spectrum in CHCl_3_ (Figure 4b, Supp. Figure 2‐21). A similar, yet less pronounced, effect is also seen in cyclohexane with 0.1 % THF (Supp. Figure 2‐22). Since the PDI dimer continues to exhibit H‐type coupling in H_2_O, this new (+)‐Cotton effect means the macrocycle **5**’s supramolecular chirality is inverted from an M‐helix to a P‐helix on going from CHCl_3_ to aqueous solvent (Figure 4c).^[30]^ We confirmed this chirality assignment by TD‐DFT which, in line with experiment, showed a (+)‐Cotton effect in the predicted CD spectrum of an optimised P‐helical macrocycle structure of **5** in H_2_O.

Mirror image chiroptical switching behaviour is observed experimentally with the D‐valinol‐derived macrocycle **7** (Supp. Figures 2‐23 and 2‐24) which, relative to its enantiomer, L‐valinol macrocycle **5**, exhibits mirror‐image CD spectra in CHCl_3_ ((+)‐Cotton effect) and H_2_O ((−)‐Cotton effect). The tuning of supramolecular helicity using achiral solvents has been previously reported,[^17^, ^19^, ^21^, ^24^, ^32^, ^36^, ^79^, ^80^, ^81^, ^82^] but it is realised for the first time here in a discrete intramolecular PDI dimer, courtesy of the macrocyclic architectures of **5** and **7**.

The macrocycle's helical switching is intrinsically connected to the ‘open’ ⇆ ‘closed’ conformational change (Figures 3d and 4c). This is because an analogous solvent into solvent (DMSO‐H_2_O) CD titration showed a reversible sign inversion (Supp. Figure 2‐25 and 2‐26), with a turning point at the same point as seen by UV/Vis spectroscopy (~33 % H_2_O). At this point the CD signal is weakened due to the population of both right‐ and left‐handed helical conformers, although a CD signal is still observed since the CD spectra are not mirror images (Figure 4b). Furthermore, the weaker CD signal in cyclohexane also correlates with the weaker intramolecular π–π aggregation in this solvent compared to H_2_O (Figure 3d). We propose that the inversion of supramolecular helicity is most pronounced in H_2_O because of the hydrophobic collapse of the amino acid‐derived PDI units, analogous to that observed in protein assemblies.^[83]^ This conformational change minimises the hydrophobic surface area by shielding regions such as the PDI π‐surfaces and *i*Pr groups. Notably, for the L‐valinol *i*Pr groups to be in close proximity in **5**, the PDI dimer must switch from a left‐handed to a right‐handed helix (Figure 4c).

While the sign of the macrocycle's dissymmetry factor changes in aqueous solvent, the magnitude is the same as that in CHCl_3_ (Table 1), despite the stronger excitonic coupling in H_2_O. This suggests that, as well as a shorter PDI–PDI separation, the dimer also has a smaller rotational displacement in H_2_O, potentially like that in the crystal structure of **5** which also exhibits intramolecular π–π interactions (Figure 2c). In solution, however, this ‘closed’ conformation will be more dynamic and indeed the Cotton effect observed in H_2_O is evidence for the rotational displacement of co‐facially stacked PDIs (Figure 4b).

**Table 1:** Dissymmetry factors (g_abs_ and g_lum_) for macrocycle **5** and its complex with coronene. Errors in g_abs_ and g_lum_ are ±2×10^−4^ and ±5×10^−5^ respectively.

Index	Species	Solvent	g_abs_ (×10^−3^)/[λ/nm]	g_lum_ (×10^−3^)/[λ/nm]
**1**	**5**	CHCl_3_	−2 [539]	−2 [542]
**2**	**5**	H_2_O	+2 [551]	+1 [640]
**3**	**5‐coronene** (95 % complexation)	CHCl_3_	−0.4 [550]	0

Importantly, bis‐PDI macrocycle **5** also acts as a CPL switch, since the handedness of the CPL signal is inverted on going between CHCl_3_ and H_2_O (Figure 4d, e). Macrocycle **5** exhibits a negative band in the CPL spectrum in CHCl_3_ and a positive band in H_2_O. These CPL signals match the sign of the lowest energy branch of their CD spectra, indicating that, in the excited state, the M‐ or P‐helicity of **5** is maintained in the chlorinated or aqueous solvent, respectively (Table 1, Supp. Table 3‐1, Supp. Figures 3‐1 to 3‐3).

The macrocycle is most strongly fluorescent in CHCl_3_ (ϕ_obs_=91 %) which contributes to a relatively high CPL brightness of B_CPL_=68 M^−1^ cm^−1^ for organic emitters.^[84]^ Interestingly, the 0–1 and 0–2 vibrational progression peaks seen in the fluorescence spectrum of **5** in CHCl_3_ have drastically reduced intensities in the CPL spectrum (Figure 4d). This suggests that the population of vibrationally excited states reduces supramolecular helicity, akin to the reduction of the CD signal upon heating.

In comparison to CHCl_3_, the macrocycle's g_lum_ is lower than g_abs_ in H_2_O (Table 1), indicating a larger change in chiral conformation between ground and excited states occurs under aqueous conditions. This observation is consistent with the bis‐PDI macrocycle forming an excimer in H_2_O (Figure 3c). Indeed, previous calculations have shown that a PDI excimer favours an almost co‐linear orientation in a co‐facial arrangement,^[85]^ an excited‐state geometry that will reduce the helical twist of the macrocyclic PDI dimer and so lower its g_lum_ in solution.

### Host–Guest Chemistry

**Figure 5:**
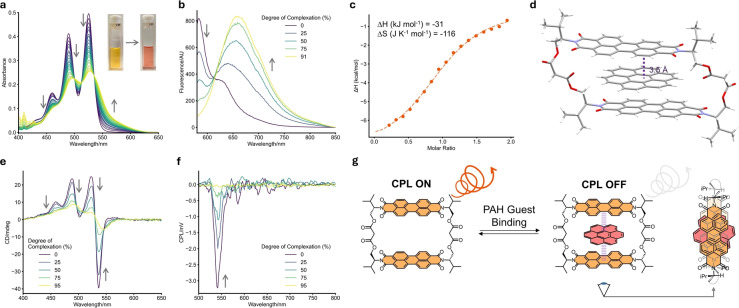
The host–guest chemistry of macrocycle **5**. **a**) UV/Vis spectroscopic titration between host **5** (5 μM) and up to 52 equivalents of coronene guest in CHCl_3_ (298 K). The photos show the change in colour of the solution upon coronene complexation. **b**) Fluorescence spectrum of **5** (5 μM) upon addition of coronene at different degrees of complexation (in %), excited at λ_ex_=570 nm, the charge‐transfer band, to excite the complex. Exciplex emission is observed. **c**) ITC thermogram for coronene binding by **5** in CHCl_3_. **d**) Single crystal structure of **5**–coronene co‐crystals, showing the 1 : 1 binding stoichiometry of the host–guest complex. **e**) Changes in CD spectra of **5** at different degrees of complexation (in %) with coronene as guest (100 μM for **5**). **f**) Changes in CPL spectra of **5** at different degrees of complexation (in %) with coronene as guest. (5 μM for **5**, λ_ex_=490 nm). **g**) Graphic of coronene guest binding by **5**.

The potential for the bis‐PDI macrocycle to adopt an ‘open’ conformation and its propensity for π–π stacking interactions, as seen in the ‘closed’ conformation (Figure 3e), prompted us to investigate the molecular recognition properties of **5** with PAH guests (Figure 5d). These studies were conducted in CHCl_3_ and PhMe to minimise the competition from intramolecular π–π stacking between the PDI units. UV/Vis spectroscopic titrations revealed the binding of several PAH guests by evolution of the PDI S_0_→S_1_ absorption band, leading to the emergence of isosbestic points and new red shifted bands characteristic of host–guest electronic interactions, including charge‐transfer between the PDI π‐acceptor and PAH π‐donor (Figure 5a, Supp. Figures 4‐1 to 4‐10).^[86]^ Association constants (*K*_a_) were obtained by non‐linear curve fitting of the titration data to a 1 : 1 host–guest stoichiometric model (Table 2),[^87^, ^88^, ^89^] as validated by single crystal X‐ray diffraction (Figure 5c). For planar PAHs, binding gets stronger with host preorganisation (**4**<**5**) and upon increasing guest π‐surface area (pyrene < perylene < coronene), the latter trend being in line with previous PAH hosts.[^90^, ^91^] Macrocycle **5** also bound the non‐planar guest corannulene, which has rarely been studied,[^91^, ^92^, ^93^, ^94^] and only been explored computationally with PDI‐based hosts that do not contain bay substituents.^[95]^

**Table 2:** Association constants (*K*_a_) for PAH guests.

Index	Host	Guest	*K*_a_/M^−1^ in PhMe	*K*_a_/M^−1^ in CHCl_3_
1	**4**	Pyrene	48	No binding
2	**5**	Pyrene	110	54
3	**5**	Corannulene	190	190
4	**5**	Perylene	540	1,100
5	**5**	Coronene	29,000	38,000

^a^1 : 1 host–guest stoichiometry, *K*_a_ determined by UV/Vis spectroscopy titrations at 298 K with all fitting errors <5%.

The binding of the best guest we tested, coronene, was also examined by isothermal titration calorimetry (Figure 5c, Supp. Figures 4‐11 and 4‐12, Supp. Table 4‐1) in CHCl_3_ and ^1^H NMR spectroscopy in CDCl_3_ (Supp. Figure 7‐4). The former gave a *K*_a_ in good agreement with that obtained by UV/Vis spectroscopy (i.e., of the order of 10^4^ M^−1^) and revealed the binding is enthalpically driven, Δ*H*=−31.0 kJ mol^−1^, while Δ*S*=−116 J K^−1^ mol^−1^. By ^1^H NMR spectroscopy, the addition of one equivalent of coronene to **5** led to a broadening and upfield shift of the PDI aromatic signals, while the malonyl‐CH_2_ signal moves downfield, as expected for a co‐facial π‐stacked orientation in the macrocycle's cavity. Indeed, this binding mode and stoichiometry was confirmed by three X‐ray diffraction structures of co‐crystals of macrocycle **5** with bound PAH guests, pyrene, perylene and coronene (Figure 5d, Supp. Figures 5‐6 to 5‐8). The encapsulated guest forms close π–π contacts of 3.5 Å with the PDI units and the crystal structure packing reveals that a PAH is also sandwiched between the exterior π‐surfaces of neighbouring macrocycles (3.6 Å). While the bulky *i*Pr groups prevented such packing between PDI units in the structure of **5** alone, smaller PAHs can now fill this gap.

We also investigated the effect of guest binding on the fluorescence emission of the macrocycle for the two strongest bound guests, perylene and coronene, in CHCl_3_ (Figure 5b, Supp. Figures 4‐13 to 4‐17). For both guests, binding causes a decrease in intensity of the PDI emission of **5** and, upon excitation of the host–guest charge transfer band (Figure 5b and Supp. Figure 4‐17, λ=570 nm), the growth of a new red‐shifted and broadened emission peak, characteristic of the formation of an exciplex^[96]^ with charge‐transfer character.^[97]^ The Stokes shift is largest for the **5**–coronene exciplex (Δλ=116 nm), which shows that it is lower in energy than the **5**–perylene exciplex (Δλ=88 nm).

Finally, we investigated the impact of PAH recognition on the bis‐PDI macrocycle's chiroptical properties by performing CD and CPL spectroscopic titrations with perylene and coronene in CHCl_3_. For both guests, the CD of **5** became weaker upon binding (Table 1, Figure 5e, Supp. Figures 4‐18 to 4‐25) and no new CD signal was induced in the bound PAH, even when the degree of complexation reached 95 % (for coronene). Upon extrapolating this trend to complete complexation (Supp. Figures 4‐20 and 4‐25), we find that the loss of CD is more substantial in the **5**–coronene complex (a 96 % decrease relative to the free host) than the **5**–perylene complex (66 %). While PAH binding does not change the sign of the Cotton effect and thus the macrocycle's helicity, it does red‐shift the CD spectrum, which is consistent with the UV/Vis spectroscopy studies. Importantly, as for +/− chiroptical switching (Figure 4b), the on/off chiroptical switching is also reversible since the strength of the macrocycle's CD signal increases upon decreasing the degree of coronene complexation (Supp. Figure 4‐26 to 4‐30).

A similar, yet more drastic, effect was observed from the CPL titrations, with the macrocycle's CPL signal fully depleted when the degree of complexation reached >90 % with either guest (Figure 5f, Supp. Figures 4‐31 to 4‐34). Interestingly, no CPL was observed from the exciplex emission band either, i.e., when the complex was excited at λ=570 nm. Therefore, bis‐PDI macrocycle **5** acts as a three‐way chiroptical switch, covering both CD/CPL sign and signal strength, and operable through the (achiral) stimuli of solvent medium and PAH guest binding (Figure 1). To the best of our knowledge, this is the first example of CPL +/−/off switching of a discrete molecule in solution.

The attenuation of chiroptical properties upon achiral guest binding may be explained by a reduction in supramolecular helicity of the flexible host to optimise intermolecular π–π stacking in the **5**–PAH complex, a conformational change supported by the co‐linearity of the PDI dimer in the co‐crystals (Figure 5d and Supp. Figure 5‐9). For CD, the larger π‐surface area of coronene appears to have a greater impact than perylene. Importantly, the depletion of the CPL cannot be explained entirely by fluorescence quenching, since the macrocycle–PAH exciplexes are emissive, e.g., ϕ_obs_=7 % for **5**–coronene (degree of complexation=95 %). Instead, we propose that π–π interactions between the flexible host **5** and PAH guest cause a complete unwinding of the macrocycle's helical conformation to form a co‐linear ‘achiral’ exciplex (Figure 5g). This effect is comparable to the intramolecular excimer in H_2_O which also lowered g_lum_ and complements previous reports of rigid exciplexes which are CPL active.[^18^, ^19^, ^98^, ^99^]

### Summary and Conclusions

In summary, we have prepared a novel bis‐PDI macrocycle using L/D‐valinol as the imide groups (**5**/**7**), which successfully translates point chirality from L/D‐valinol to supramolecular helical chirality in the resulting intramolecular PDI dimer (Figure 1). Courtesy of the macrocyclic architecture, the chiral PDI dimer is a discrete, persistent, and dynamic system for understanding structure‐chiroptical property relationships in extended chiral supramolecular assemblies of dyes.[^18^, ^42^, ^46^, ^58^, ^100^] The sign of the CD/CPL signal can be inverted using a solvent stimulus, since macrocycle **5** exhibits opposite supramolecular helicity in CHCl_3_ (M‐helix) and aqueous (P‐helix) media, as governed by intramolecular π–π interactions. The macrocycle is also notable for its molecular recognition of PAHs (*K*_a_ >10^4^ M^−1^), which controls the amplitude of the CD/CPL signal. Therefore, bis‐PDI macrocycle **5** (and its enantiomer, **7**) represents a unique three‐way chiroptical switch (+/−/off) of potential value for chiroptical imaging, sensing and spintronics.

Solution, solid state, and computational studies reveal how the macrocyclic dimer's switchable chiral conformation impacts the sign and magnitude of the absorption and emission of circularly polarised light. This includes tuning the interchromophore separation as well as the size and direction of their rotational displacement. We propose that while dimer preorganisation enhances H‐type excitonic coupling and, in turn, the chiroptical signal, there is a potential trade‐off if the increase in electronic coupling in the dimer comes at the cost of reducing supramolecular helicity.^[30]^ Indeed, despite stronger excitonic coupling in H_2_O, the dissymmetry factors of **5** are either unchanged (g_abs_) or lower (g_lum_) in aqueous media compared to CHCl_3_, due to a more co‐linear arrangement of the intramolecular PDI dimer/excimer. Ultimately, coronene binding causes a loss of the CD/CPL signal because the formation of the complex/exciplex leads to an unwinding of macrocycle helicity. Therefore, our bis‐PDI macrocycle demonstrates the advantage of dynamic supramolecular chirality for realising chiroptical switching in helical assemblies and how the relative arrangement of the chromophores is key to controlling this phenomenon.

## Supporting Information

Synthetic procedures for compounds **1–7**, characterisation data, additional spectra, titration data, single crystal diffraction data, DFT optimised structures, and experimental and computational methods can be found in the Supporting Information.

Deposition Number(s) 2379172 (for ValPDIMC_CHCl3), 2379183 (ValPDIMC_CH2Cl2), 2379184 (for ValPDIMC_Pyrene), 2379198 (for ValPDIMC_Perylene), 2379186 (for ValPDIMC_Coronene) contain the supplementary crystallographic data for this paper. These data are provided free of charge by the joint Cambridge Crystallographic Data Centre and Fachinformationszentrum Karlsruhe Access Structures service.

## Conflict of Interests

The authors declare no conflict of interest.

1

## Supporting information

As a service to our authors and readers, this journal provides supporting information supplied by the authors. Such materials are peer reviewed and may be re‐organized for online delivery, but are not copy‐edited or typeset. Technical support issues arising from supporting information (other than missing files) should be addressed to the authors.

Supporting Information

Supporting Information

Supporting Information

Supporting Information

Supporting Information

## Data Availability

The data that support the findings of this study are available in the supplementary material of this article.
