# Supplementary material for: A Bis‐Perylene Diimide Macrocycle Chiroptical Switch
Source: Angew Chem Int Ed Engl. 2025 Feb 10;64(15):e202501122. doi: 10.1002/anie.202501122 (PMC11976202; doi:10.1002/anie.202501122)
Supplement: Supplementary file 5 — Supporting Information [file ANIE-64-e202501122-s002.pdf]

Angewandte Chemie
Eine Zeitschrift der Gesellschaft Deutscher Chemiker

Supporting Information

A Bis-Perylene Diimide Macrocycle Chiroptical Switch

*D. Hartmann, S. E. Penty, M. A. Zwijnenburg, R. Pal, T. A. Barendt**

Supplementary Information

A bis-perylene diimide macrocycle chiroptical switch

Authors: Denis Hartmann[1], Samuel E. Penty[2], Martijn Zwijnenburg[3], Robert Pal[2], Timothy A. Barendt[1,*]

Affiliations:

[1]School of Chemistry, University of Birmingham, Edgbaston, Birmingham B15 2TT, United Kingdom

[2]Department of Chemistry, University of Durham, South Road, Durham DH1 3LE, United Kingdom

[3]Department of Chemistry, University College London, 20 Gordon Street, London WC1H 0AJ, United Kingdom

Contents

1) Synthesis 2
2) Photophysical Properties 11
3) CPL Measurements 30
4) Host/Guest Chemistry 34
5) X-Ray Crystallography 55
6) Density Functional Theory 63
7) NMR Spectra 68
8) Mass Spectra 84
9) IR Spectrum 91
10) References 92

1) Synthesis

General Synthesis

Reagents were purchased from commercial sources (Merck, Acros Organics, Fluorochem and Alfa Aesar) and used without further purification. Solvents were used as supplied (analytical/HPLC-grade from Fisher or Sigma-Aldrich). If required, solvents were dried over molecular sieves (3 Å) overnight before use and stored under N_2 atmosphere. Petroleum ether (PE) over a boiling point range of 40–60 °C was used. Eluent mixtures are reported in volume:volume or %vol. Column chromatography was carried out using Merck Silica Gel 60 Å, 230-400 mesh, 40-63 µm particle size. TLC was carried out on Merck silica gel 60 F254 Al plates. Preparative TLC was performed using 20 × 20 cm plates with 1 cm silica thickness. NMR spectroscopy measurements were recorded using a Bruker AVIII300, AVIII400, AV Neo 400 or AV Neo 500 instrument and peaks were referenced to the residual solvent peak. Electrospray Ionisation Mass Spectrometry (ESI-MS) measurements were carried out Waters Xevo G2-XS TOF Mass Spectrometer**.** Infrared Spectra (IR) were recorded on a Perkin-Elmer Spectrum Two FT-IR Spectrometer and classified as strong (s), medium (m), weak (w) and broad (b).

L-Valinol-Perylene Diimide (PDI) 1

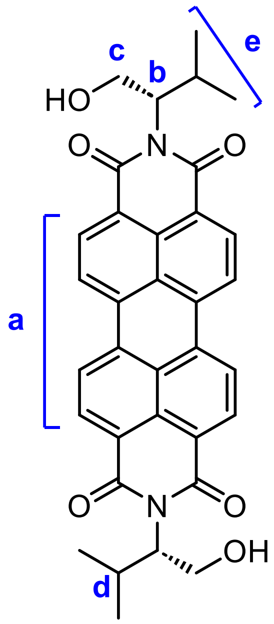

Procedure adapted from the literature:[1] To a round-bottom flask under N_2 atmosphere was added perylene tetracarboxylic acid dianhydride (1 g, 2.55 mmol), L-valinol (539 mg, 5.23 mmol), 1,4-dioxane (10 mL) and dimethylacetamide (10 mL). The reaction mixture was stirred at 140 °C for 24 h. The mixture was then cooled, and the solvent reduced *in vacuo*. The resulting residue was purified by flash column chromatography (SiO_2, 4% MeOH/$CHCl_3$) to yield a dark red/brown solid as the title compound (1.13 g, 2.01 mmol, 79%).

R_f (SiO_2, 4% MeOH/$CHCl_3$) 0.21.

^{1}H-NMR (400 MHz, $CDCl_3$) δ/ppm 8.66 – 7.66 (m, 8H, CH_{Ar} **a**), 5.08 (td, J = 10.1, 4.0 Hz, 2H, imide C-H, **b**), 4.52 (s, 2H, diastereotopic CH_2, **c'**), 4.17 (d, J = 11.5 Hz, 2H, diastereotopic CH_2, **c''**), 2.64 (s, 2H, iPr C-H, **d**), 1.17 (d, J = 6.5 Hz, 6H, CH_3 iPr, **e'**) & 0.81 (broad s, 6H, iPr CH_3, **e''**).

Due to poor solubility, no ^{13}C NMR could be obtained.

HRMS (ESI$^+$) found 563.2162 $[M+H]^+$, $[H_{35}C_{30}N_2O_6]^+$ requires 563.2176.

UV/Vis ($CHCl_3$) λ/nm (ε/mM^{-1} cm^{-1}) 529 (57), 492 (35), 461 (13)

TBDMS protection of L-Valinol-Perylene Diimide

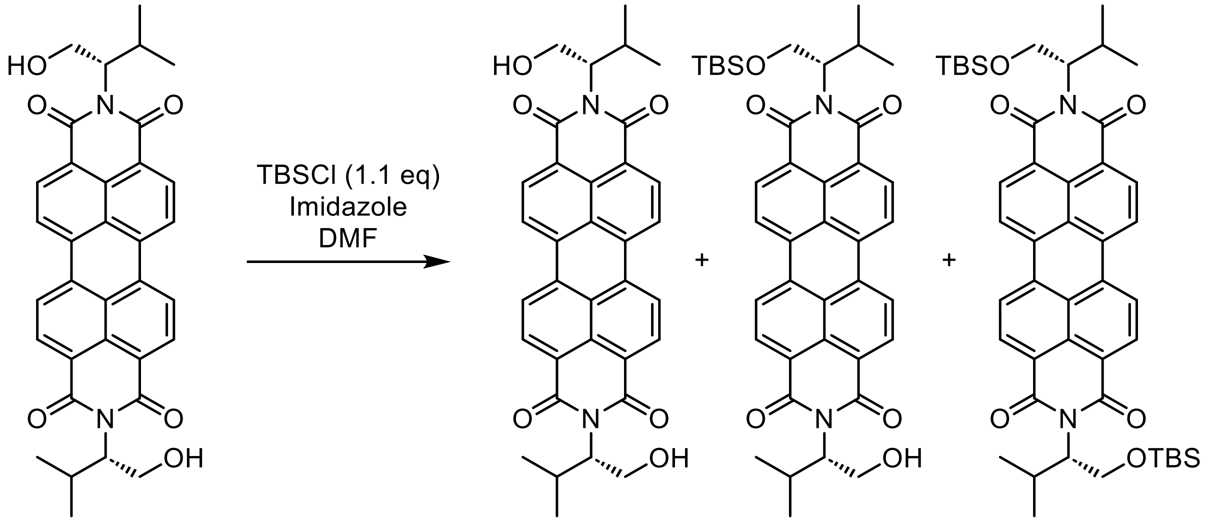

In a dry round bottom flask under N_2 atmosphere, L-Valinol-PDI **1** (1.2 g, 2.13 mmol), *tert*-butyldimethylsilyl chloride (354 mg, 2.34 mmol, 1.1 eq.) and imidazole (294 mg, 4.32 mmol) were dissolved in dry DMF (12 mL) and the reaction mixture was left stirring overnight at room temperature. The solvent was reduced *in vacuo* and the resulting residue was purified by flash column chromatography (0-30% Acetone/CH_2Cl_2) to yield the desired, mono-protected species (523 mg, 0.773 mmol. 36%), the di-protected species (200 mg, 0.253 mmol, 11%) as well as unreacted starting material (594 mg, 1.06 mmol, 50%).

Di-TBDMS-L-Valinol-PDI 2

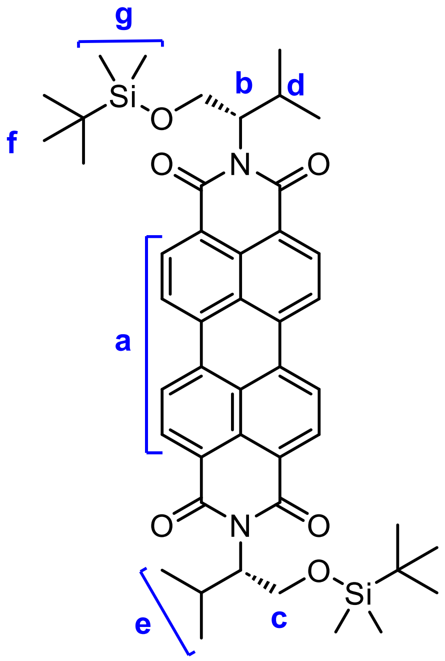

$\mathbf{R_f}$ (SiO_2, 2% Acetone/CH_2Cl_2) 0.63.

^{1}H-NMR (400 MHz, $CDCl_3$) δ/ppm 8.54 (apparent dd, *J* = 31.3, 7.9 Hz, 4H, CH_{Ar}, **a'**), 8.38 (d, *J* = 8.0 Hz, 4H, CH_{Ar}, **a''**), 5.02 (ddd, *J* = 10.6, 9.5, 5.0 Hz, 2H, C-H imide, **b**), 4.38 (t, *J* = 9.9 Hz, 2H, diastereotopic CH_2, **c'**), 4.07 (dd, *J* = 10.4, 5.0 Hz, 2H, diastereotopic CH_2, **c''**), 2.67 (dp, *J* = 10.6, 6.7 Hz, 2H, *i*Pr C-H, **d**), 1.13 (d, *J* = 6.6 Hz, 6H, *i*Pr CH_3, **e'**), 0.92 (d, *J* = 6.7 Hz, 6H, *i*Pr CH_3, **e''**), 0.67 (s, 18H, silyl-*t*Bu CH_3, **f**), -0.01 (s, 6H, silyl-CH_3, **g'**), -0.10 (s, 6H, silyl-CH_3, **g''**).

^{13}C-NMR (101 MHz, $CDCl_3$) δ/ppm 164.74, 163.61, 134.32, 131.78, 130.89, 129.45, 126.26, 123.83, 123.29, 122.91, 62.37, 61.63, 27.53, 25.76, 20.99, 20.43, 18.07, -5.27, -5.45.

HRMS (ESI$^+$) found 791.3896 $[M+H]^+$, $[H_{59}C_{46}N_2O_6Si_2]^+$ requires 791.3906.

UV/Vis ($CHCl_3$) λ/nm (ε/mM^{-1} cm^{-1}) 525 (58), 489 (35), 458 (13)

Mono-TBDMS-L-Valinol-PDI 3

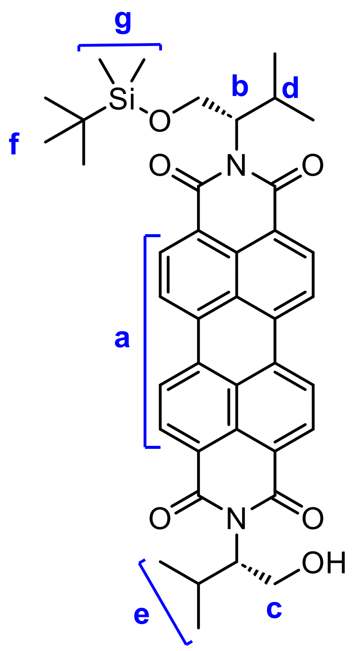

R*f* (SiO_2, 2% Acetone/CH_2Cl_2) 0.14.

^{1}H-NMR (300 MHz, $CDCl_3$) δ/ppm 8.75 – 8.40 (m, 8H, **a**), 5.03 (ddt, J = 19.7, 9.8, 5.1 Hz, 2H, **b**), 4.36 (dd, J = 12.2, 7.8 Hz, 2H, **c'**), 4.06 (td, J = 12.1, 11.7, 3.8 Hz, 2H, **c''**), 3.00 – 2.52 (m, 2H, **d**), 1.17 (dd, J = 16.2, 6.6 Hz, 6H, **e'**), 1.02 – 0.77 (m, 6H, **e''**), 0.67 (s, 9H, **f**), -0.01 (s, 3H, **g'**), -0.10 (s, 3H, **g''**).

^{13}C-NMR (126 MHz, $CDCl_3$) δ/ppm 164.89, 164.80, 163.68, 135.01, 134.19, 131.88, 131.02, 129.61, 129.52, 126.42, 126.34, 124.11, 123.59, 123.39, 123.29, 123.05, 62.78, 62.50, 62.34, 62.04, 27.64, 26.17, 25.78, 21.01, 20.69, 20.53, 20.49, 20.17, 20.07, 18.08, -5.27, -5.43.

HRMS (ESI$^+$) found 677.3035 $[M+H]^+$, $[H_{44}C_{40}N_2O_6Si]^+$ requires 677.3042.

Di-TBDMS-LValinol-PDI Malonate 4

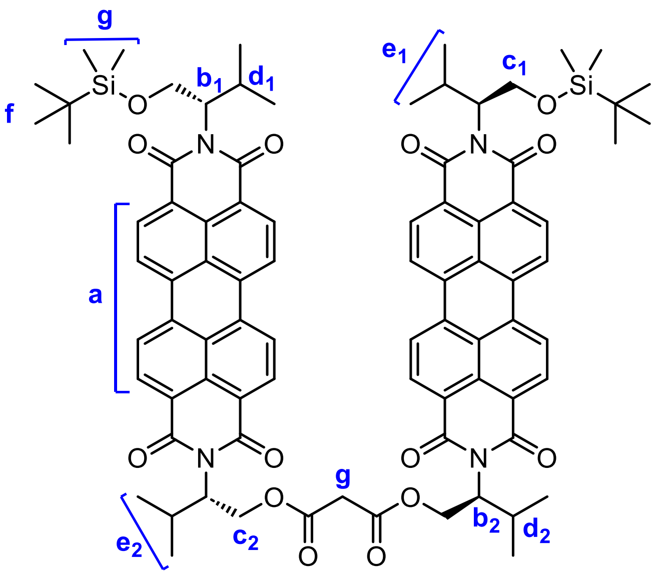

To a dry round-bottom flask equipped with a stirrer bar was added mono-TBS-L-Valinol-PDI **3** (387 mg, 572 µmol), dry pyridine (460 µL, 10 eq.) and dry CH_2Cl_2 (15 mL) and the reaction flask cooled with ice. To this was added dropwise a solution of malonyl chloride (28 µL, 0.5 eq.) in CH_2Cl_2 (2 mL). The reaction was then allowed to warm up to room temperature and left stirring overnight. The solvent was then removed in vacuo and the resulting residue purified by flash column chromatography (SiO_2,

2% Acetone/$CHCl_3$) to yield a red solid as the desired compound (162 mg, 114 µmol, 20%).

R_f (SiO_2, 2% Acetone/$CHCl_3$) 0.53.

^{1}H-NMR (500 MHz, Chloroform-*d*) δ/ppm 8.69 – 8.18 (m, 16H, **a**), 5.02 (tt, *J* = 10.0, 4.9 Hz, 4H, **b**), 4.78 (s, 2H, **C_2'**), 4.55 (s, 2H, **C_2''**), 4.44 – 4.29 (m, 2H, **C_1'**), 4.08 (m, 2H, **C_1''**), 3.23 (s, 2H, **g**), 2.74 – 2.53 (m, 4H, **d**), 1.12 (dd, *J* = 28.1, 7.2 Hz, 12H, **e**), 0.98 – 0.78 (m, 12H, **e**), 0.67 (s, 18H, **f**), 0.03 – -0.05 (m, 6H, **g'**), -0.10 (d, *J* = 9.9 Hz, 6H, **g''**).

^{13}C-NMR (101 MHz, $CDCl_3$) δ/ppm 166.11, 164.74, 164.40, 163.55, 134.68, 134.21, 132.04, 131.82, 131.24, 130.95, 129.58, 129.45, 126.35, 126.26, 123.99, 123.69 – 122.47 (multiple broad carbon signals overlapping), 64.69, 62.42, 61.67, 58.80, 41.52, 27.70, 27.60, 25.78, 21.01, 20.65, 20.47, 19.94, 18.10, -5.24, -5.42.

HRMS (ESI$^+$) found 1421.5814, $[C_{74}H_{61}N_4O_{16}]^+$ requires 1421.5909.

UV/Vis ($CHCl_3$) λ/nm (ε/mM^{-1} cm^{-1}) 527 (118), 490 (78), 459 (29)

L-Valinol-Perylene-Diimide-Malonate Macrocycle 5

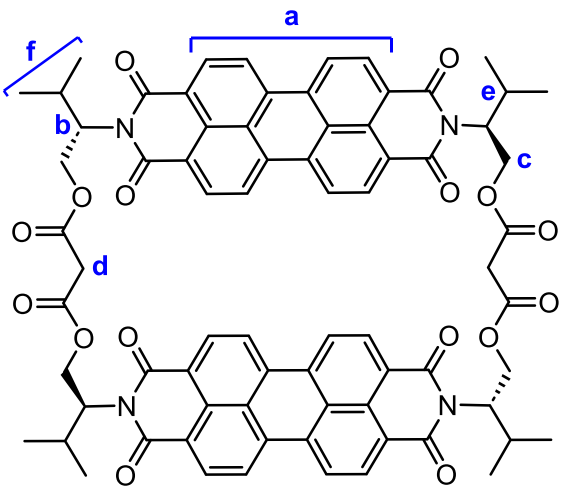

To a dry round-bottom flask equipped with a stirrer bar was added L-Valinol-PDI **1** (1 g, 1,78 mmol), dry pyridine (7 mL, 50 eq.) and dry dichloromethane (1.5 L). A solution of malonyl chloride (173 µL, 1 eq.) in dry dichloromethane (5 mL) was added via syringe pump over 8 h and the reaction left stirring overnight. The resulting solution was reduced in vacuo and the residue purified by flash column chromatography (SiO_2, 4-10% Acetone/CH_2Cl_2), and the fractions containing the desired macrocycle further purified by preparative TLC (4% Acetone/CH_2Cl_2) to yield a red solid as the desired compound (70 mg, 0.055 mmol, 6.2%).

R_f (SiO_2, 8% Acetone/CH_2Cl_2) 0.21.

^{1}H-NMR (500 MHz, $CDCl_3$) δ/ppm 8.74 (d, J = 7.9 Hz, 4H), 8.65 – 8.57 (m, 8H), 8.55 (d, J = 8.0 Hz, 4H), 4.96 (dd, J = 11.7, 7.9 Hz, 8H, b, c’), 4.65 (d, J = 8.2 Hz, 4H, c’’), 3.16 (s, 4H, d), 2.89 – 2.55 (m, 4H, e), 1.22 (d, J = 6.6 Hz, 12H, f’), 0.86 (d, J = 6.7 Hz, 12H, f’’).

^{13}C-NMR (126 MHz, $CDCl_3$) δ/ppm 165.97, 164.31, 163.60, 134.61, 134.49, 132.46, 130.91, 129.74, 126.54, 123.54, 123.31, 123.05, 122.94, 64.61, 59.52, 42.05, 27.43, 20.87, 19.92.

HRMS (ESI$^+$) found 1261.4050 $[M+H]^+$, $[H_{75}C_{60}N_4O_{16}]^+$ requires 1261.4077, and also found 1283.8888 $[M+Na]^+$, $[H_{74}C_{60}N_4O_{16}Na]^+$ requires 1283.3896.

IR (powder, cm^{-1}) 2962 (w), 1735 (m, C=O), 1692 (s, C=O), 1654 (s, C=O), 1592 (s, C=O), 1577 (s, C=O), 1405 (m), 1337 (s), 1246 (s), 810 (s), 747 (s).

UV/Vis ($CHCl_3$) λ/nm (ε/mM^{-1} cm^{-1}) 526 (88), 491 (75), 461 (31)

D-Valinol-Perylene Diimide (PDI) 6

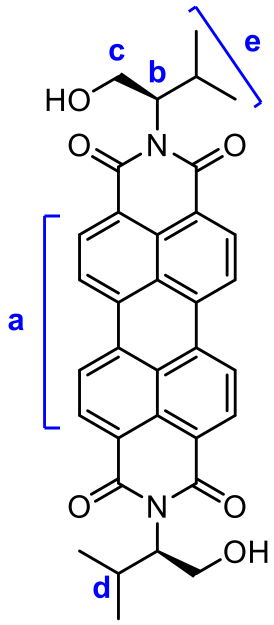

Synthesis analogous to PDI **1**: To a round-bottom flask under N_2 atmosphere was added perylene tetracarboxylic acid dianhydride (1 g, 2.55 mmol), D-valinol (539 mg, 5.23 mmol), 1,4-dioxane (10 mL) and dimethylacetamide (10 mL). The reaction mixture was stirred at 140 °C for 24 h. The mixture was then cooled, and the solvent reduced *in vacuo*. The resulting residue was purified by flash column chromatography (SiO_2, 4% MeOH/CH_2Cl_2) to yield a dark red/brown solid as the title compound (713 mg, 1.27 mmol, 50%).

^{1}H-NMR (400 MHz, $CDCl_3$): δ/ppm 8.18 (dd, *J* = 129.3, 75.3 Hz, 8H), 5.09 (td, *J* = 9.9, 3.7 Hz, 2H), 4.51 (s, 2H), 4.17 (s, 2H), 2.65 (s, 2H), 1.17 (d, *J* = 6.5 Hz, 6H), 0.81 (s, 6H).

HRMS (ESI$^+$) found 563.2186 $[M+H]^+$, $[H_{35}C_{30}N_2O_6]^+$ requires 563.2176.

D-Valinol-Perylene-Diimide-Malonate Macrocycle 7

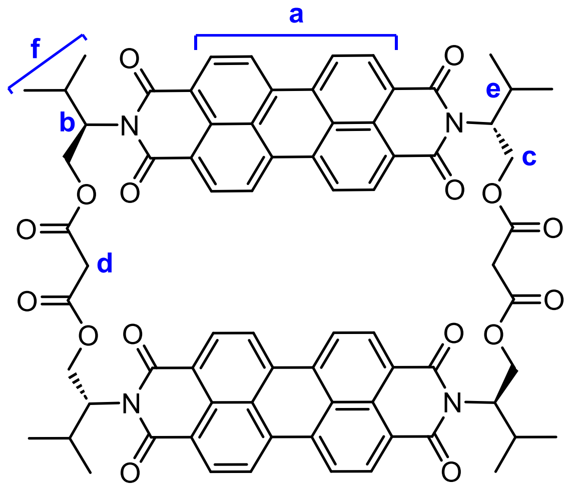

To a dry round-bottom flask equipped with a stirrer bar was added D-Valinol-PDI **1** (500 mg, 0.89 mmol), dry pyridine (2.15 mL, 30 eq.) and dry dichloromethane (1 L). A solution of malonyl chloride (86 µL, 1 eq.) in dry dichloromethane (5 mL) was added via syringe pump over 8 h and the reaction left stirring overnight. The resulting solution was reduced *in vacuo* and the residue purified by flash column chromatography (SiO_2, 4-10% Acetone/CH_2Cl_2), and the fractions containing the desired macrocycle further purified by preparative TLC (4% Acetone/CH_2Cl_2),and purified further by column chromatography (4% Acetone/CH_2Cl_2) to yield a red solid as the desired compound (12 mg, 9.5 µmol, 2%).

^{1}H-NMR (400 MHz, $CDCl_3$) δ/ppm 8.74 (d, *J* = 7.9 Hz, 4H), 8.68 – 8.57 (m, 8H), 8.55 (d, *J* = 7.9 Hz, 4H), 4.96 (dd, *J* = 12.1, 7.9 Hz, 8H), 4.65 (d, *J* = 8.6 Hz, 4H), 3.16 (s, 4H), 2.75 (s, 4H), 1.22 (d, *J* = 6.6 Hz, 12H), 0.86 (d, *J* = 6.6 Hz, 12H).

^{13}C-NMR (101 MHz, $CDCl_3$) δ/ppm 165.98, 164.32, 163.62, 134.63, 134.51, 132.48, 130.92, 129.76, 126.56, 123.56, 123.32, 123.07, 122.95, 64.63, 59.53, 42.07, 27.44, 20.88, 19.93.

HRMS (ESI$^+$) found 1261.4065 $[M+H]^+$, $[H_{75}C_{60}N_4O_{16}]^+$ requires 1261.4077.

2) Photophysical Properties

General

UV-Visible absorbance spectra were recorded on a Shimadzu UV-3600i Plus Spectrometer, with a wavelength accuracy of ± 0.2 nm in the visible and uv region, a baseline flatness of ± 0.002 nm (200 – 3000 nm) and a noise level of <0.00008 Abs (900 nm).

Fluorescence spectra were recorded on a Cary Eclipse fluorescence spectrometer at medium gain and smoothed using a Savitzky-Golay filter.

Circular Dichroism measurements were taken on a JASCO J-1500 CD Spectrophotometer with a wavelength accuracy ± 0.2 nm (250 to 500 nm), ± 0.5 nm (500 to 800 nm), a photometric accuracy of ± 0.01 Abs and a CD root mean square noise < 0.007 mdeg (500 nm). "Recorded traces were then baseline corrected and smoothed using the provided software.

Fluorescence Spectra of 2, 4 and 5 in $CHCl_3$

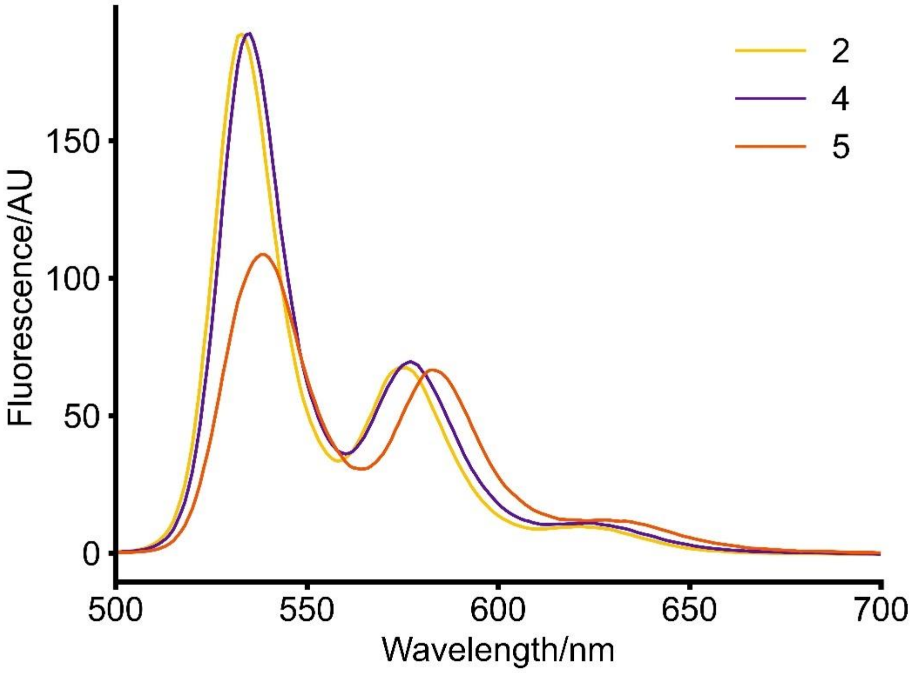

Supplementary Figure 2-1: Fluorescence Spectra of Compounds **2**,**3** and **5** in $CHCl_3$, recorded at 125 nM concentration at 600 V gain excited at 450 nm.

Absorbance and Fluorescence Spectra of 2, 4 and 5 in PhMe

Absorbance spectra were recorded at 5 µM concentration in a 1 cm pathlength cuvette. Fluorescence measurements were recorded at 250 nM concentration.

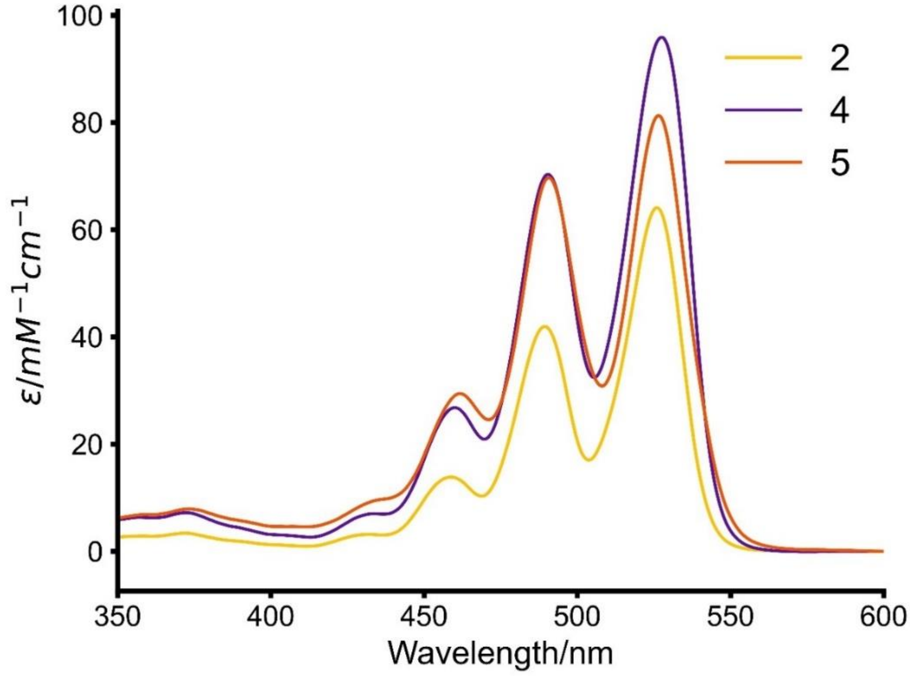

Supplementary Figure 2-2: Absorbance Profiles of Compounds **2**, **4** and **5** in PhMe, taken at 5 µM concentration at 1 cm pathlength.

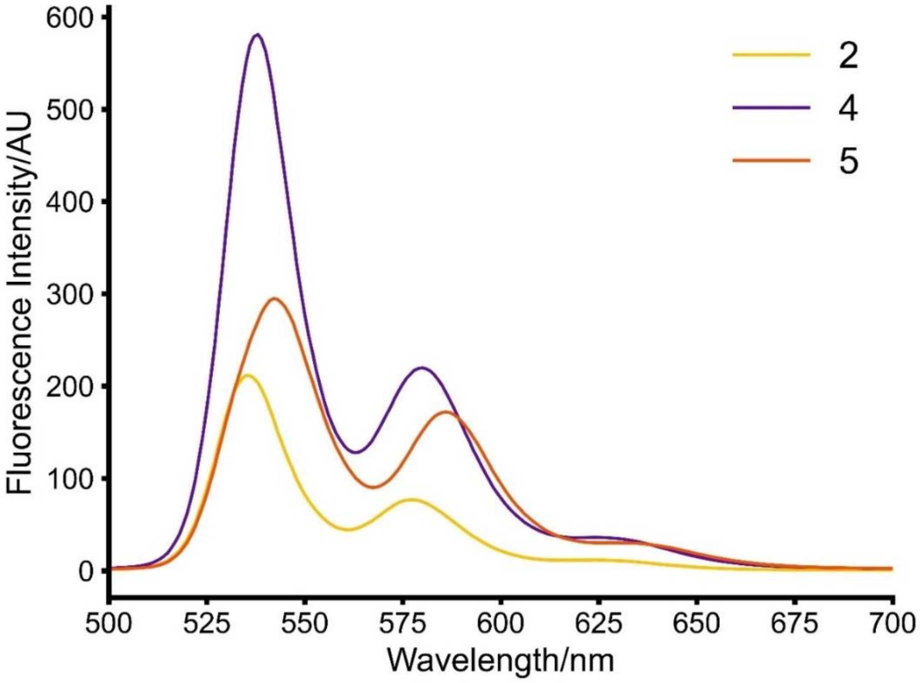

Supplementary Figure 2-3: Fluorescence Spectra of Compounds **2**,**3** and **5** in PhMe, recorded at 250 nM concentration at 600 V gain, excited at 450 nm.

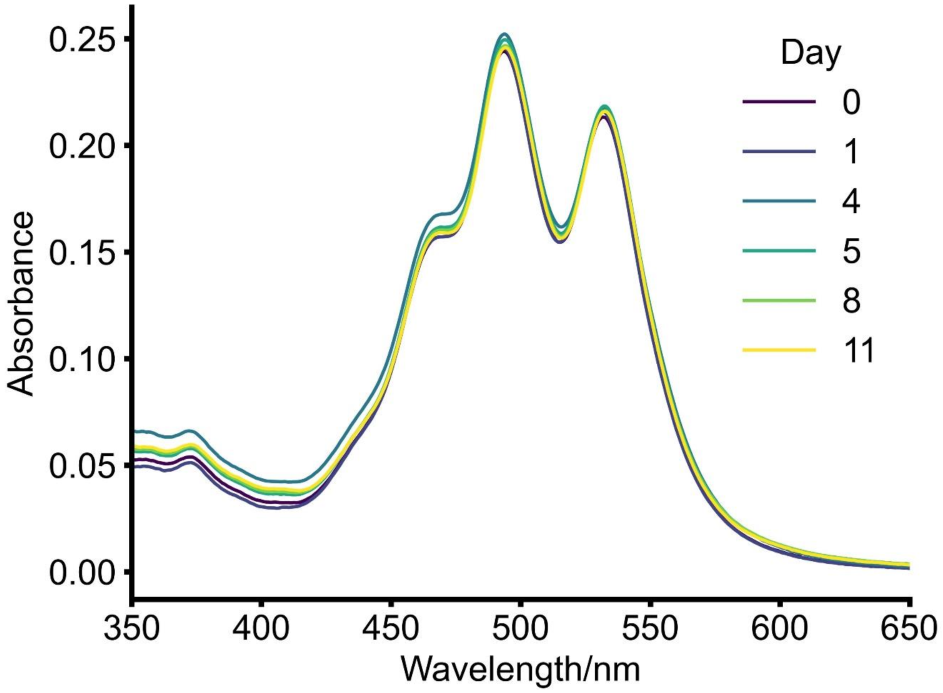

Supplementary Figure 2-4: Stability of solutions of macrocycle **5** in 0.1% DMSO in H_2O over several days.

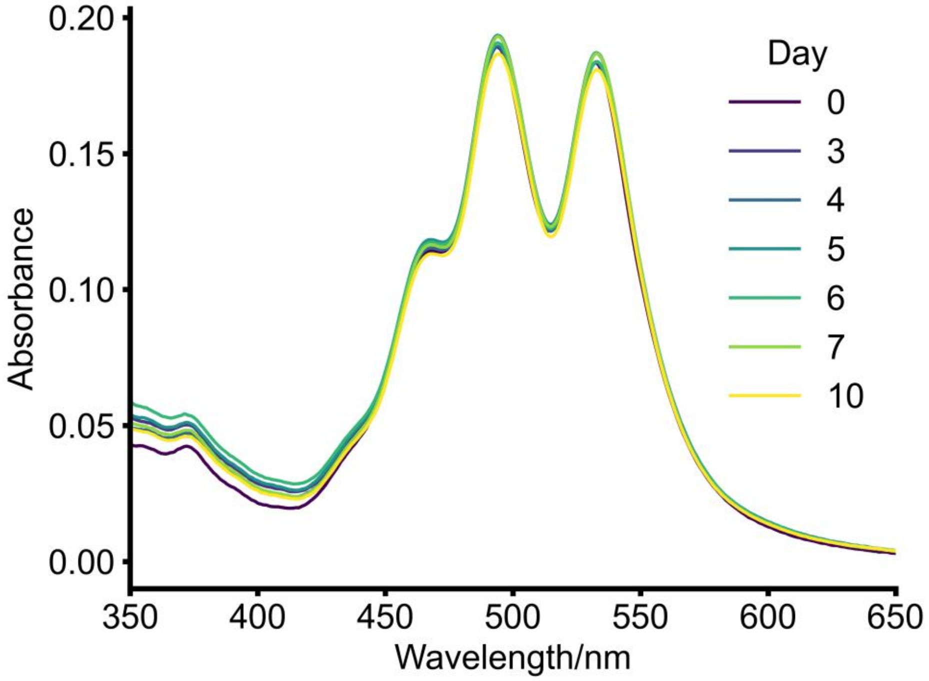

Supplementary Figure 2-5: Stability of solutions of macrocycle **5** in 0.1% THF in H_2O over several days.

Measurement of Linearity over a Range of Concentrations in H_2O from DMSO

Measurements were taken in a 0.1 cm pathlength cuvette over a range of 11-300 µM through dilution. For solubility reasons, the amount of DMSO had to be increased to 6%. The resulting data was tested for linearity by plotting absorbance vs. concentration (*vide infra*). A trendline fitted to the 6 datapoints of lowest concentration show a deviation of linearity above 100 µM. This indicates that at 10 µM, any aggregation behaviour observed is intra-, rather than intermolecular.

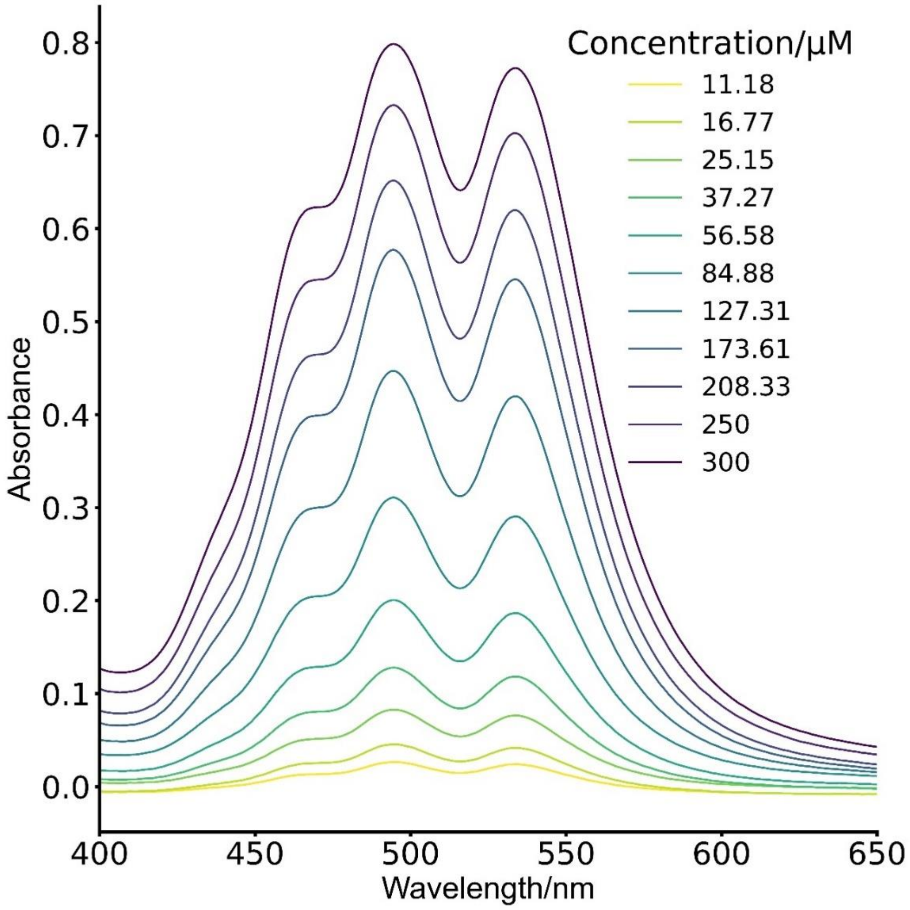

Supplementary Figure 2-6: Absorbance Plot of Macrocycle **5** in 6% DMSO/H_2O over different concentrations.

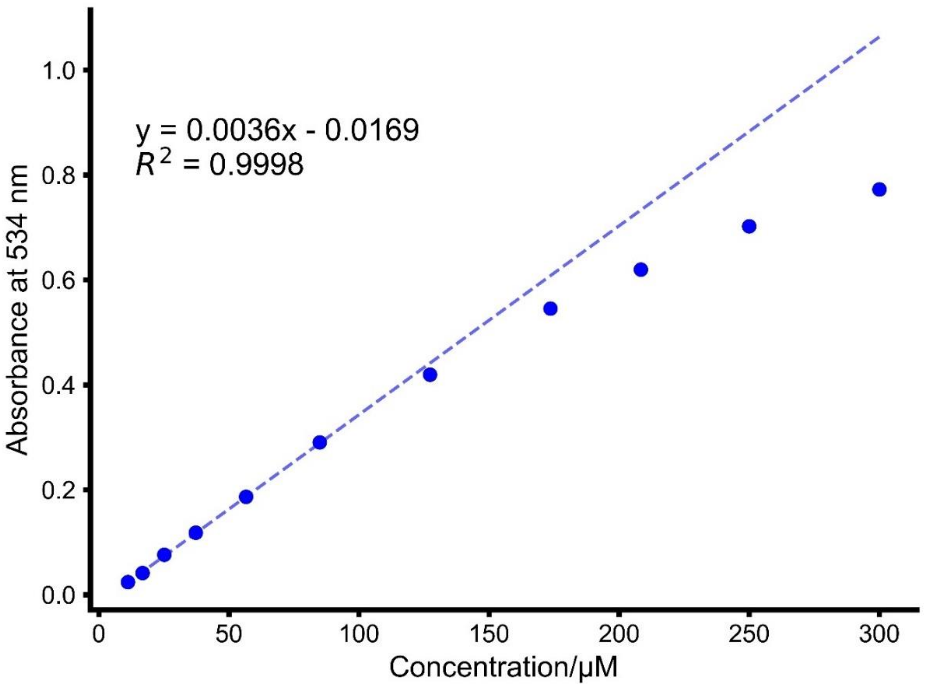

Supplementary Figure 2-7: Beer-Lambert Plot of **Supplementary Figure 2-6**, plotting concentration (in µM) vs. absorbance at the 0-0 transition (λ = 534 nm). A trendline fitted to the first 6 datapoint shows the expected linearity when extrapolated (as all measurements are taken below 1 Absorbance, where the Beer-Lambert Law can be approximated as linear), with the real data showing a deviation past 100 µM. Aggregation observed in absorbance measurements at low concentrations (<100 µM) is therefore intramolecular.

Measurement of Linearity over a Range of Concentrations in H_2O from THF

Measurements were taken over a range of 5-100 µM at 3% THF in a 0.1 cm pathlength cuvette, except the 5 µM , due to the detection limit of the instrument. Therefore, the Y-axis is given as absorbance divided by pathlength. The resulting data was tested for linearity by plotting absorbance vs. concentration (*vide infra*).

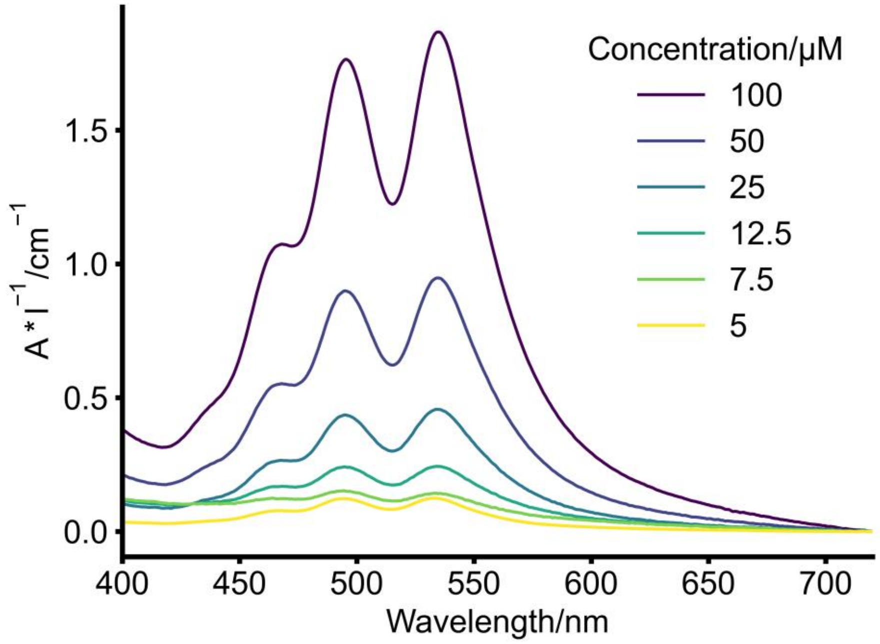

Supplementary Figure 2-8*:* Absorbance plot corrected for pathlength, of Macrocycle **5** in 3% THF/H_2O over different concentrations. Samples were measured at 0.1 cm pathlength, except the 5 µM sample, which had to be measured at 1 cm pathlength, due to the detection limit of the instrument. Therefore, the Y-axis is given as absorbance divided by pathlength.

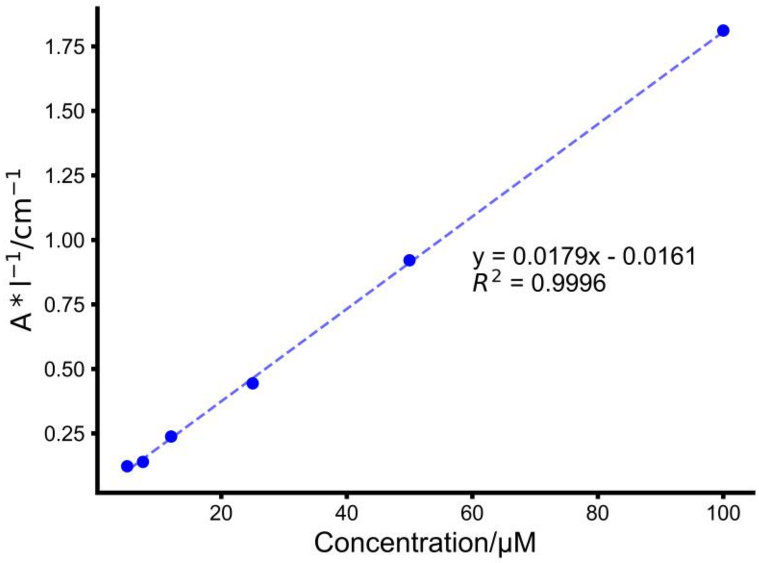

Supplementary Figure 2-9: Beer-Lambert Plot of **Supplementary Figure 2-8**, plotting concentration (in µM) vs. absorbance divided by pathlength at the 0-0 transition (λ = 531 nm). A trendline shows the expected linearity. The compound was found not to be soluble above 100 µM.

Quantum Yield and Lifetime Determination

Quantum yields and lifetimes were measured on an Edinburgh instruments FLS1000 spectrometer. Quantum yields were recorded as Φ_{obs} with 0.1 nm step size and 0.2 seconds dwell time using an integrating sphere and a Visible PMT-980. Measurements were taken at 1 µM concentration with 3 nm bandwidth at the excitation wavelength and 0.1 nm emission bandwidth, and intensity optimised to yield 10^6 counts per second for the blank sample at the excitation wavelength. Samples in $CHCl_3$ and PhMe were excited at 491 nm and the emission measurement was from 471-750 nm. Samples in H_2O were excited at 493 nm, and the emission measurement was from 473-850 nm. Samples with coronene were excited at 491 nm and emission data was integrated from 600-750 nm. Data was processed using the Fluoracle software. Lifetimes were recorded using a pulsed 375 nm laser using a 5 µM solution of compound **5** in either $CHCl_3$ or a 3 µM Solution 0.01% DMSO/H_2O and data fitted using Fluoracle.

Tabulated Quantum Yields

**Index**	**Species**	**Solvent**	**λ_{ex}/nm**	**Quantum Yield/%**
**1**	Macrocycle **5**	$CHCl_3$	491	91
**2**	Macrocycle **5**	PhMe	491	87
**3**	Macrocycle **5**	H_2O	493	7
**4**	Macrocycle **5** + xs. Coronene	$CHCl_3$	491	7
**5**	Macrocycle **5** + xs. Coronene	PhMe	491	14

Supplementary Table 2-1: Measured Quantum Yields for Macrocycle **5** in various solvents without and with guest species.

Lifetimes of macrocycle in $CHCl_3$ and H_2O

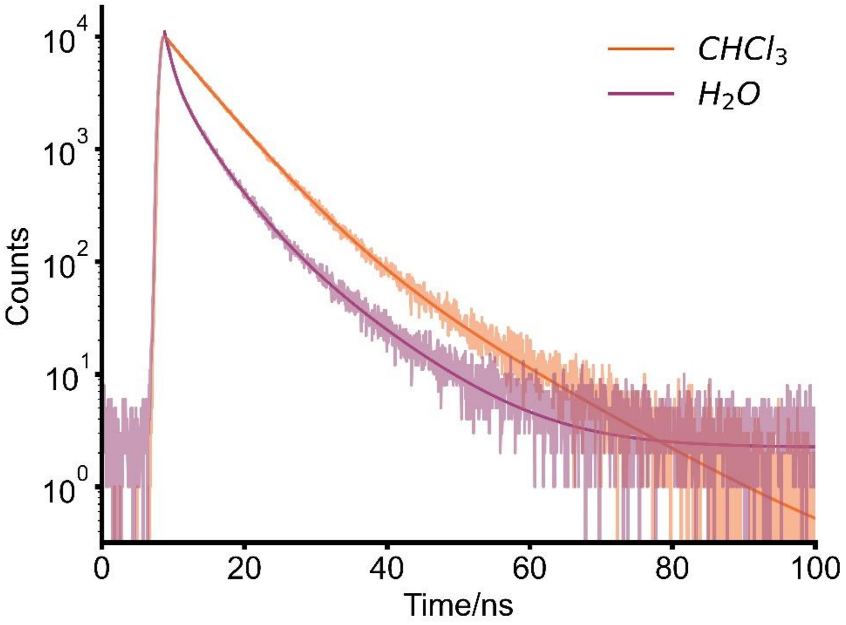

Supplementary Figure 2-10: Lifetimes of ValPDIMC **5** in $CHCl_3$ and H_2O and their calculated fits. $CHCl_3$ was measured at 5 µM concentration, H_2O was measured at 3 µM concentration with 0.03% DMSO present. Fits correspond to values given in **Supplementary Table 2-2**.

**Sample**	**A**	**B_1**	**τ_1**	**B_2**	**τ_2**	**B_3**	**τ_3**	**X^2**	**$\langle\tau\rangle_{amp}$**	**$\langle\tau\rangle_{int}$**
**$CHCl_3$**	0.12	9470.03	5.52	642.478	12.39	-	-	1.04	5.96	6.43
**H_2O**	2.23	6736.46	1.02	3972.96	3.86	654.98	9.123	1.34	2.48	4.29

Supplementary Table 2-2: Fitting Parameters used on Lifetime data recorded for Macrocycle **5** in $CHCl_3$ and H_2O. Data was fitted to the equation:

$$I(t) = A + B_1 \cdot e^{\left(\frac{-t}{\tau_1}\right)} + B_2 \cdot e^{\left(\frac{-t}{\tau_2}\right)} + B_3 \cdot e^{\left(\frac{-t}{\tau_3}\right)}$$

Aggregation Study from DMSO to H_2O

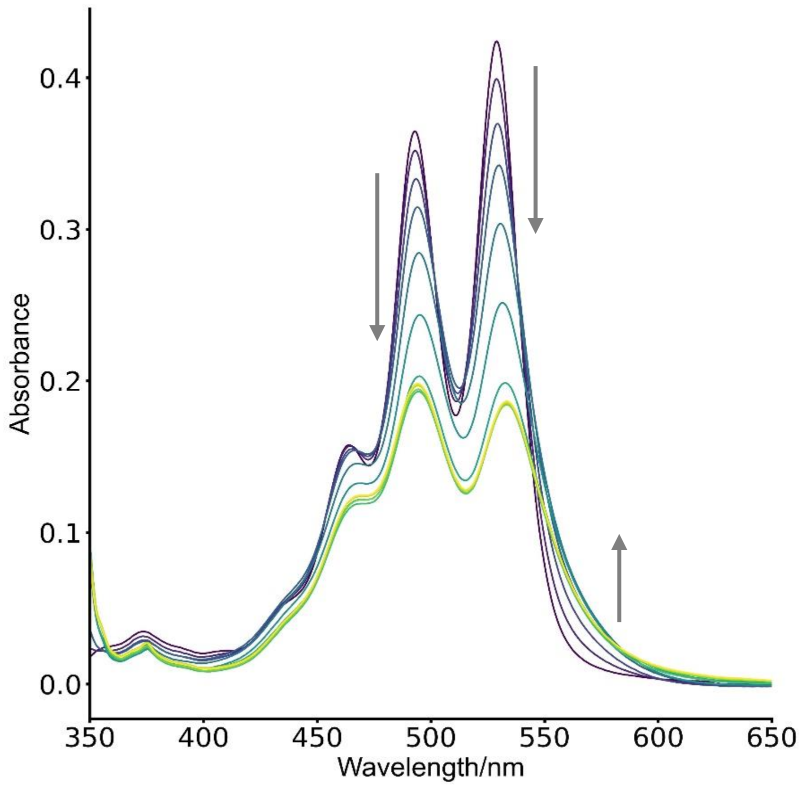

Supplementary Figure 2-11: UV-Visible absorbance of **5** from 0-50% DMSO. Experiment was performed at 5 µM concentration in a 1 cm pathlength cuvette.

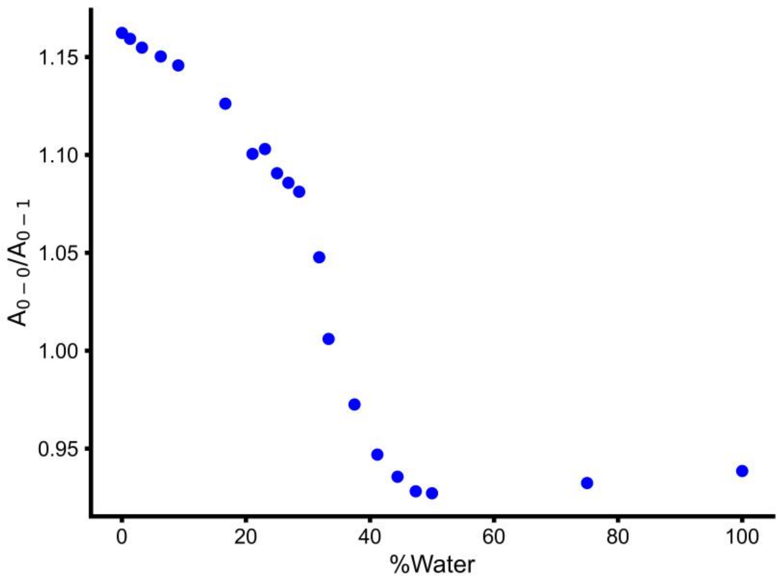

Supplementary Figure 2-12: 0-0/0-1 Ratio of **Supplementary Figure 2-11** against %Water. Datapoints for 75 and 100% were taken separately.

A Gibbs free energy of intramolecular H-type aggregation (ΔG_{agg}) was determined for each solvent, following an adapted method used by Würthner and co-workers.[2]

Using the assumptions that The A_{0-0}/A_{0-1} ratio in DMSO corresponds to all molecules of macrocycle **5** being in a state of no H-type aggregation, and the ratio corresponding to 50% DMSO/H_2O corresponds to all molecules in an H-aggregated state.

Therefore, the mole fraction α_u of fully unaggregated molecules can be estimated according to the following equation:

$$\alpha_u = \frac{\rho_a - \rho_{obs}}{\rho_a - \rho_u}$$

Where ρa is the A_{0-0}/A_{0-1} ratio of the fully H-type aggregated macrocycle in 50% DMSO/H_2O, ρu is the ratio of the non-aggregated macrocycle in DMSO and ρobs is the observed ratio for intermediate DMSO:H_2O ratios.

From this, an equilibrium constant Keq can be calculated as follows:

$$K_{eq} = \frac{1 - \alpha_u}{\alpha_u}$$

And ΔG_{agg} can then be determined for each solvent according to:

$$\Delta G = -RT \ln K$$

A plot of the linear regime (excluding asymptotic behaviour, which is not represented by this model) allows for determination of ΔG_{agg} as the intercept of the resulting trendline.

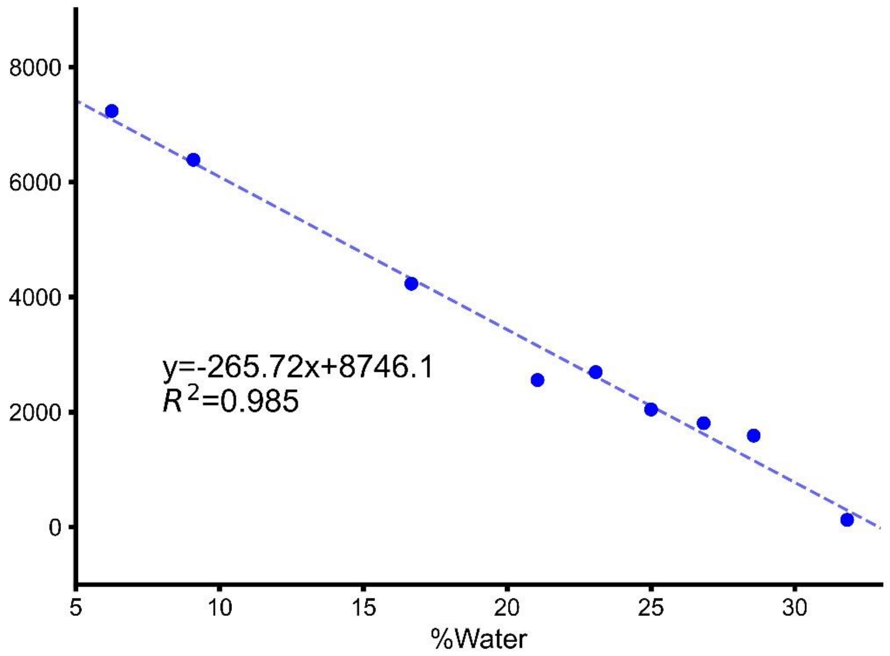

Supplementary Figure 2-13: Calculation of the Gibbs free energy of aggregation from the data obtained in **Supplementary Figure 2-11 and 2-12**. Blue datapoints were included in the fit, whereas grey datapoints were omitted, due to deviating behaviour, which is not included in the model.

Absorption Spectra of 5 in various solvents

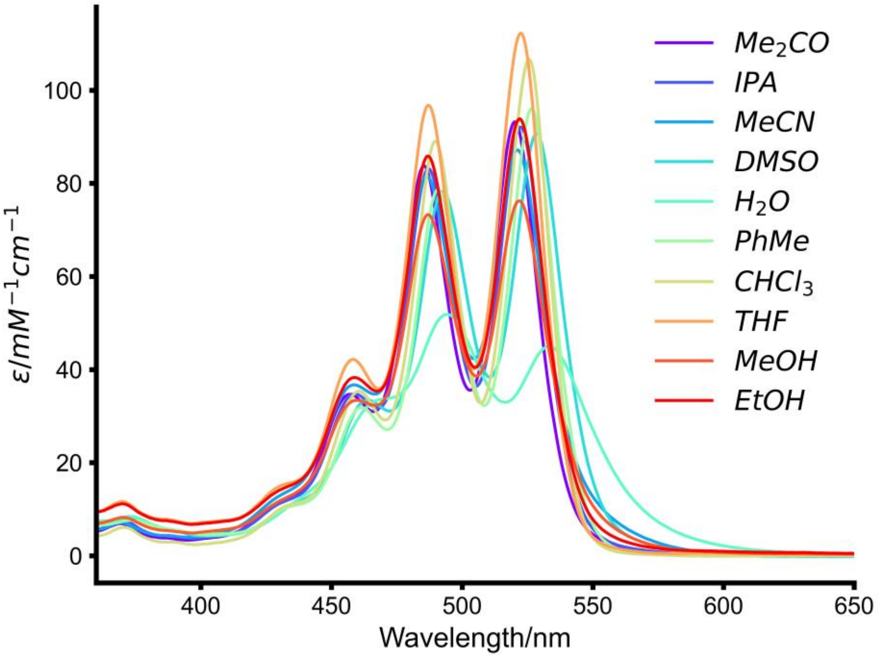

Supplementary Figure 2-14: Absorption spectra of **5** in polar solvents that follow the polar line of the Reichardt $E_T(30)$ polarity scale, all with 0.1% DMSO. Samples were prepared by addition of a 5 mM stock solution of the macrocycle in DMSO into the desired solvent to 5 µM and measured at 1 cm pathlength.

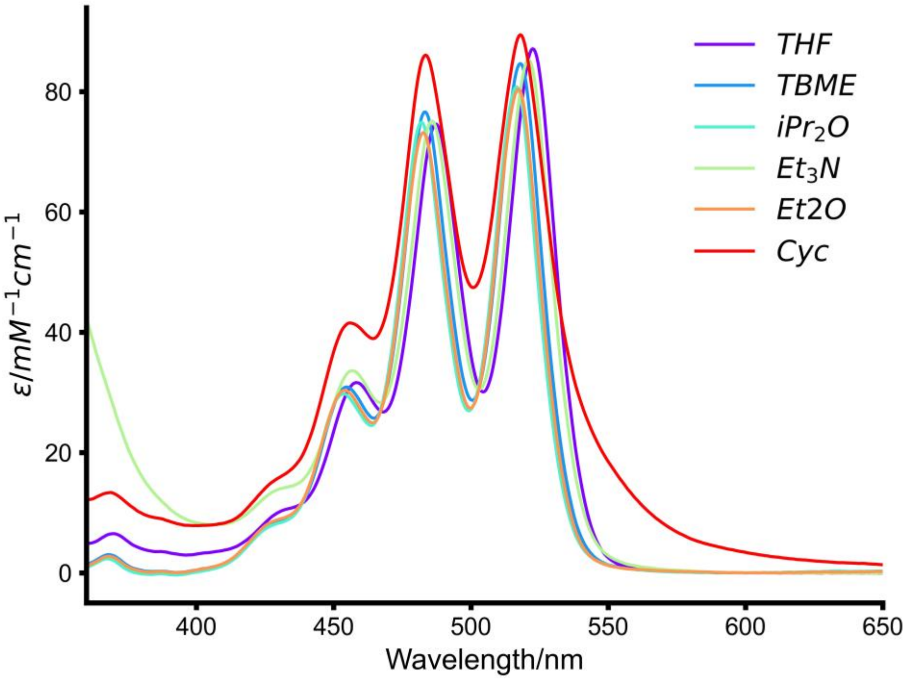

Supplementary Figure 2-15: Absorption spectra of **5** in polar solvents that follow the apolar line of the Reichardt $E_T(30)$ polarity scale, all with 0.1% THF. Samples were prepared by addition of a 5 mM stock solution of the macrocycle in DMSO into the desired solvent to 5 µM and measured at 1 cm pathlength.

Solvent/Solvent Titration of Macrocycle 5 in $CHCl_3$/Cyclohexane

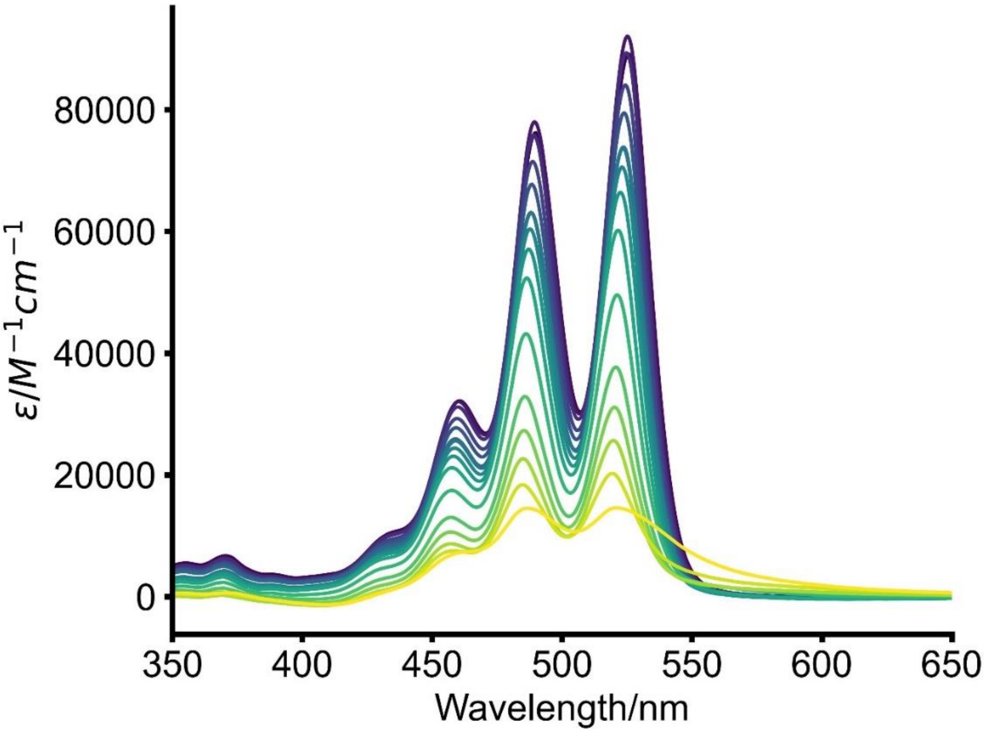

Supplementary Figure 2-16: Change in absorbance spectrum going from pure $CHCl_3$ (purple) to 98% cyclohexane (yellow) at 83 µM concentration, measured in a 0.1 cm cuvette.

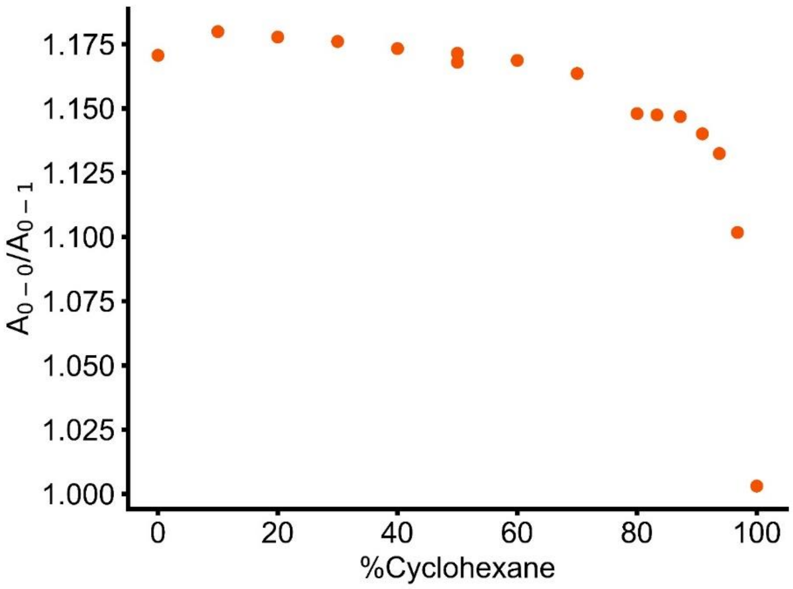

Supplementary Figure 2-17: Ratio of the absorbance of the 0-0 and 0-1 peaks from **Supplementary Figure 2-16** against %Cyclohexane. A decrease in ratio is in accordance with aggregation of the macrocycle.

CD Signals of Compounds 2,3 and 5 in PhMe

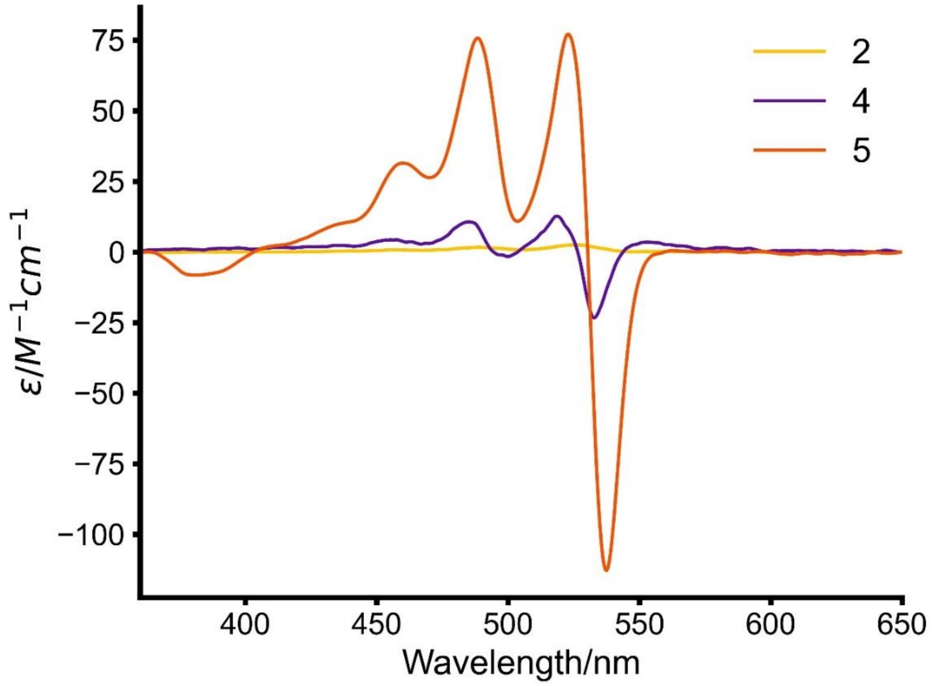

Supplementary Figure 2-18: Circular Dichroism spectra of compounds **2**, **3** and **5** in PhMe, recorded at 150 µM in a 1 mm pathlength cuvette (for **2** and **3**) or at 15 µM in a 10 mm pathlength cuvette (for **5**).

Change in CD signal upon heating

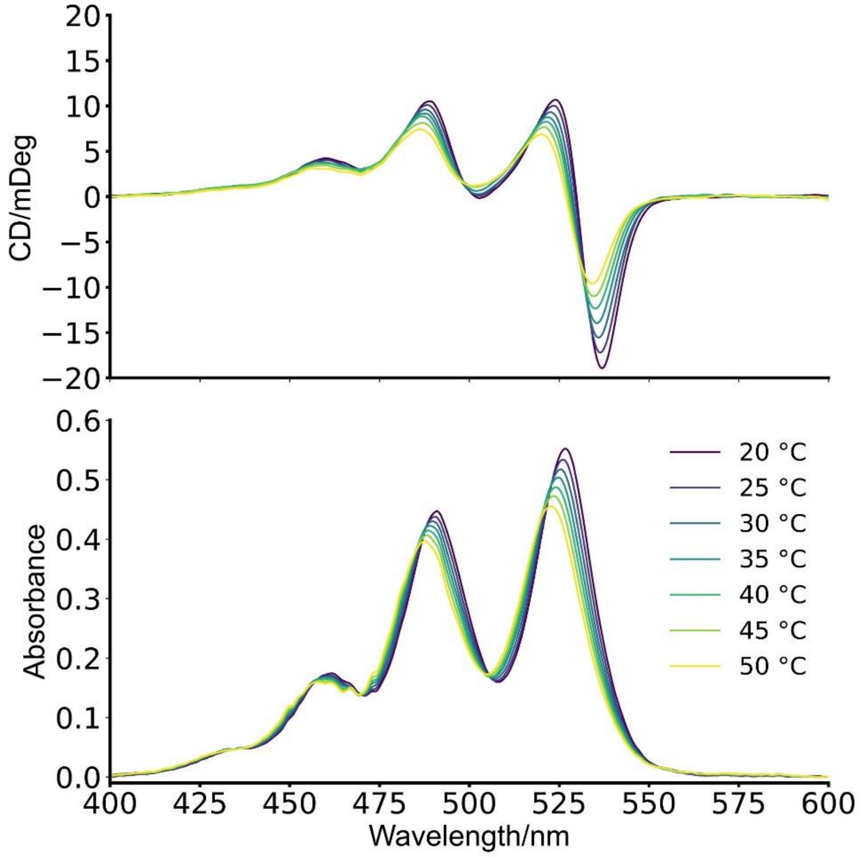

Supplementary Figure 2-19: Change in CD signal and absorbance of **5** upon heating in $CHCl_3$. There is a visible loss in CD intensity upon heating of the sample, indicating the population of higher energy states of lower helicity.

CD in different solvents

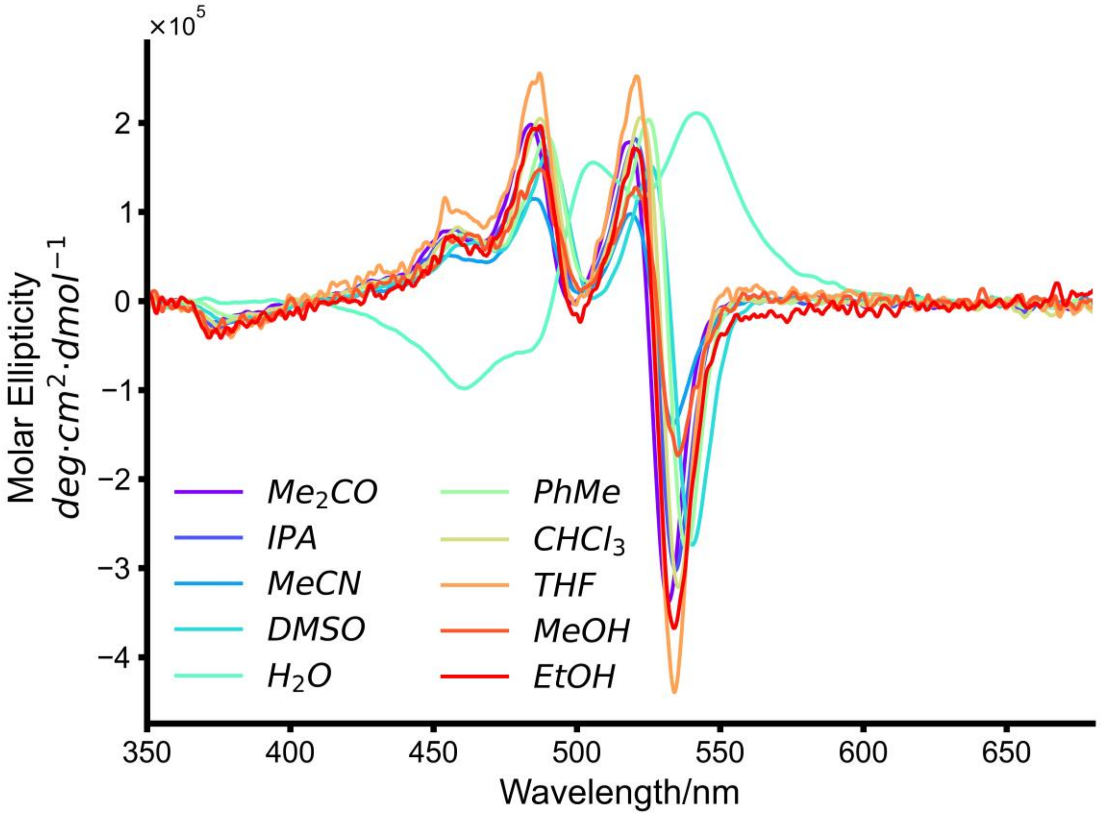

Supplementary Figure 2-20: Changes in Circular Dichroism of **5** in various solvents. Samples were prepared by addition from a 5 mM stock solution in DMSO into the appropriate solvent for a final concentration of 5 µM, with 0.1% DMSO.

Calculated g_{abs} in different solvents

Index	Solvent	g_{abs} (x 10^{-3})	Wavelength/nm
**1**	Me_2CO	−2	535
**2**	IPA	−2	537
**3**	MeCN	−2	544
**4**	DMSO	−1	536
**5**	H_2O	+2	551
**6**	PhMe	−2	541
**7**	$CHCl_3$ (S_0-S_1)	−2	539
**8**	$CHCl_3$ (S_0-S_2)	−2	375
**9**	THF	−3	537
**10**	MeOH	−1	537
**11**	EtOH	−3	538

Supplementary Table 2-3: Calculated g_{abs} in different solvents. The largest error in g_{abs} is ±2 x 10^{-4} for all S_0-S_1 transitions (±6 x 10^{-4} for S_0-S_2), which was calculated using error propagation from given experimental errors of the equipment used in determining this data.

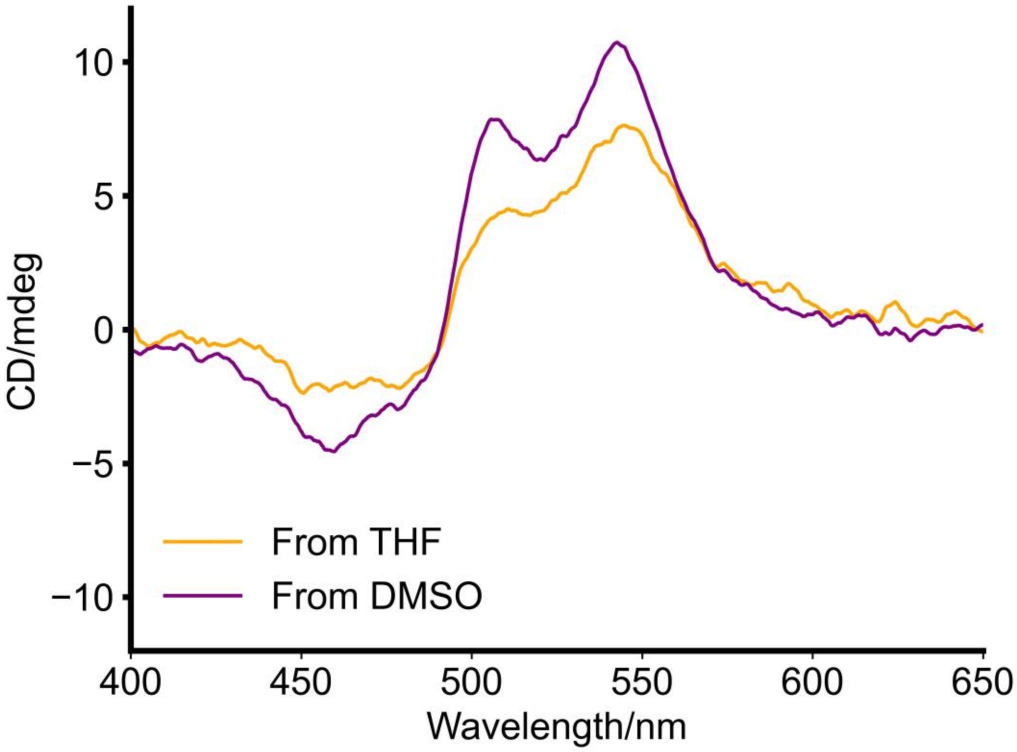

Supplementary Figure 2-21: Comparison of CD spectra in H_2O from THF and DMSO (at 0.1%) recorded at 5 µM concentration.

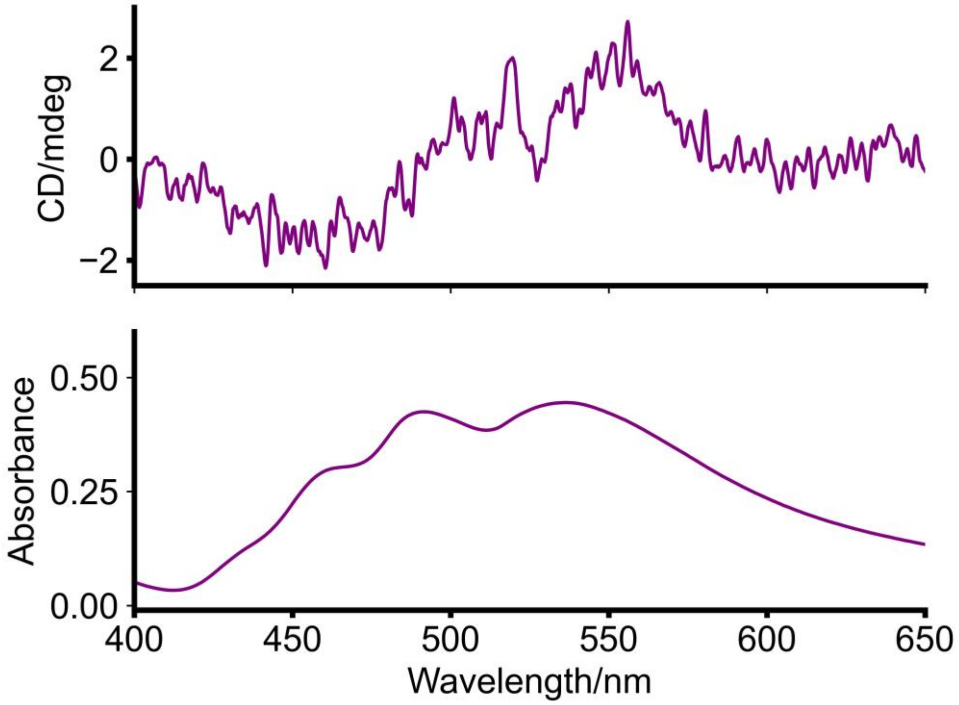

Supplementary Figure 2-22: Circular dichroism and corresponding absorbance trance of macrocycle **5** in 98% cyclohexane with 2% THF recorded in a 0.1 cm pathlength cuvette.

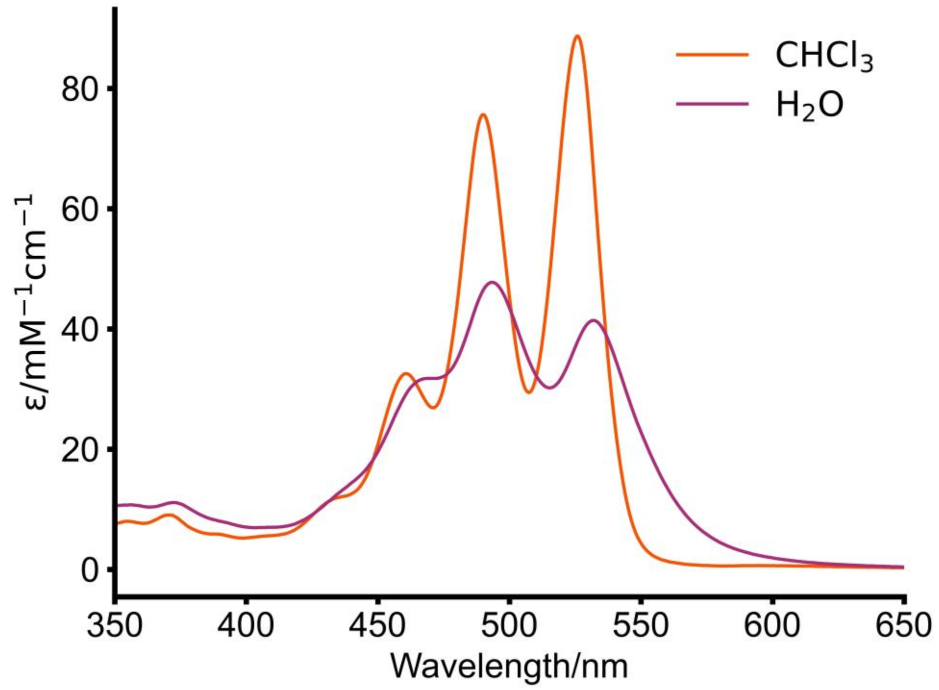

Supplementary Figure 2-23: Absorbance spectra of D-valinol-derived Macrocycle **7** in $CHCl_3$ and H_2O, recorded at 5 µM concentration with 0.1% DMSO.

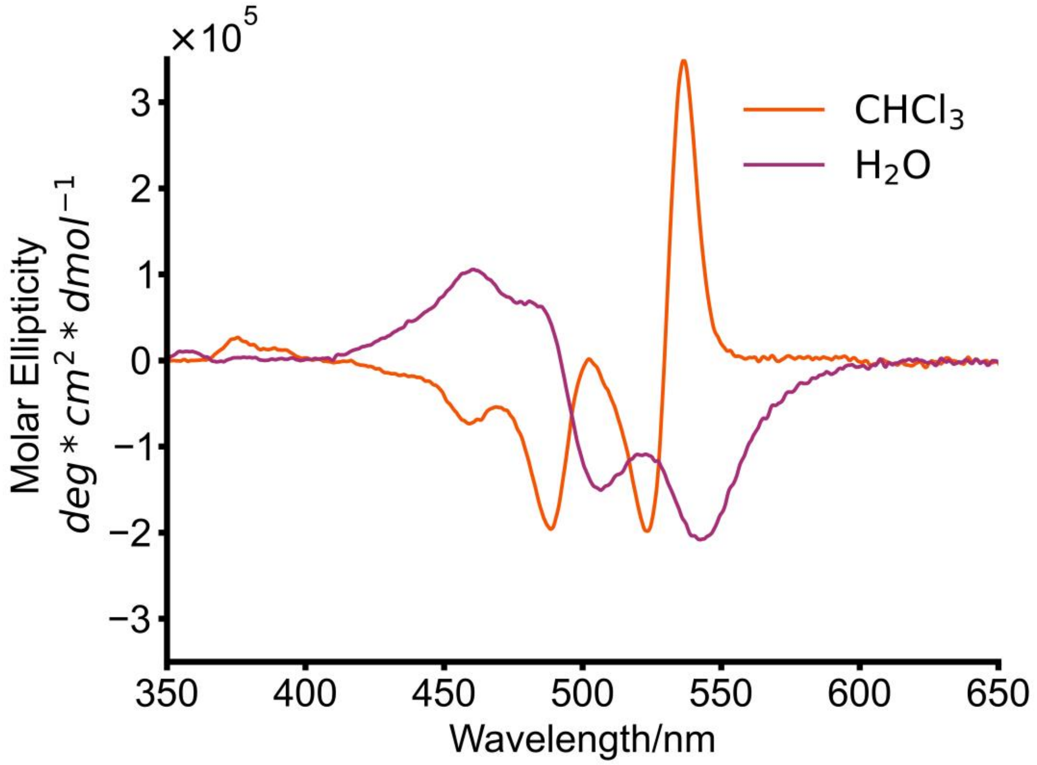

Supplementary Figure 2-24: CD spectra of D-valinol-derived Macrocycle **7** in $CHCl_3$ and H_2O, recorded at 5 µM concentration.

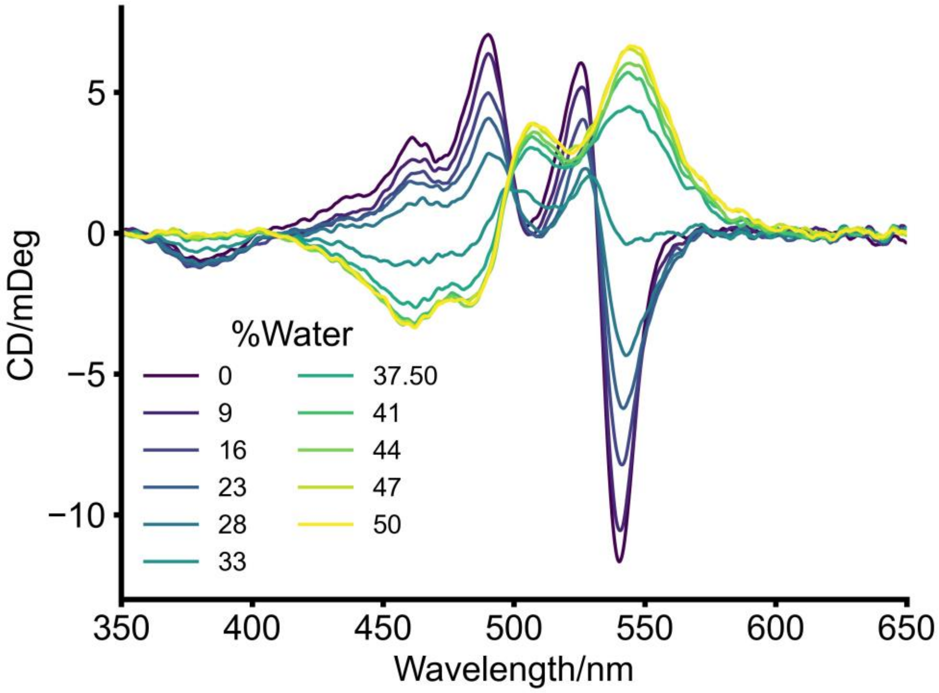

Supplementary Figure 2-25: Change in CD Spectrum upon addition of water into DMSO. Experiment was performed at 5 µM concentration of compound **5** in a 1 cm pathlength cuvette.

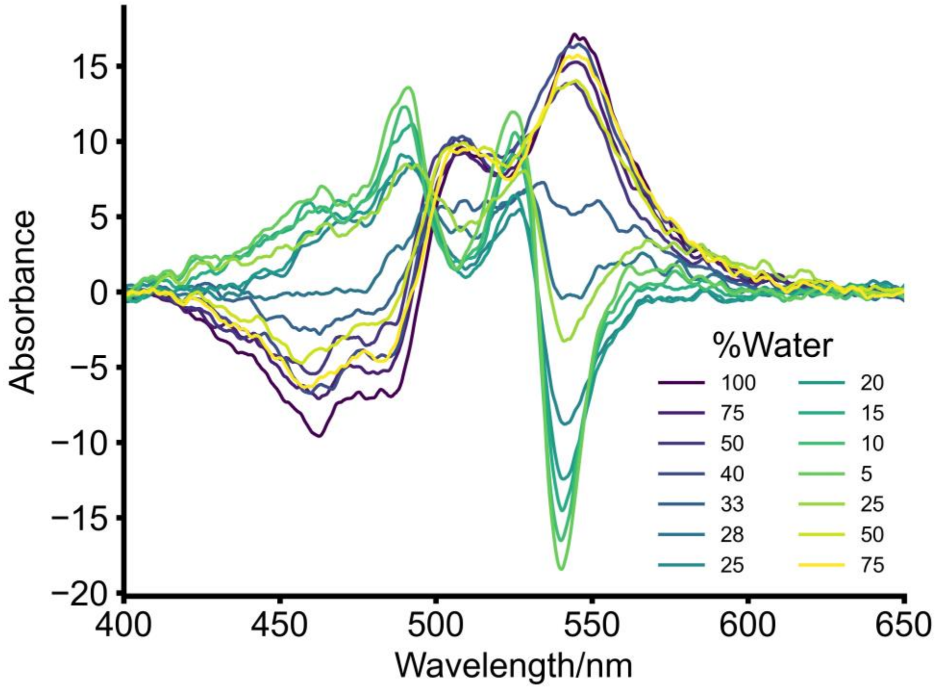

Supplementary Figure 2-26: Change in CD Spectrum upon addition of DMSO into water and back through addition of water. The inversion of CD signal is fully reversible. Experiment was performed at 10 µM concentration of compound **5** in a 1 cm pathlength cuvette.

3) CPL Measurements

General

CPL was measured with a home-built (modular) spectrometer. [3] The excitation source was a broad band (200 – 1000 nm) laser- driven light source EQ 99 (Elliot Scientific). The excitation wavelength was selected by feeding the broadband light into an Acton SP-2155 monochromator (Princeton Instruments, 7 µm slit); the collimated light was focused into the sample cell (1 cm quartz cuvette, temperature controlled at 20 ºC). Sample PL emission was collected perpendicular to the excitation direction with a lens (f = 150 mm). The emission was fed through a photoelastic modulator (PEM) (Hinds Series II/FS42AA) and through a linear sheet polariser (Comar). The light was then focused into a second scanning monochromator (Acton SP-2155, 7 µm slit) and subsequently on to a photomultiplier tube (PMT) (Hamamatsu H10723 series). The detection of the CPL signal was achieved using the field modulation lock-in technique. The electronic signal from the PMT was fed into a lock-in amplifier (Hinds Instruments Signaloc Model 2100). The reference signal for the lock-in detection was provided by the PEM control unit. The monchromators, PEM control unit and lock-in amplifier were interfaced to a desktop PC and controlled by a custom-written Labview graphic user interface. The lock-in amplifier provided two signals, an AC signal corresponding to (I_L- I_R) and a DC signal corresponding to (I_L + I_R) after background subtraction. The emission dissymmetry factor was therefore readily obtained from the experimental data, as 2 AC/DC.

Spectral calibration of the scanning monochromator was performed using a Hg-Ar calibration lamp (Ocean Optics, HAL-200). A correction factor for the wavelength dependence of the detection system was constructed using a calibrated lamp (Ocean Optics). The measured raw data was subsequently corrected using this correction factor. The validation of the CPL detection systems was achieved using light emitting diodes (LEDs) at various emission wavelengths. The LED was mounted in the sample holder and the light from the LED was fed through a broad band polarising filter and λ/4 plate (Ocean Optics) to generate circularly polarised light. Prior to all measurements, the λ/4 plate and a LED were used to set the phase of the lock-in amplifier correctly. The emission spectra were recorded with 2 nm step size and the slits of the detection monochromator were set to a slit width (vide supra) corresponding to a spectral resolution of 3.5 nm. CPL spectra (as well as total emission spectra) were obtained through an averaging procedure of several scans.

Measurements in $CHCl_3$ and PhMe were taken at 5 µM and the samples were excited at 450 nm. Spectra were recorded as an average of 5 measurements, except CPL titrations, which were single measurements. Measurements in H_2O were taken at 20 µM at 0.4% DMSO and excited at 493 nm. Spectra were recorded as an average of 20 measurements.

Calculated g_{lum}

Supplementary Table 3-1: Calculated g_{lum} values for Macrocycle **5** in various solvents. The experimental error of the instrument is ±5 x 10^{-5}. This error is larger than any of the fitting errors (± 6 x 10^{-6}, ± 2 x 10^{-5} and ± 6 x 10^{-6} for Index **1-3** respectively), which were calculated as standard errors of the mean for the fitted gaussian to the recorded CPL data (**Supplementary Figures 3-1** to **3-3**).

**Index**	**Solvent**	g_{lum}	**Wavelength/nm**
**1**	$CHCl_3$	−2 x 10^{-3}	542
**2**	H_2O	+1 x 10^{-3}	640
**3**	PhMe	−1 x 10^{-3}	543

Residuals of Gaussian fits to CPL data

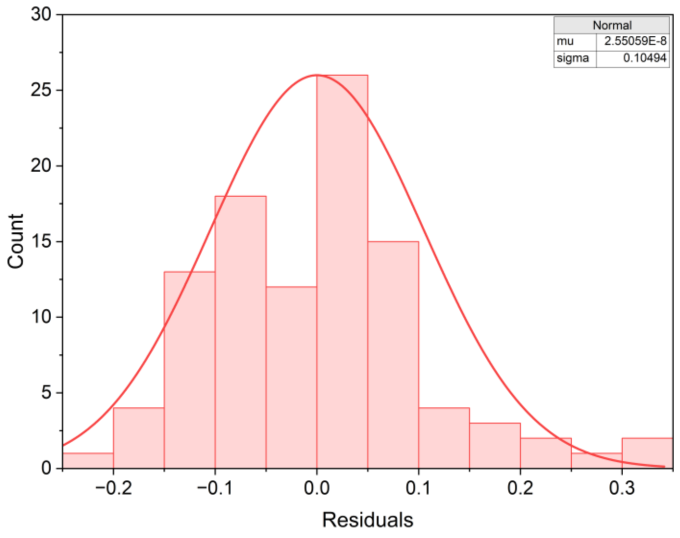

Supplementary Figure 3-1: Distribution of residuals of gaussian fit to CPL data in $CHCl_3$ (**Figure 4d**).

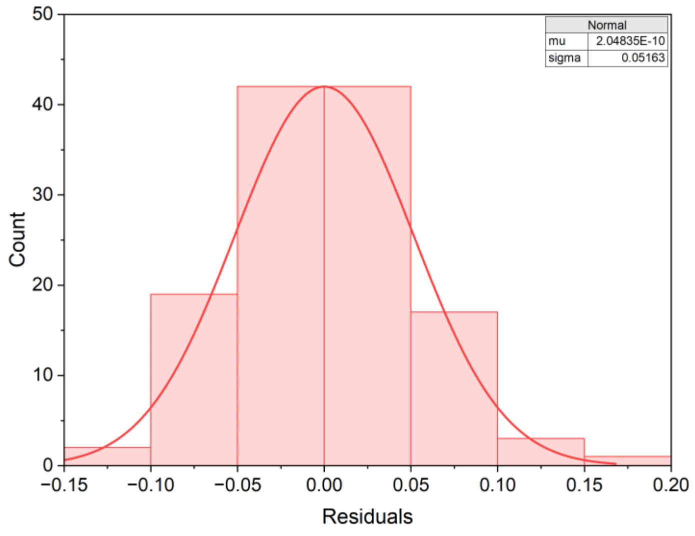

Supplementary Figure 3-2: Distribution of residuals of gaussian fit to CPL data in H_2O (**Figure 4e**).

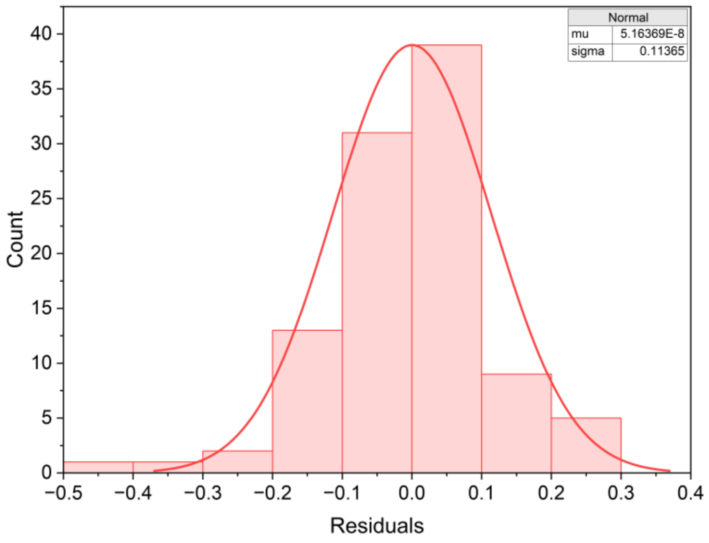

Supplementary Figure 3-3: Distribution of residuals of gaussian fit to CPL data in PhMe (**Supplementary Figure 4-37**).

4) Host/Guest Chemistry

General

Host/Guest Titrations were performed at ~5 µM concentration of macrocycle **5**, and ~10 µM of dimer **4**. Solutions of excess guest in a solution of host were added to a solution of pure host and absorbance spectra recorded on a Shimadzu UV-3600i Plus Spectrometer with 0.5 nm intervals at medium scan speed. The resulting data was then analysed via supramolecular.org using a 1:1 UV-visible absorbance binding model, fitting the charge transfer band region to ensure no interference coming from the absorbance of the guest species. Exact fitting parameters are given with each titration, as well as links to the saved data on supramolecular.org. Graphs show the increase in concentration of guest species going from purple (no guest) to yellow (maximum amount added).

Titration of Pyrene into Dimer in PhMe

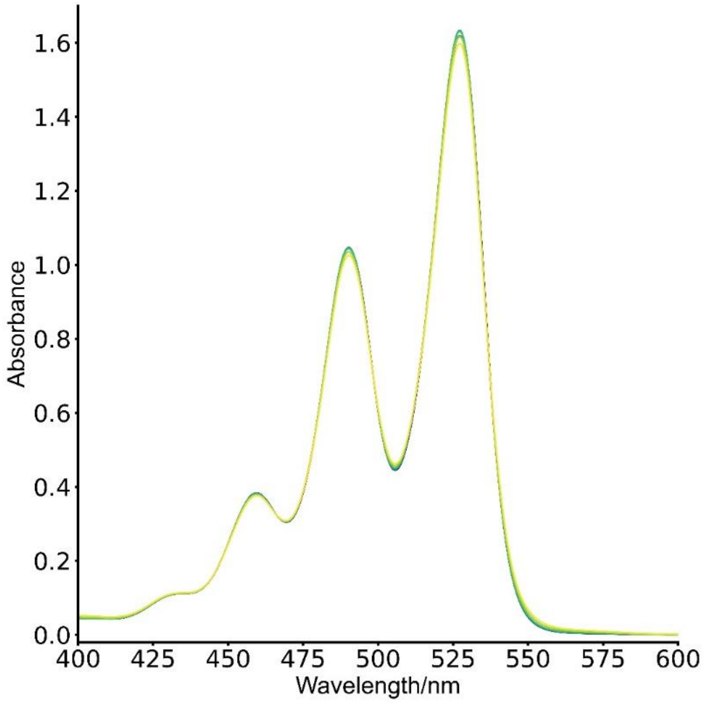

Supplementary Figure 4-1: Changes in Absorbance Spectra of Dimer **4** in PhMe at 10 µM upon addition of pyrene (0-5 mM). Fitting of the spectrum from 531 – 580 nm to a 1:1 binding model yielded a K_a of 48.00 M^{-1} with an error of ± 4.05%. Bindfit link: http://app.supramolecular.org/bindfit/view/37585cb0-3038-4232-9888-15f08e7dcce5

Titration of Pyrene into Dimer in $CHCl_3$

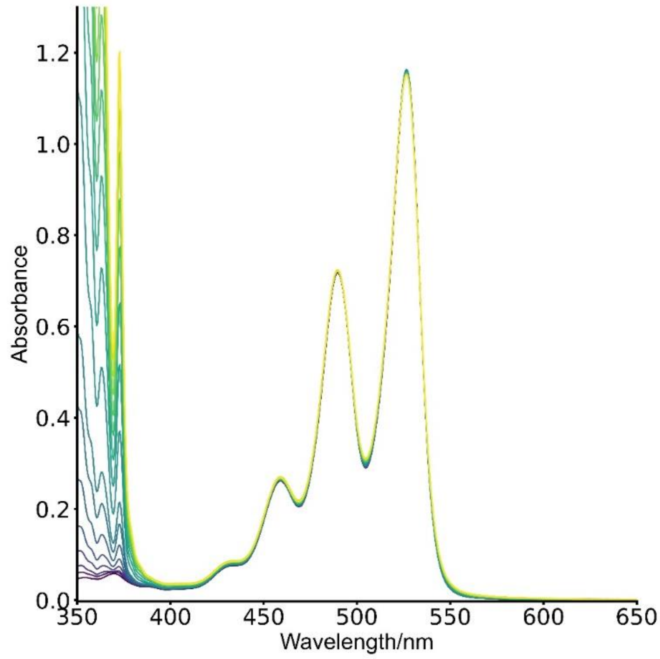

Supplementary Figure 4-2: Changes in Absorbance Spectra of Dimer **4** in $CHCl_3$ at 9.8 µM upon addition of pyrene (0-4.8 mM). The binding was too weak to be accurately fitted.

Titration of Pyrene into Macrocycle 5 in PhMe

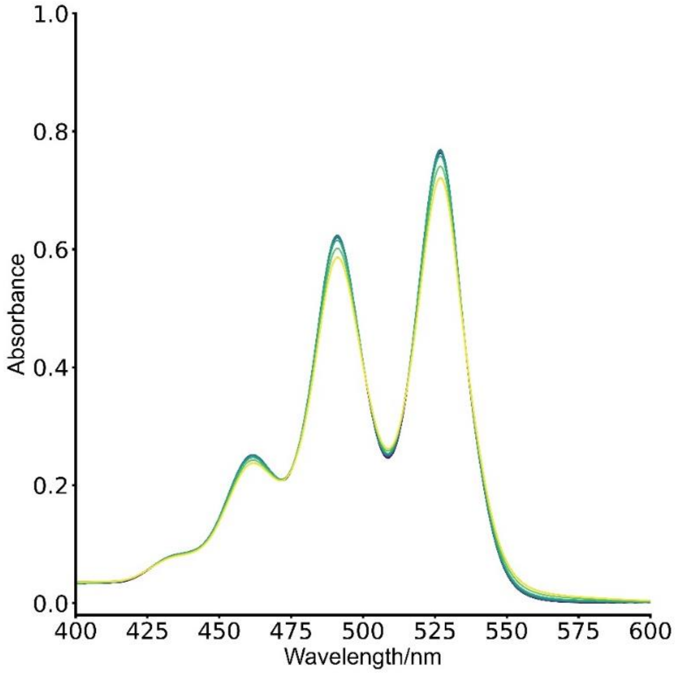

Supplementary Figure 4-3: Changes in absorbance spectra of macrocycle 5 in PhMe at 5 µM upon addition of pyrene (0-5 mM). Fitting of the spectrum from 553-570 nm to a 1:1 binding model yielded a K_a of 111.11 M^{-1} with an error of ± 0.95%. Bindfit link: http://app.supramolecular.org/bindfit/view/94580575-6148-4d63-9b3e-2b66c6f9e91e

Titration of Pyrene into Macrocycle 5 in $CHCl_3$

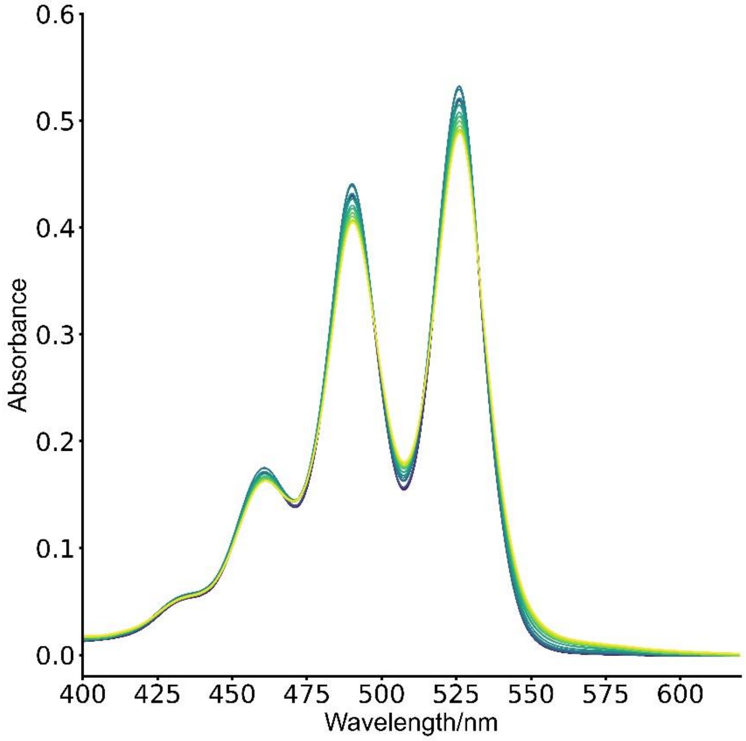

Supplementary Figure 4-4: Changes in absorbance spectra of macrocycle 5 in $CHCl_3$ at 4.7 µM upon addition of pyrene (0-4.8 mM). Fitting of the spectrum from 531-571 nm to a 1:1 binding model yielded a K_a of 53.69 M^{-1} with an error of ± 0.42%. Bindfit link: http://app.supramolecular.org/bindfit/view/278c0d96-6cbe-4aee-988b-dd19d7117ca6

Titration of Corannulene into Macrocycle 5 in PhMe

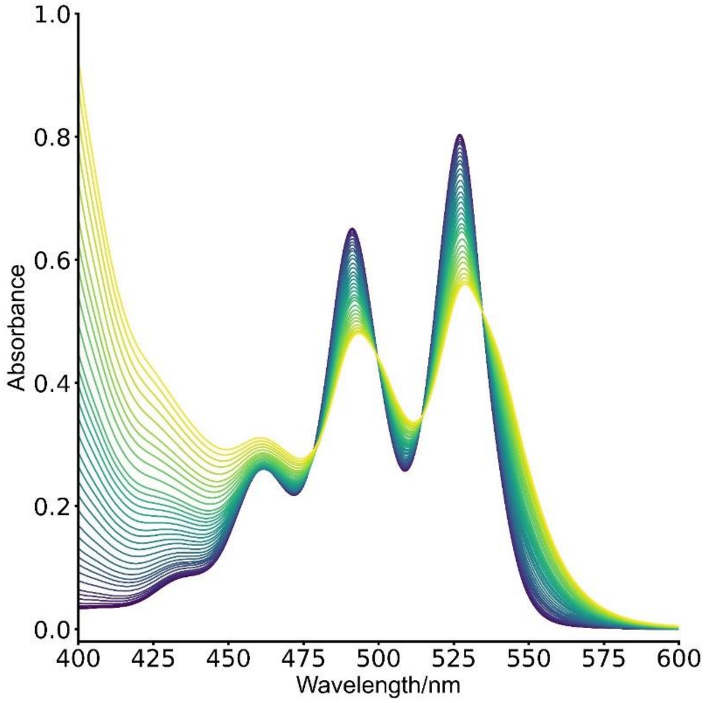

Supplementary Figure 4-5: Changes in absorbance spectra of macrocycle 5 in PhMe at 5.22 µM upon addition of corannulene (0-4.9 mM). Fitting of the spectrum from 531-650 nm to a 1:1 binding model yielded a K_a of 193.68 M^{-1} with an error of ± 0.08%. Bindfit link: http://app.supramolecular.org/bindfit/view/620ae0d1-b30c-443b-b9e1-10934b6962cf

Titration of Corannulene into Macrocycle 5 in $CHCl_3$

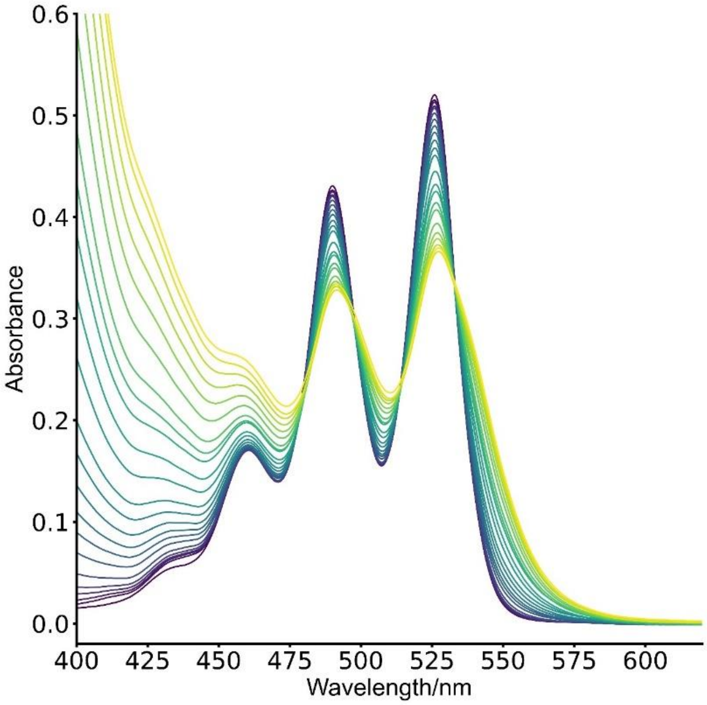

Supplementary Figure 4-6: Changes in absorbance spectra of macrocycle 5 in $CHCl_3$ at ~4.41 µM upon addition of corannulene (0-4.76 mM). Fitting of the spectrum from 521-585 nm to a 1:1 binding model yielded a K_a of 193.87 M^{-1} with an error of ± 0.29%. Bindfit link: http://app.supramolecular.org/bindfit/view/3bab07db-45c0-401b-8850-1a84ce66f513.

Titration of Perylene into Macrocycle 5 in PhMe

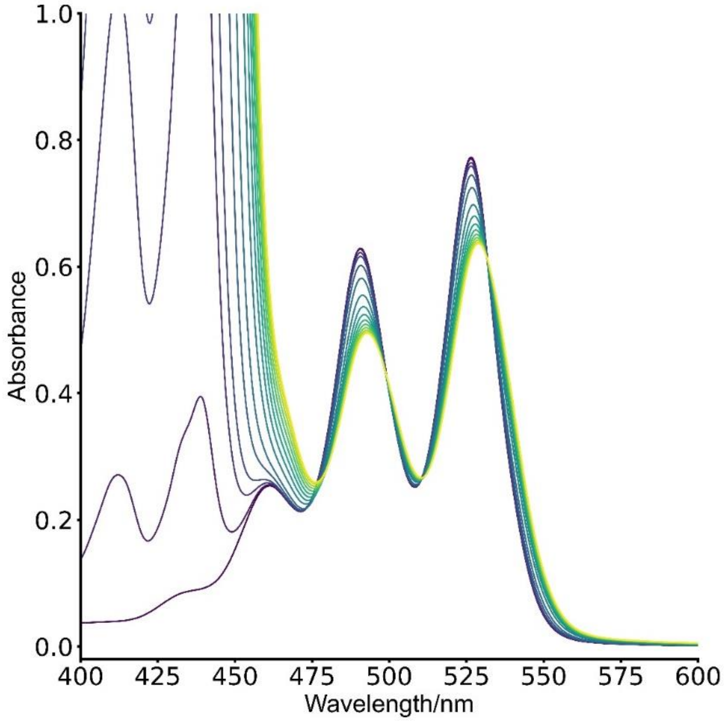

Supplementary Figure 4-7: Changes in absorbance spectra of macrocycle 5 in PhMe at 5 µM upon addition of perylene (0-2.5 mM). Fitting of the spectrum from 537-571 nm to a 1:1 binding model yielded a K_a of 540.61 M^{-1} with an error of ± 0.13%. Bindfit link: http://app.supramolecular.org/bindfit/view/efa69c9e-457e-4ba4-904a-4d584552a249

Titration of Perylene into Macrocycle 5 in $CHCl_3$

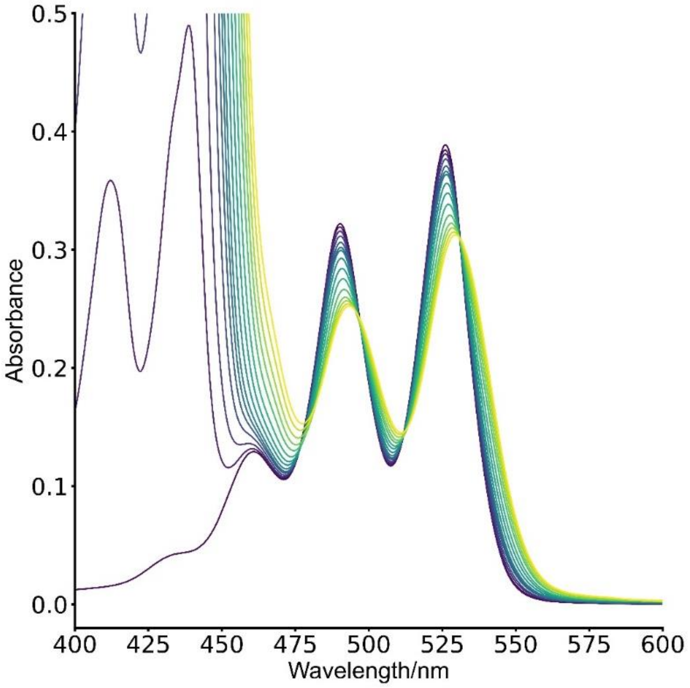

Supplementary Figure 4-8: Changes in absorbance spectra of macrocycle 5 in $CHCl_3$ at 3.75 µM upon addition of perylene (0-1.67 mM). Fitting of the spectrum from 521-543 nm to a 1:1 binding model yielded a K_a of 1062.74 M^{-1} with an error of ± 0.53. Bindfit link: http://app.supramolecular.org/bindfit/view/5c058aa6-7c23-4134-9b16-1e6faf992365

Titration of Coronene into Macrocycle 5 in PhMe

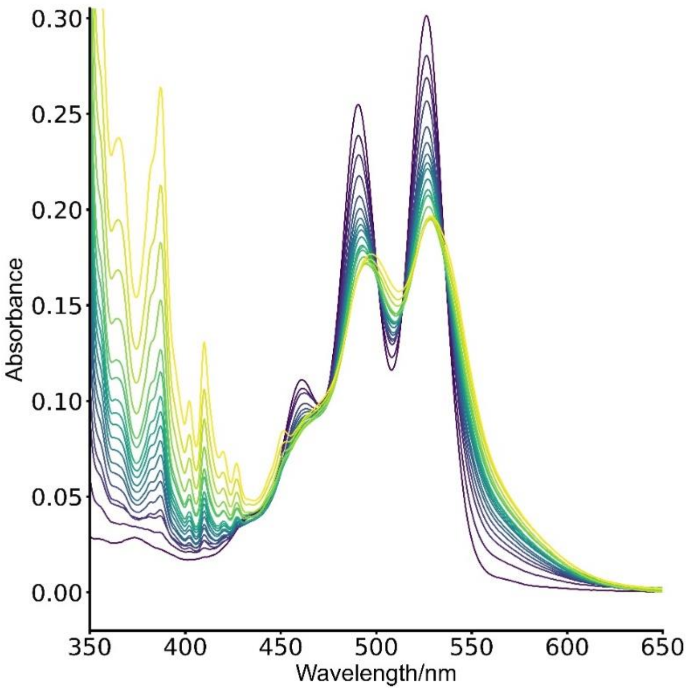

Supplementary Figure 4-9: Changes in absorbance spectra of macrocycle 5 in PhMe at 1.85 µM upon addition of coronene (0-0.13 mM). Fitting of the spectrum from 531-571 nm to a 1:1 binding model yielded a K_a of 29181.26 M^{-1} with an error of ± 0.40%. Bindfit link: http://app.supramolecular.org/bindfit/view/90179981-b7ab-4197-aca4-9fc341dd99da

Titration of Coronene into Macrocycle 5 in $CHCl_3$

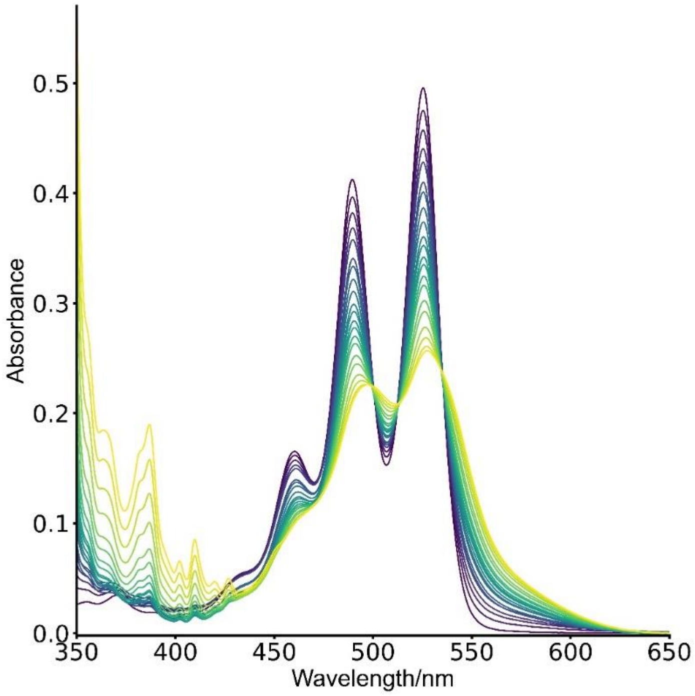

Supplementary Figure 4-10: Changes in absorbance spectra of macrocycle 5 in $CHCl_3$ at ~5 µM upon addition of perylene (0-0.26 mM). Fitting of the spectrum from 537-595 nm to a 1:1 binding model yielded a K_a of 37741.05 M^{-1} with an error of ± 0.3896%. Bindfit link: http://app.supramolecular.org/bindfit/view/12ce6f2d-477f-49df-8d99-f0feff1932a8

ITC of Macrocycle and Coronene

ITC spectra were recorded on a Malvern Panalytical MicroCal PEAQ-ITC, using a reference power of 10 µcal/s and a stirring speed of 750 RPM. 19 injections were performed with 150 seconds delay between injections. Data was recorded at 25 °C. Concentration of the coronene guest species inside the syringe was 1 mM, and the concentration of macrocycle inside the cell was 100 µM. The first 2 injections were discarded. The data was then baseline-corrected and fitted using the built-in software, with the number of sites set to 1, as the number of sites = 1 was obvious from the turning point at 1 molar equivalent. The experiment was performed in triplicate.

ITC Thermogram

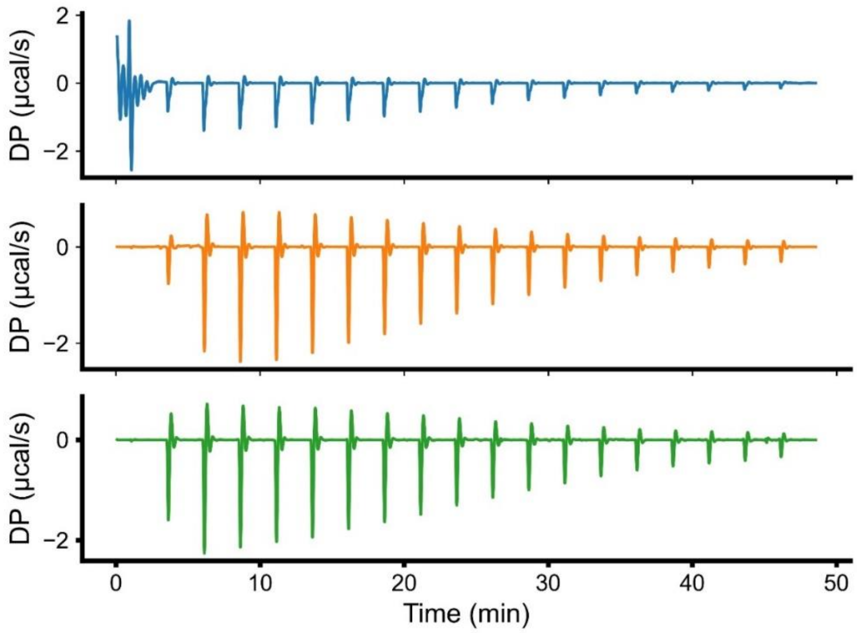

Supplementary Figure 4-11: Baseline-corrected ITC traces of macrocycle **5** + Coronene in $CHCl_3$

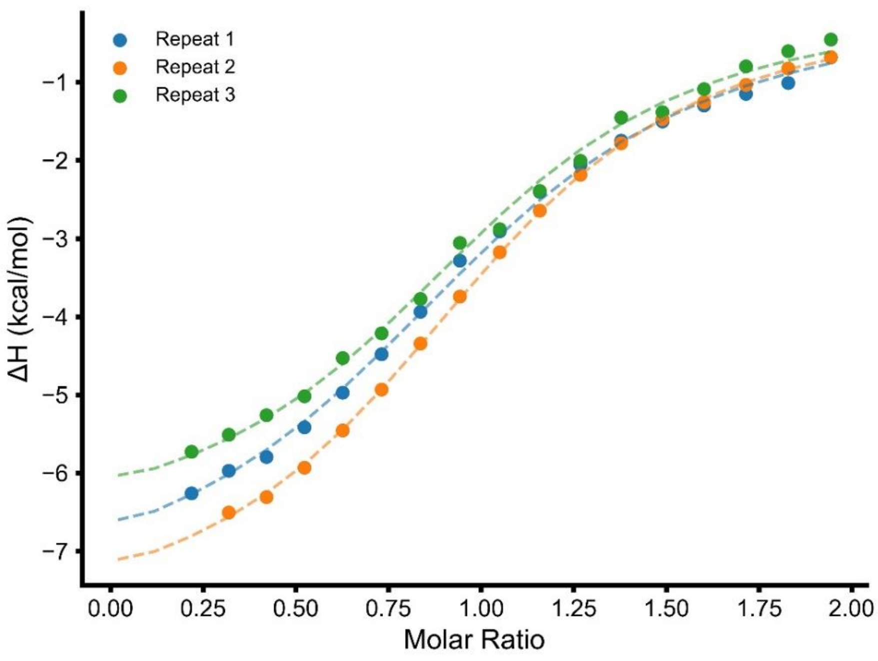

Supplementary Figure 4-12: Integrated heat plot of Coronene and macrocycle **5**. Dots represent recorded data and dashed line represent the fitted data.

Index	***Temperature (°C)***	***K_D (M)***	***K_D Error (M)***	***ΔH (kcal/mol)***	***ΔH Error (kcal/mol)***	***ΔG (kcal/mol)***	***-TΔS (kcal/mol)***
1	*25.1*	*1.57 x 10^{-5}*	*1.96 x 10^{-6}*	*-7.65*	*0.33*	*-6.56*	*1.1*
2	*25.3*	*1.23 x 10^{-5}*	*6.18 x 10^{-7}*	*-8*	*0.135*	*-6.71*	*1.29*
3	*25.2*	*1.27 x 10^{-5}*	*2.11 x 10^{-6}*	*-6.81*	*0.361*	*-6.68*	*0.126*
Avg	*25.2*	*1.35 x 10^{-5}*	*3.42 x 10^{-6}*	*-7.49*	*0.275*	*-6.65*	*0.839*

Supplementary Table 4-1: Thermodynamic parameters calculated from ITC data for coronene as titrant. The measured K_d corresponds to a K_a of 7.4 x 10^4 M^{-1}. From the thermodynamic parameters resulting from the experiment, we can conclude that binding of a guest species inside the macrocycle cavity is driven by enthalpy.

Fluorescence Titrations with Coronene as Guest

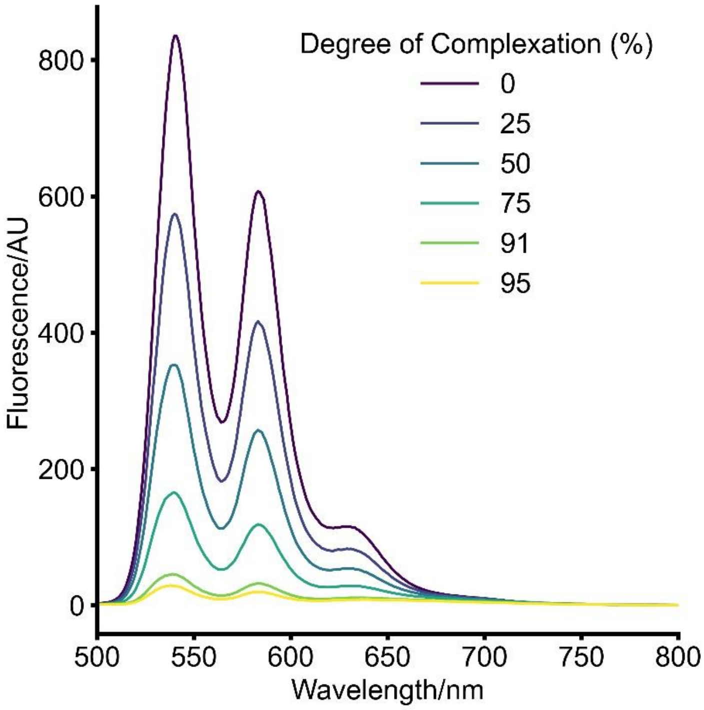

Supplementary Figure 4-13: Fluorescence of Macrocycle **5** at 5 µM in $CHCl_3$ excited at 490 nm upon addition of coronene, with the PMT set at 530 V. The degree of complexation is known from the K_a value for coronene.

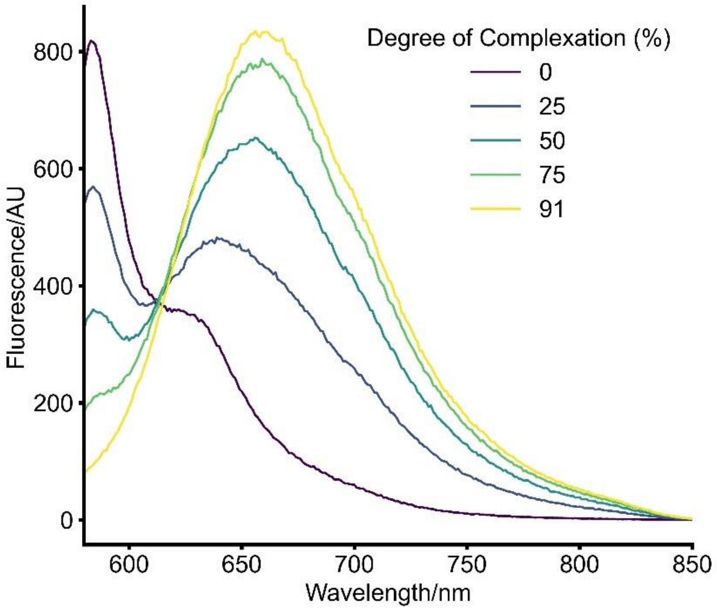

Supplementary Figure 4-14: Fluorescence of Macrocycle **5** at 5 µM in $CHCl_3$ excited at 570 nm (the charge transfer band) upon addition of Coronene, with the PMT set at 800 V. a clear reduction in fluorescence intensity is observed for the macrocycle band, and a clear exciplex emission band is observed, increasing with increasing %HG in solution. The data was fitted to a 1:1 binding model, yielding a K_a of 75891.56 M^{-1} with an error of ± 0.8785%. Bindfit link: http://app.supramolecular.org/bindfit/view/f9504792-696c-4382-ae80-d339c42c12e2. The degree of complexation is known from the K_a value for coronene.

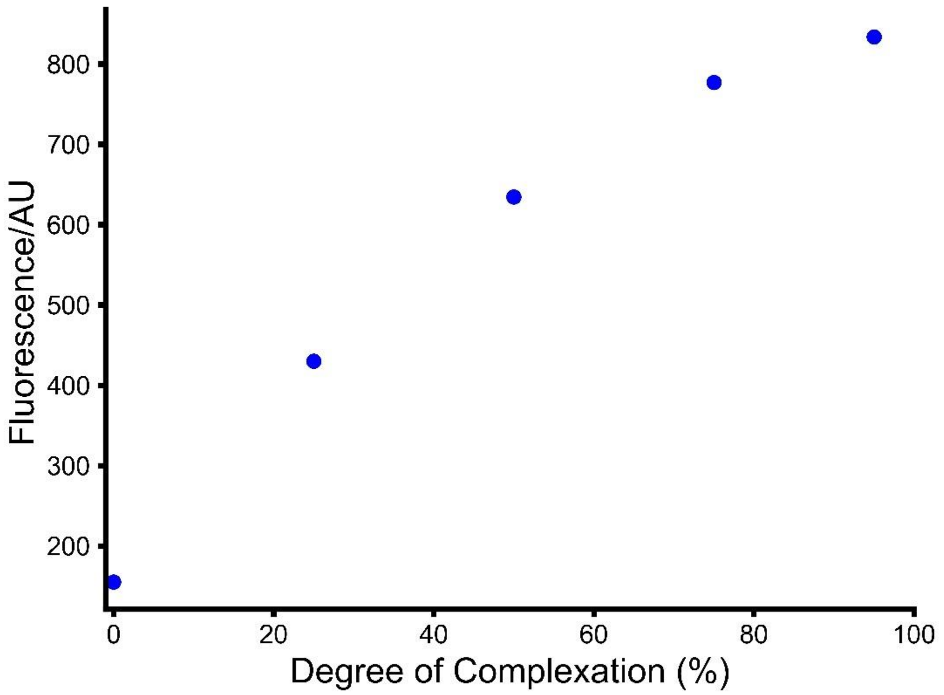

Supplementary Figure 4-15: Plot of fluorescence intensity of the exciplex band (630 nm) against %HG complex in solution. The degree of complexation is known from the K_a value for coronene.

Fluorescence Titrations with Perylene as Guest

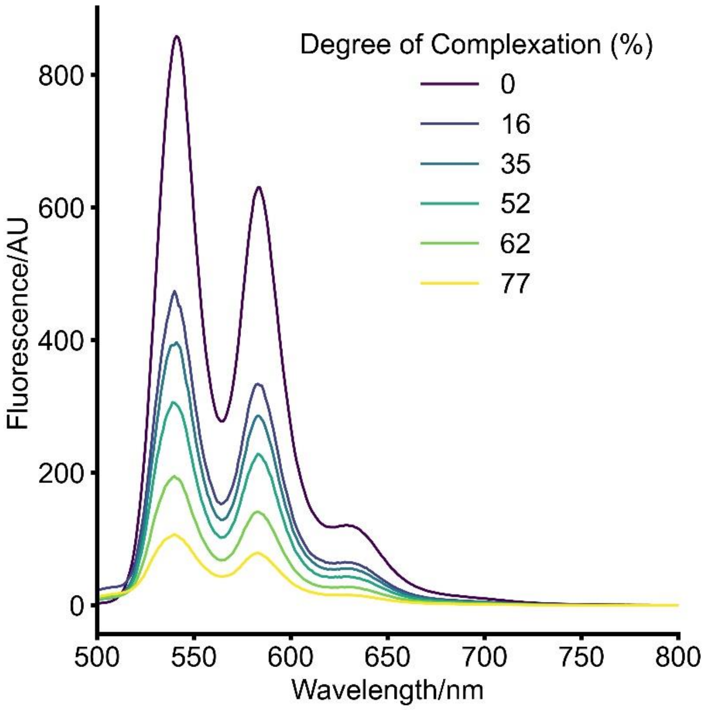

Supplementary Figure 4-16: Fluorescence of Macrocycle **5** at 5 µM in $CHCl_3$ excited at 490 nm upon addition of Perylene, with the PMT set at 530 V. The degree of complexation is known from the K_a value for perylene.

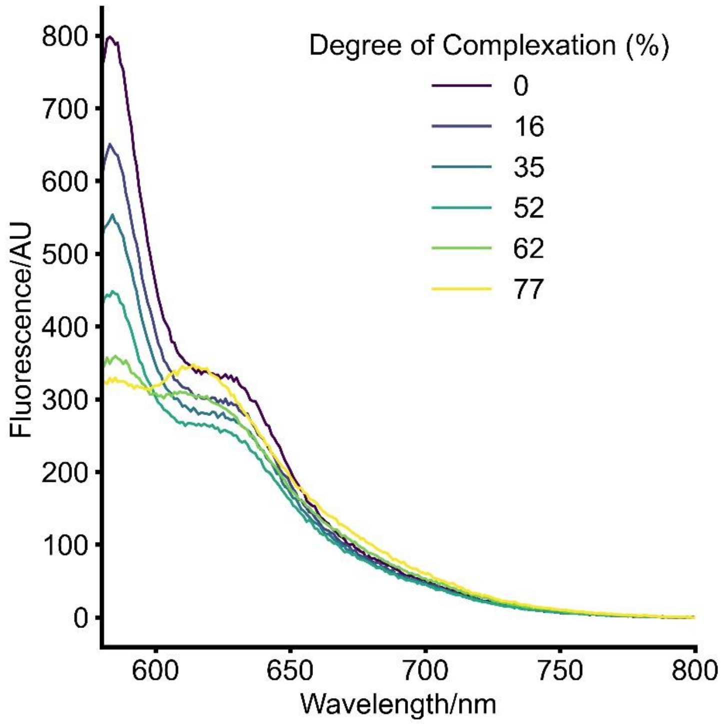

Supplementary Figure 4-17: Fluorescence of Macrocycle **5** at 5 µM in $CHCl_3$ excited at 570 nm (the charge transfer band) upon addition of Perylene, with the PMT set at 800 V. A clear reduction in fluorescence intensity is observed for the PDI monomeric emission, and exciplex emission is observed emerging at ~625 nm. The degree of complexation is known from the K_a value for perylene.

Circular Dichroism Titrations with Coronene as Guest

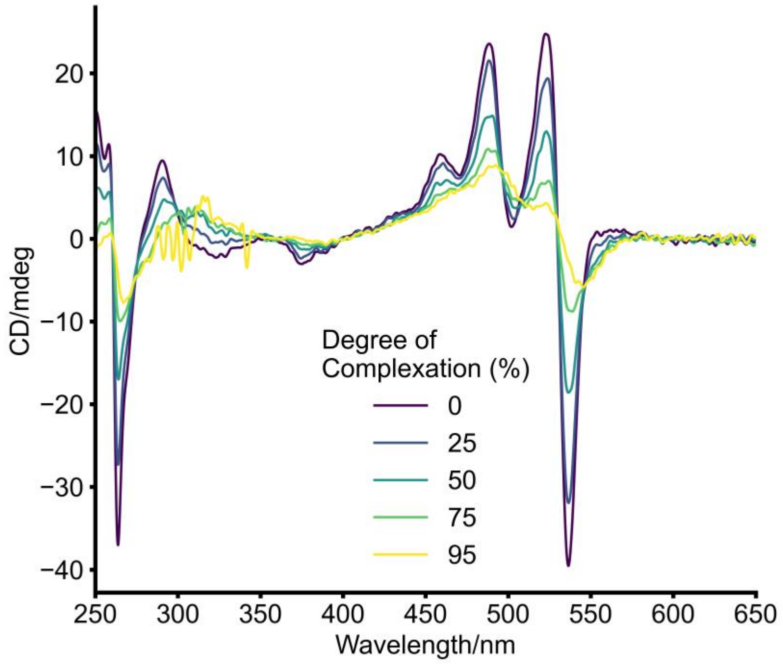

Supplementary Figure 4-18: Change in Circular Dichroism of Macrocycle **5** (100 µM in $CHCl_3$) upon addition of coronene. A clear decrease in CD intensity is observed. Notable is that there is no emerging CD signal at ~300 nm, the absorbing region of coronene. Fitting of the data (400-600 nm) to a 1:1 binding model yielded a K_a of 46730.86 M^{-1} with an error of ±1.4525%. Bindfit link: http://app.supramolecular.org/bindfit/view/435c3f9d-2089-401b-90b7-1bd2bfbadb4c. The degree of complexation is known from the K_a value for coronene.

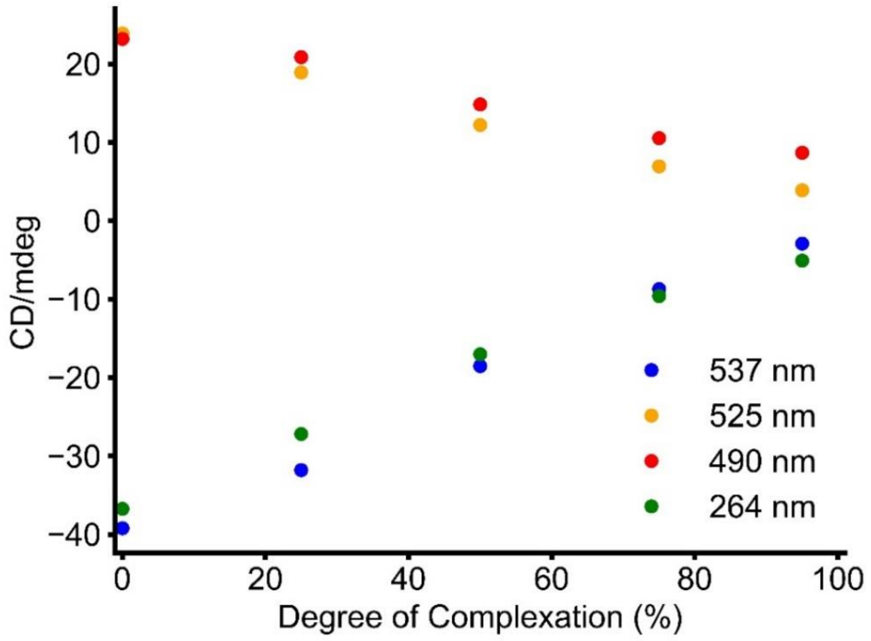

Supplementary Figure 4-19: Changes in CD maxima from **Supplementary Figure 4-18** with regards to the degree of complexation (%). A clear CD reduction is observed for all wavelengths.

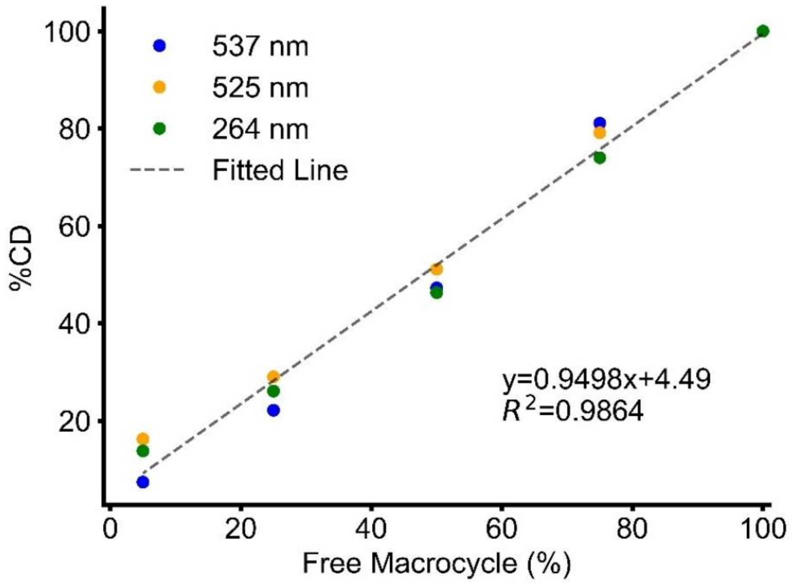

Supplementary Figure 4-20: Free macrocycle (in %) vs %CD plot from the data in **Supplementary Figure 4-18**. A trendline fitted to the data yields a slope of close to 1 and an intercept of 4.49, which gives an estimate of the relative CD intensity arising from the host-guest complex (4%).

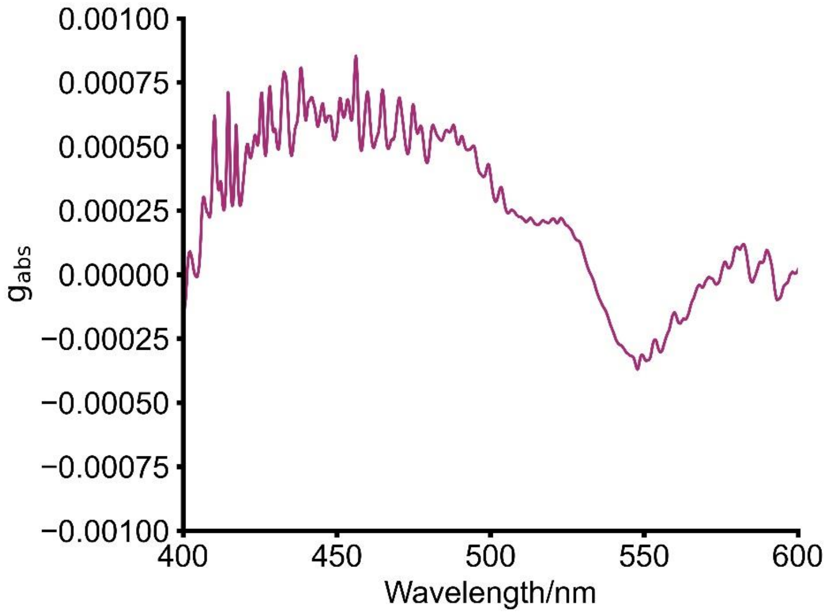

Supplementary Figure 4-21: Calculated g_{abs} from **Supplementary Figure 4-18** for 95% complexation in $CHCl_3$. The g_{abs} value at 550 nm is $-4 \times 10^{-4} \pm 9 \times 10^{-6}$.

Circular Dichroism Titrations with Perylene as Guest

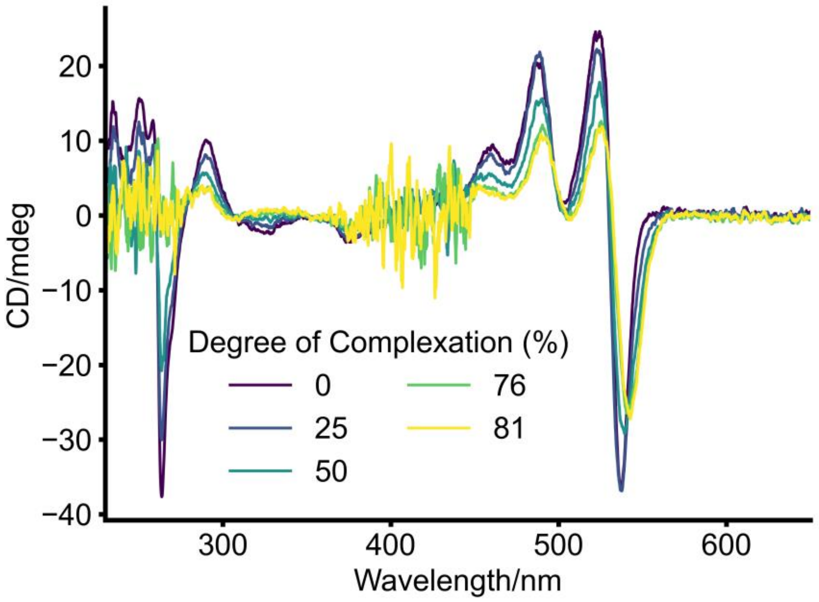

Supplementary Figure 4-22: Change in Circular Dichroism of Macrocycle **5** (100 µM in $CHCl_3$) upon addition of perylene. A decrease in CD intensity is observed. Notable is that there is no emerging CD signal at ~400-450 nm, the absorbing region of perylene. The degree of complexation is known from the K_a value for perylene.

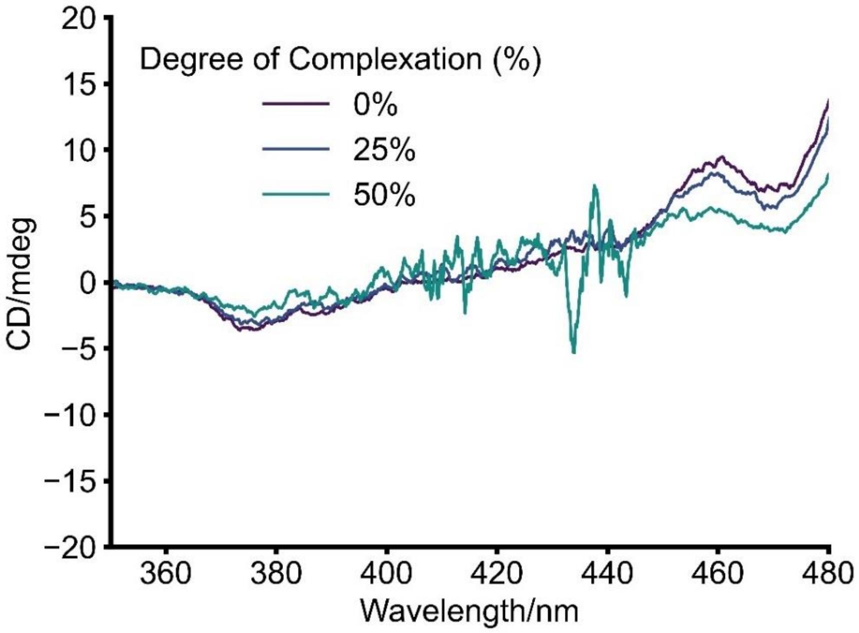

Supplementary Figure 4-23: Zoom into the absorbing region of perylene in **Supplementary Figure 4-22.** No CD signal from the bound guest is observed.

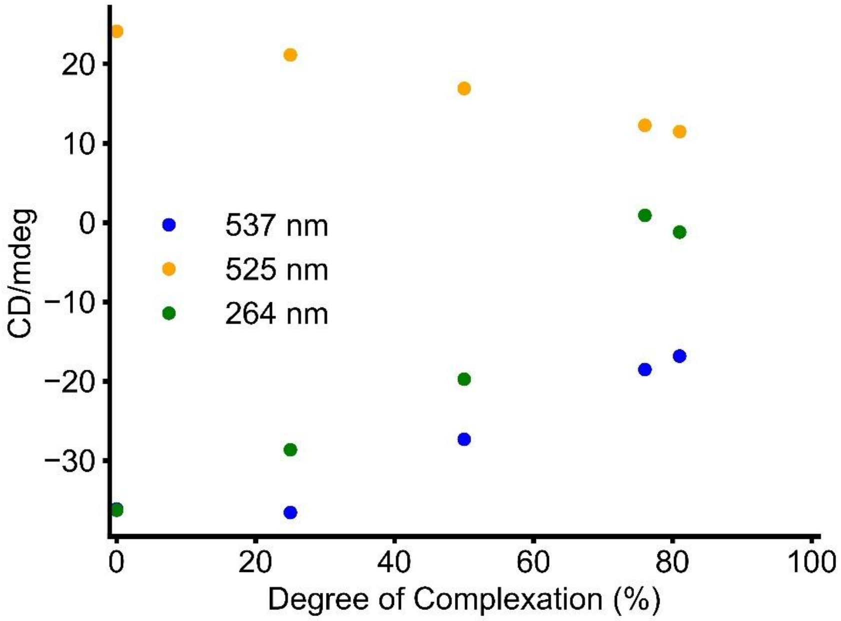

Supplementary Figure 4-24: Changes in CD maxima from **Supplementary Figure 4-22** with regards to the degree of complexation (%). A loss of CD signal intensity is observed for all wavelengths.

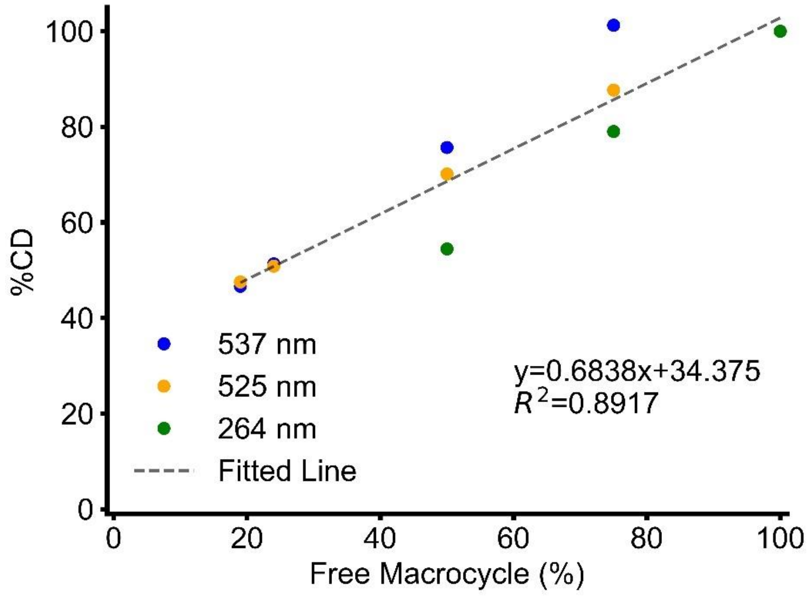

Supplementary Figure 4-25: % free macrocycle vs %CD (425 nm) plot from the data in **Supplementary Figure 4-22**, showing a linear relationship between residual host and CD signal. A trendline fitted to the data yields an intercept of 34.4, which gives an estimate of the relative CD intensity arising from the host-guest complex (34%). Datapoints for 264 nm at 19% and 24% were discarded due to noise in the data.

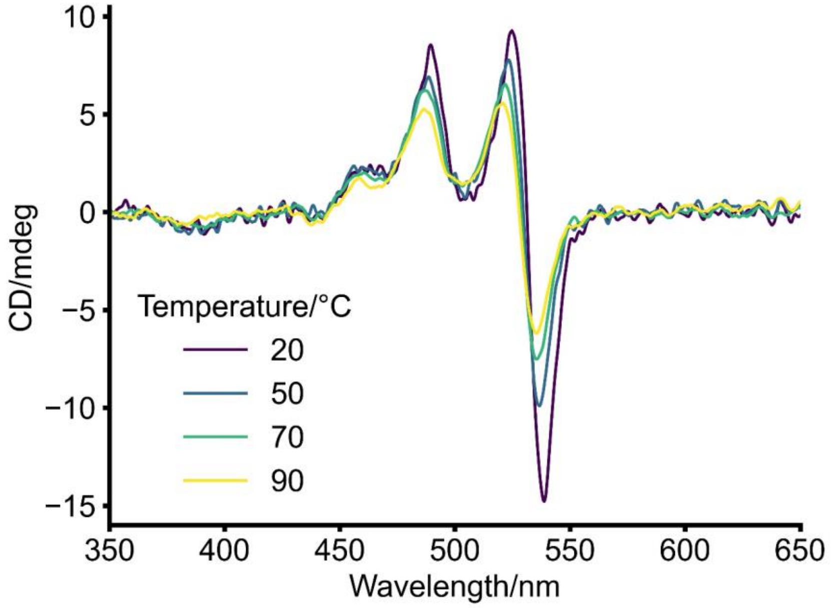

Supplementary Figure 4-26: Change in CD intensity of a solution of macrocycle **5** at 5 µM in PhMe at different temperatures. There is a decrease of CD signal at higher temperatures.

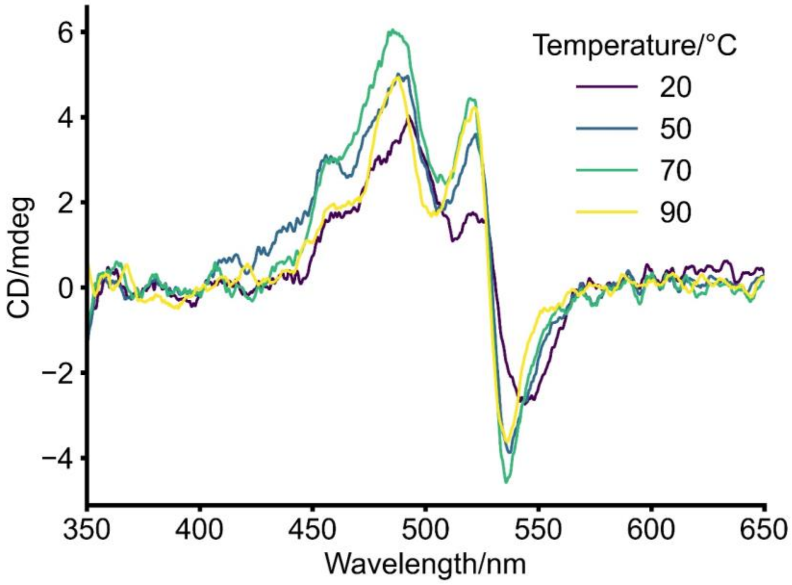

Supplementary Figure 4-27: Change in CD intensity of a solution of macrocycle **5** at 90% complexation with coronene at 5 µM in PhMe at different temperatures. Upon increasing temperature, decomplexation occurs, increasing the CD intensity, countering the effects of CD loss observed with pure macrocycle in **Supplementary Figure 4-26**.

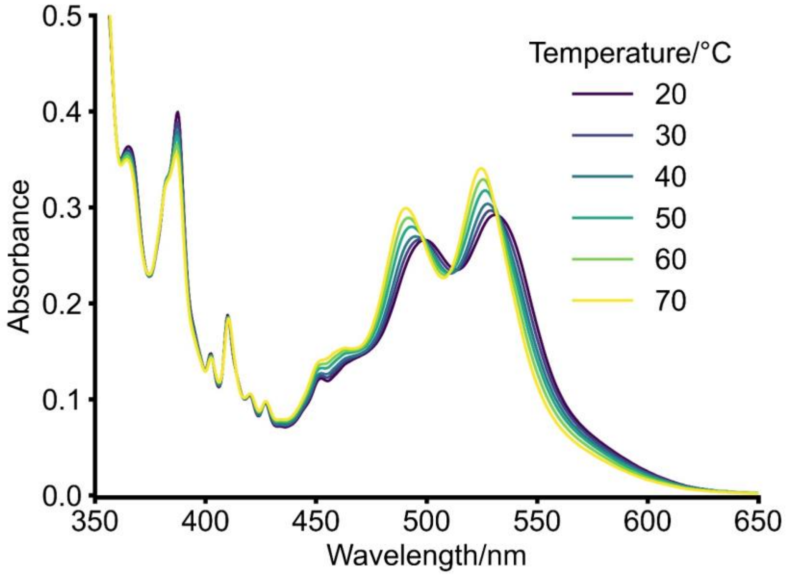

Supplementary Figure 4-28: Change in absorbance spectrum of a solution of macrocycle **5** at 90% complexation with coronene at 5 µM in PhMe at different temperatures. Upon increasing temperature, decomplexation occurs.

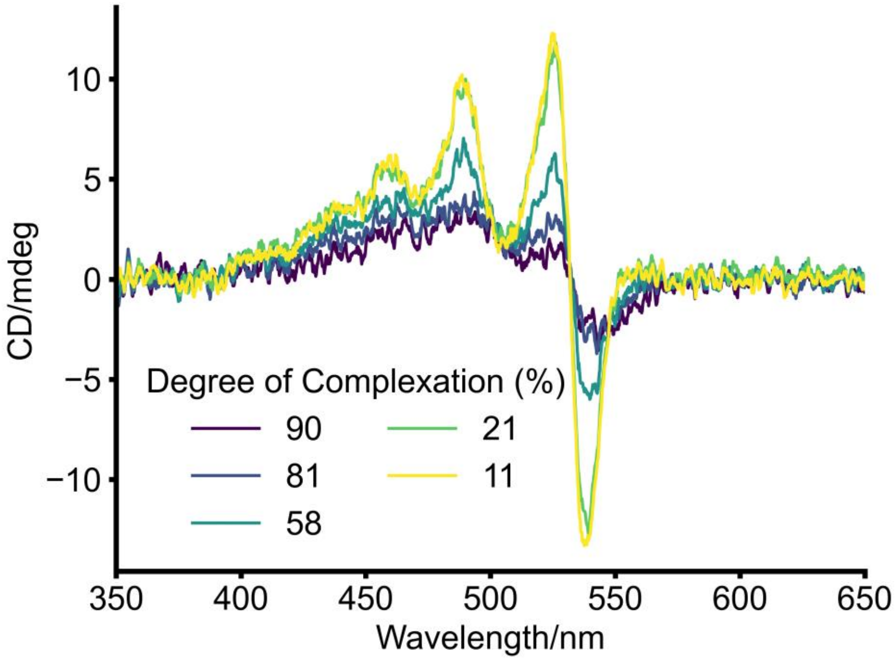

Supplementary Figure 4-29: Increase in CD signal of a solution of macrocycle **5** at 5 µM with 60 eq. coronene (90% complexation) in PhMe upon dilution with a solution of pure host down to 1 eq. coronene (11% complexation).

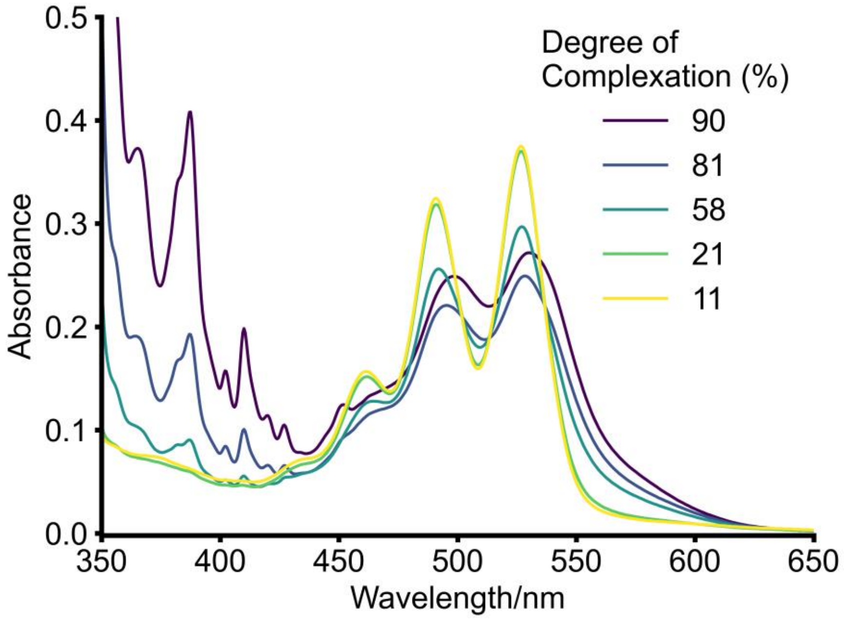

Supplementary Figure 4-30: Changes in absorbance of a solution of macrocycle **5** at 5 µM with 60 eq. coronene (90% complexation) in PhMe upon dilution with a solution of pure host down to 1 eq. coronene (11% complexation).

CPL Host/Guest Titrations with Coronene

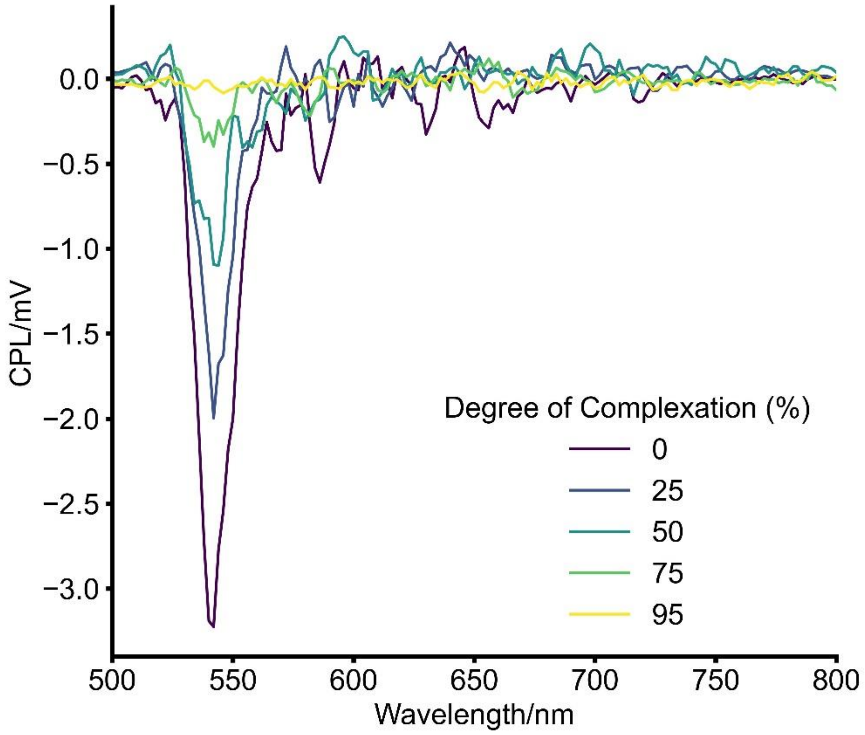

Supplementary Figure 4-31: CPL spectra of macrocycle **5** at 5 µM in $CHCl_3$ excited at 490 nm with varying amounts of coronene, measured at 900 gain and smoothed using moving average. A clear reduction in CPL is observed upon complex formation. The degree of complexation is known from the K_a value for coronene.

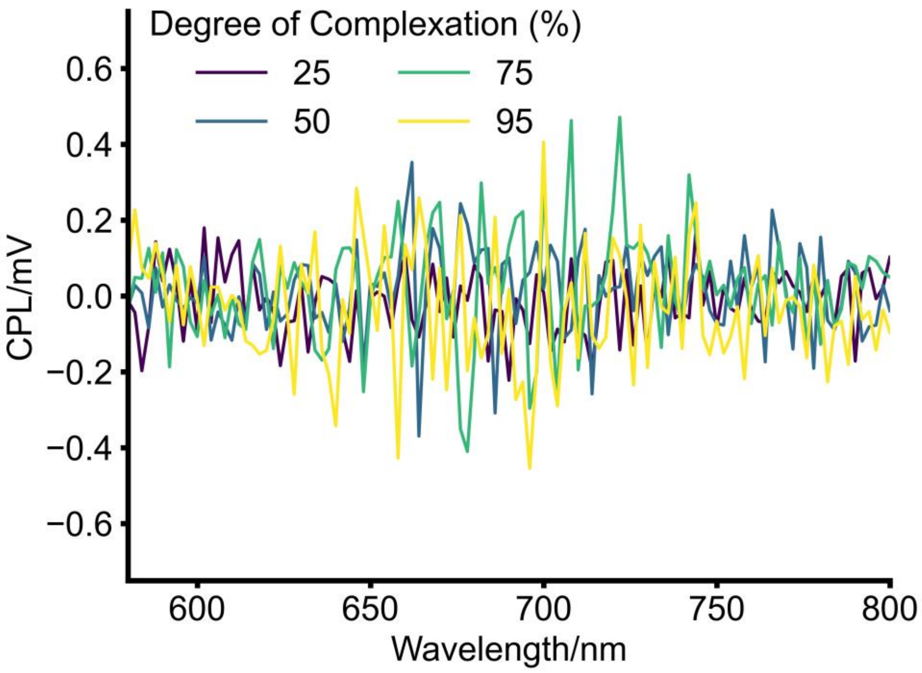

Supplementary Figure 4-32: CPL spectra of macrocycle **5** at 5 µM in $CHCl_3$ excited at 570 nm (charge-transfer band) with varying amounts of coronene, measured at max (1111) gain. The degree of complexation is known from the K_a value for coronene.

CPL Host/Guest Titrations with Perylene

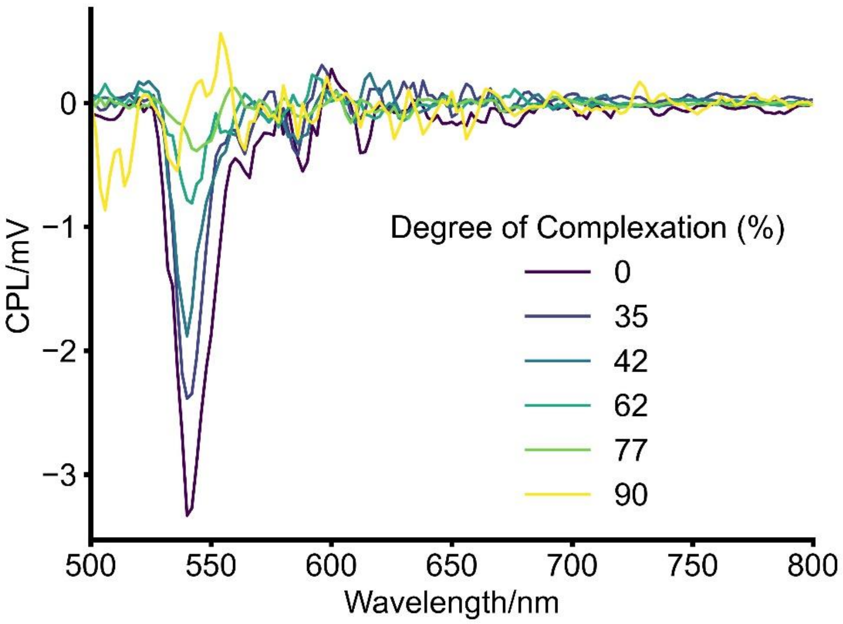

Supplementary Figure 4-33: CPL spectra of macrocycle 5 at 5 µM in $CHCl_3$ excited at 490 nm with varying amounts of perylene, measured at 900 gain and smoothed using moving average. A clear reduction in CPL is observed upon complex formation. The degree of complexation is known from the K_a value for perylene.

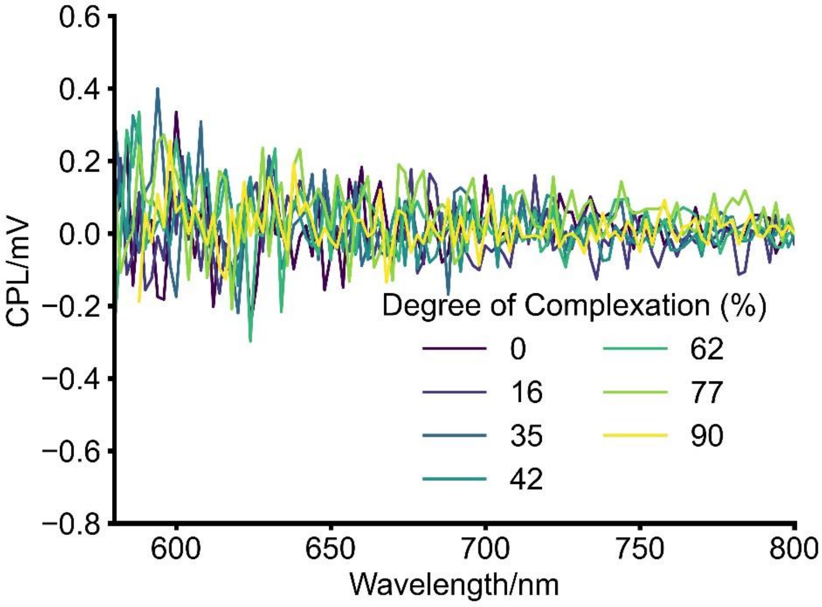

Supplementary Figure 4-34: CPL spectra of macrocycle **5** at 5 µM in $CHCl_3$ excited at 570 nm (charge transfer band) with varying amounts of perylene, measured at max (1111) gain. The degree of complexation is known from the K_a value for perylene.

Circular Dichroism and Absorbance of Macrocycle 5 in PhMe saturated with Coronene

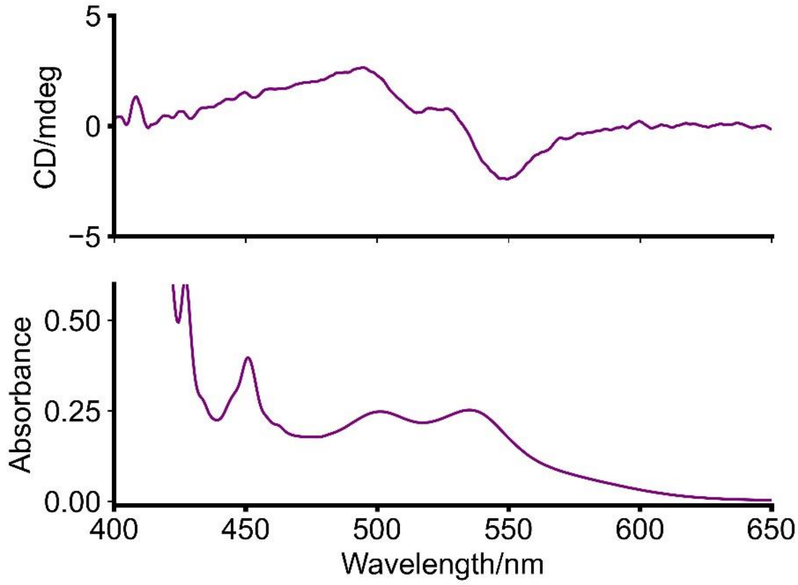

Supplementary Figure 4-35: CD and Absorbance Spectra of Macrocycle **5** at 5 µM with coronene at saturation (~98% Host/Guest Complex) in PhMe at 1 cm pathlength. The signal intensity of the CD is drastically weakened compared to the free macrocycle, as seen with $CHCl_3$. The g_{abs} at 554 nm is -5×10^{-4}.

Fluorescence Spectra of 5–Coronene at Different Excitation Wavelengths

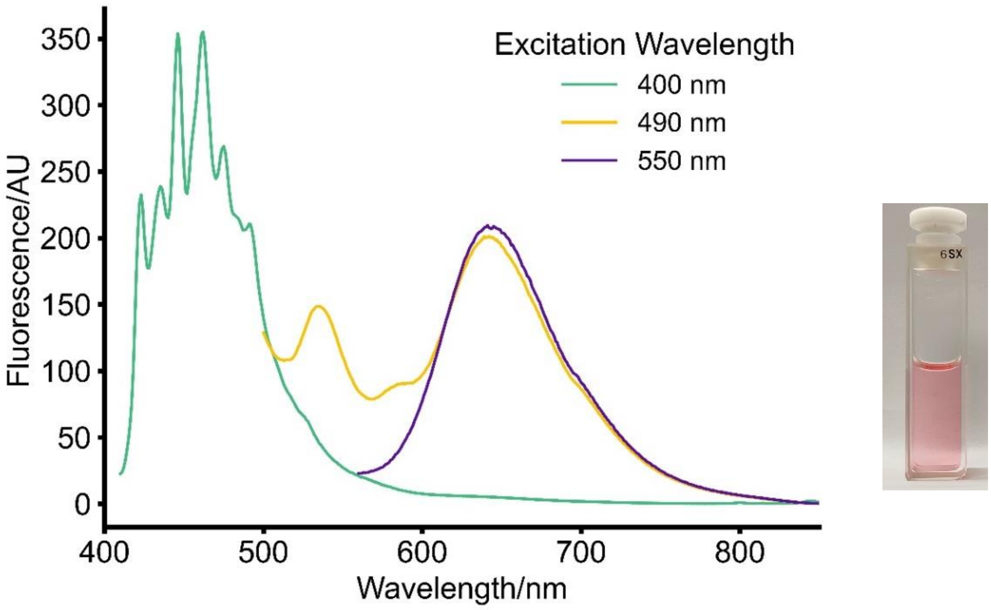

Supplementary Figure 4-36: Emission spectra of the **5**–coronene host–guest complex (~98% degree of complexation) in PhMe at different excitation wavelengths. The emission between 400-500nm arises from excitation of the coronene guest (λ_{ex} = 400 nm), the peak at 530 nm is the PDI macrocycle "monomer", which is weak and observed upon excitation of the PDI (λ_{ex} = 490 nm). The peak at 650nm is the exciplex emission, seen when excited at the charge transfer band (λ_{ex} = 550 nm). The photograph shows the colour of the complex solution. The degree of complexation is known from the K_a value for coronene.

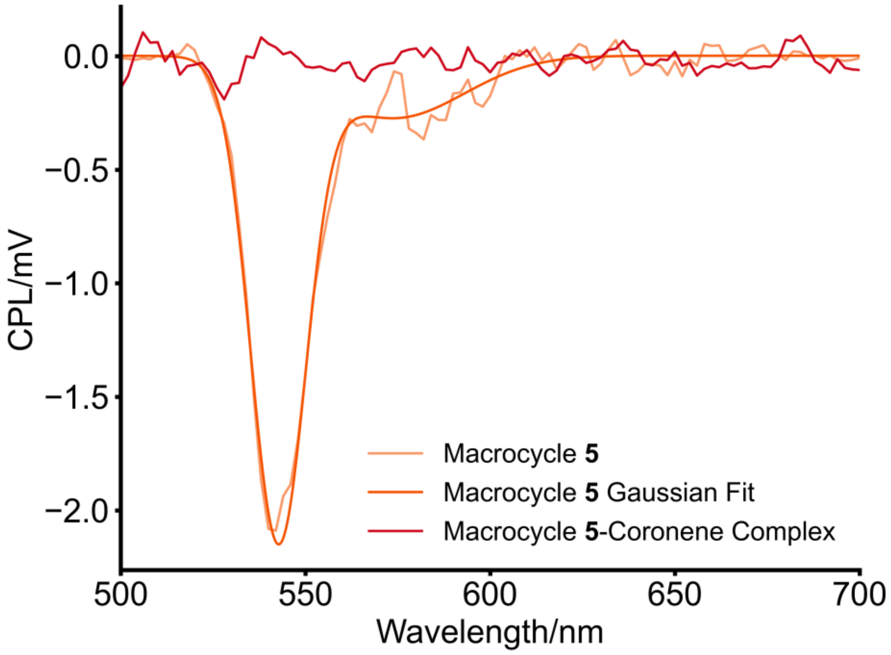

Supplementary Figure 4-37: CPL spectra of macrocycle **5** and **5**-coronene complex (~98% degree of complexation) in PhMe at 5 µM concentration, excited at 450 nm. The degree of complexation is known from the K_a value for coronene.

5) X-Ray Crystallography

General

Crystals for ValPDIMC_CHCl3 and ValPDIMC_CH2Cl2 were grown by vapour diffusion of hexane into concentrated solutions of **5** in $CHCl_3$ and CH_2Cl_2, respectively. Glass vials for crystallisation were coated with Sigmacote® siliconizing agent (Merck, SL2) prior to use. Crystals for ValPDIMC_CHCl3 were measured on a Rigaku Synergy-S dual source diffractometer with a PhotonJet-S X-ray source and a HyPix-6000 detector. Crystals for ValPDIMC_CH2Cl2 were measured on an Agilent SuperNova single crystal X-ray diffractometer with dual wavelength microfocus X-ray source and an Atlas CCD detector. Data were collected at 100 K through the use of an Oxford Cryosystems cryostream device using Cu K_α radiation. Data were collected and processed using CrysAlisPro 1.171.43.

Crystals for ValPDIMC_Pyrene, ValPDIMC_Perylene and ValPDIMC_Coronene were grown by vapour diffusion of hexane into concentrated solutions of **5** in $CHCl_3$ containing an excess of pyrene, perylene and coronene, respectively. Single crystal X-ray diffraction experiments for those crystals were performed by the UK EPSRC National Crystallography Service on a Rigaku 007HF diffractometer with HF Varimax confocal mirrors, an UG2 goniometer and HyPix 6000HE detector at 100 K using Cu K_α radiation and processed using CrysAlisPro 1.171.43.

The structures were solved by direct methods using ShelXT[4] and refined with ShelXL[5] using a least squares method. Olex2 software was used as the solution, refinement and analysis program.[6] Non-crystallographically-defined hydrogen atoms were placed geometrically and refined using a riding model. The crystal structure of ValPDIMC_Coronene showed some disorder on one of the straps which could not be accurately modelled.

Crystal data for ValPDIMC_CHCl3

$C_{78}H_{64}Cl_{12}N_4O_{16}$, M_r = 1738.73, triclinic, $P1$ (No. 1), a = 10.59280(10) Å, b = 12.4294(2) Å, c = 15.2662(2) Å, α = 91.1720(10)°, β = 90.0360(10)°, γ = 106.2870(10)°, V = 1928.87(4) Å^3, T = 100(2) K, Z = 1, Z' = 1, μ(Cu K$_\alpha$) = 4.536, 71688 reflections measured, 14832 unique (R_{int} = 0.0652) which were used in all calculations. The final wR_2 was 0.1877 (all data) and R_1 was 0.0706 (I > 2(I)).

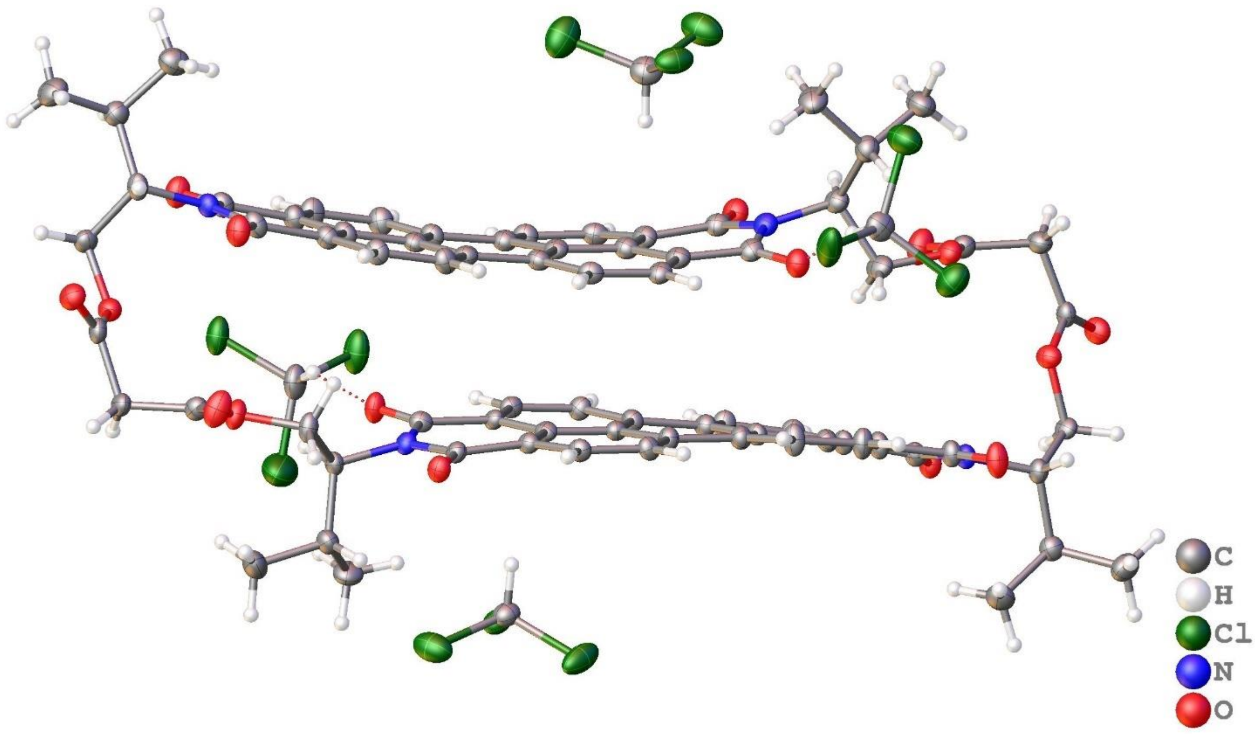

Supplementary Figure 5-1: Asymmetric Unit of ValPDI_MC_CHCl3. Thermal Ellipsoids show 50% Probability.

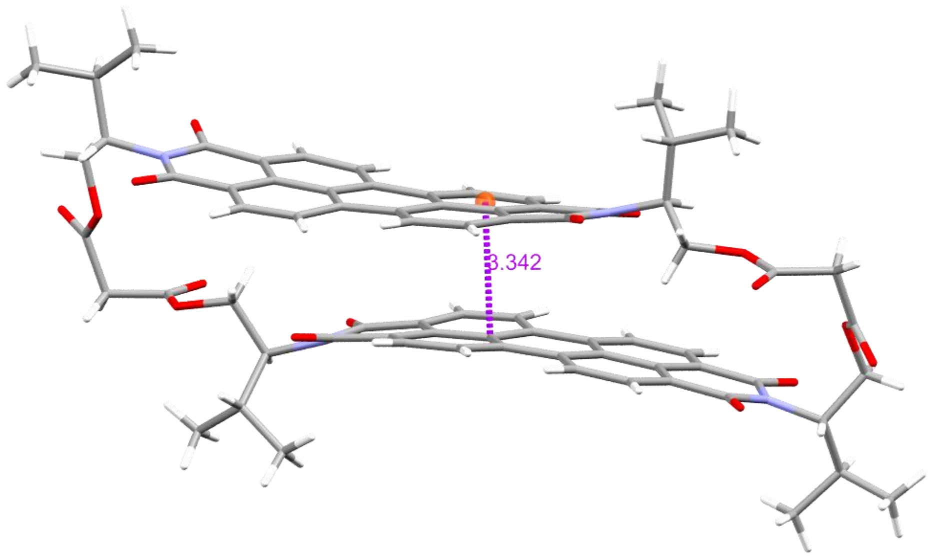

Supplementary Figure 5-2: Pi-stacking distance measurement from single crystal structure. Measurement was taken using Mercury using the crystal structure ValPDIMC_CHCl3. Solvent molecules were omitted for clarity.

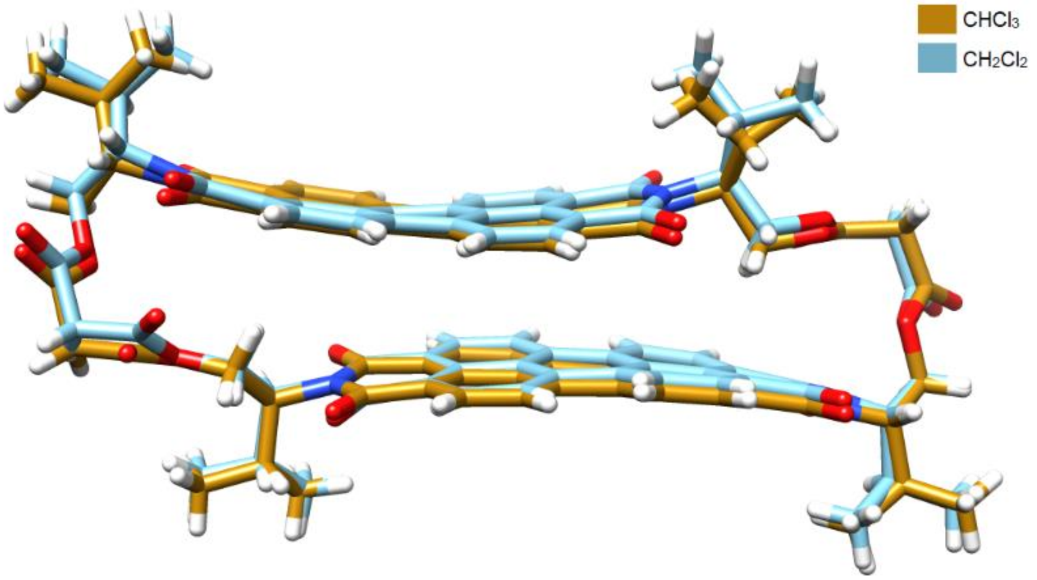

Supplementary Figure 5-3: Superimposed structures of macrocycle 5 from crystals obtained from $CHCl_3$ and CH_2Cl_2. There is a very close match between the structures obtained from both solvents. Solvent molecules from the crystal structures were omitted for clarity.

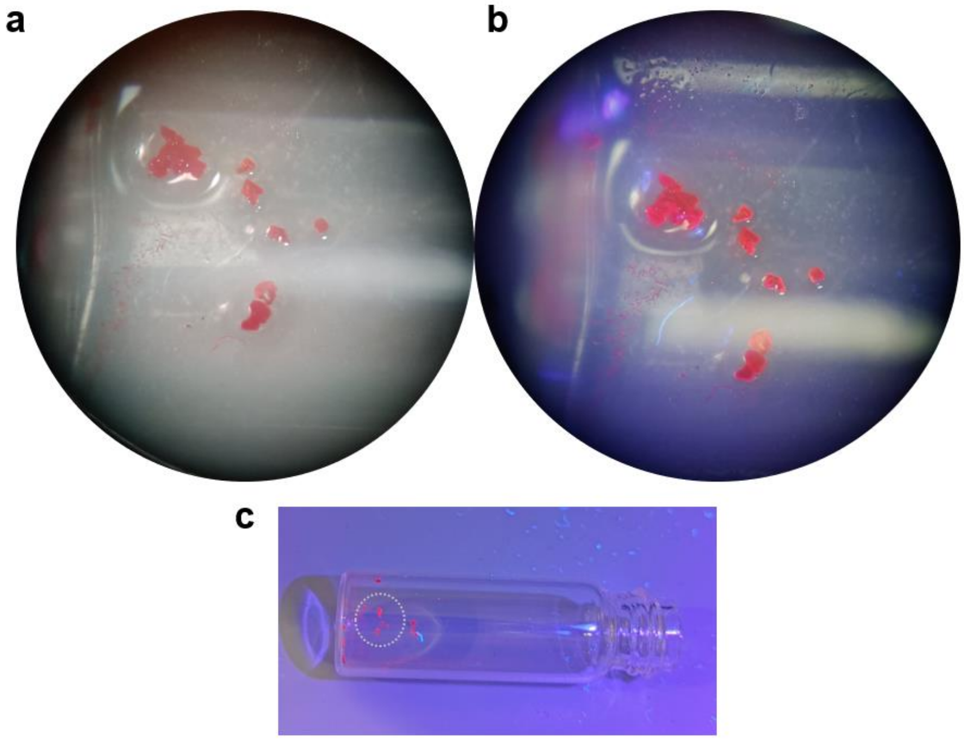

Supplementary Figure 5-4: Photographs of ValPDIMC_CHCl3 crystals through the lens of a benchtop microscope under ambient (**a**) or UV (365 nm) illumination (**b**), as well as a picture of the bulk vial with crystals inside under UV (365 nm) irradiation (**c**), with the crystal cluster photographed under the microscope highlighted.

Crystal data for ValPDIMC_CH2Cl2

$C_{76}H_{64}Cl_4N_4O_{16}$, M_r = 1431.11, triclinic, $P1$ (No. 1), a = 11.3132(4) Å, b = 11.9614(4) Å, c = 13.8223(4) Å, α = 67.395(3)°, β = 69.660(3)°, γ = 87.380(3)°, V = 1610.73(10) Å^3, T = 100(2) K, Z = 1, μ(CuK$_α$) = 2.321, 25322 reflections measured, 9217 unique (R_{int} = 0.0387) which were used in all calculations. The final wR_2 was 0.1502 (all data) and R_1 was 0.0554 (I > 2(I)).

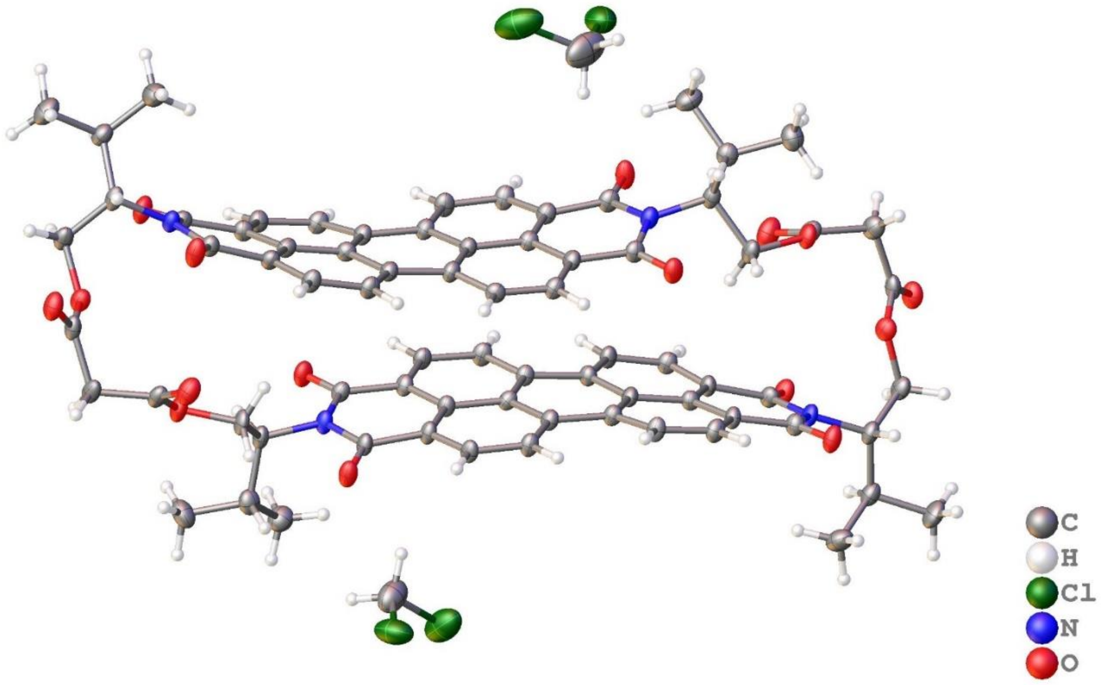

Supplementary Figure 5-5: Asymmetric Unit of ValPDIMC_CH2Cl2. Thermal Ellipsoids show 50% Probability.

Crystal data for ValPDIMC_Pyrene

$C_{106}H_{80}N_4O_{16}$, M_r = 1665.74, triclinic, *P*1 (No. 1), a = 9.4787(3) Å, b = 14.3045(4) Å, c = 15.1739(6) Å, α = 95.862(3)°, β = 104.136(3)°, γ = 92.284(3)°, *V* = 1980.18(12) Å^3, *T* = 100(2) K, *Z* = 1, *Z'* = 1, μ(Cu K_α) = 0.764, 55386 reflections measured, 12876 unique (R_{int} = 0.0519) which were used in all calculations. The final wR_2 was 0.1813 (all data) and R_1 was 0.0645 (I > 2(I)).

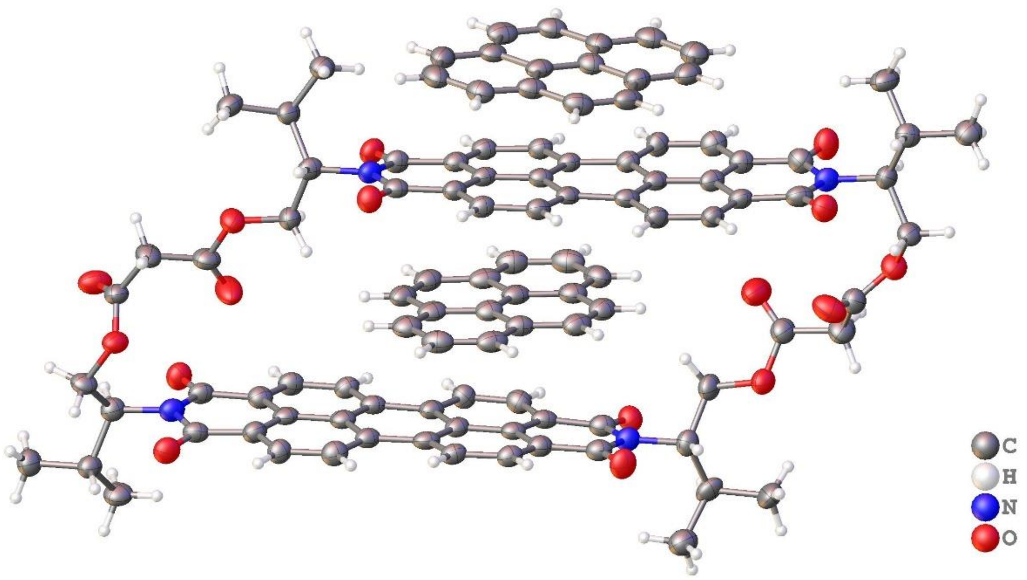

Supplementary Figure 5-6: Asymmetric Unit of ValPDIMC_Pyrene. Thermal Ellipsoids show 50% Probability

Crystal data for ValPDIMC_Perylene

$C_{114}H_{84}N_4O_{16}$, *Mr* = 1765.85, monoclinic, $P2_1$ (No. 4), a = 14.3636(2) Å, b = 9.6920(2) Å, c = 30.0293(7) Å,
β = 97.890(2)°, α = γ = 90°, *V* = 4140.86(14) Å^3, *T* = 100(2) K, *Z* = 2, *Z'* = 1, μ (Cu K$_α$) = 0.766, 49413 reflections measured, 15030 unique (R_{int} = 0.0497) which were used in all calculations. The final wR_2 was 0.1612 (all data) and R_1 was 0.0609 (I > 2(I)).

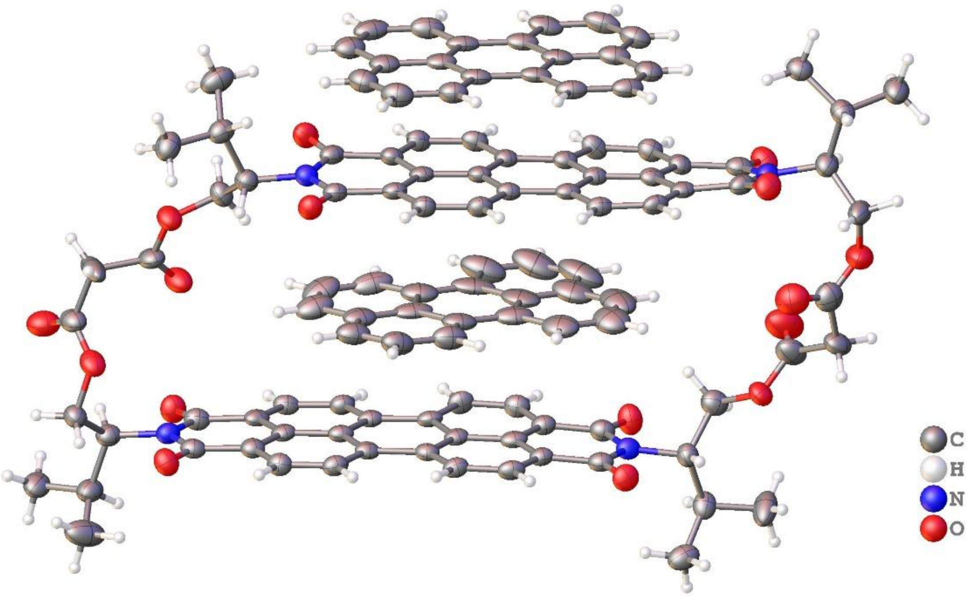

Supplementary Figure 5-7: Asymmetric Unit of ValPDI_MC_Perylene. Thermal Ellipsoids show 50% Probability.

Crystal Data for ValPDIMC_Coronene

$C_{122}H_{84}N_4O_{16}$, *Mr* = 1861.93, monoclinic, *P*21 (No. 4), a = 14.2531(2) Å, b = 10.97800(10) Å, c = 27.8626(4) Å, β = 94.7830(10)°, α = γ = 90°, *V* = 4344.49(10) $Å^3$, *T* = 100.00(2) K, *Z* = 2, *Z'* = 1, μ(Cu $K_α$) = 0.763, 142214 reflections measured, 14567 unique (*Rint* = 0.0407) which were used in all calculations. The final *wR2* was 0.1844 (all data) and *R1* was 0.0625 (I > 2(I)).

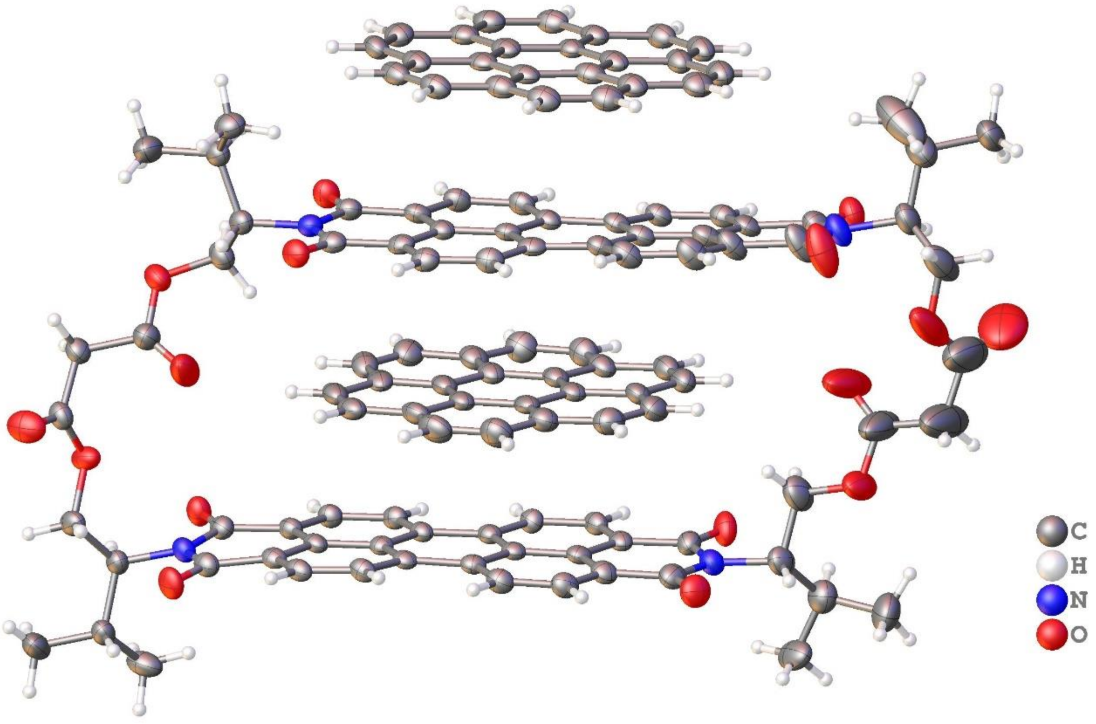

Supplementary Figure 5-8: Asymmetric Unit of ValPDI_MC_Coronene. Thermal Ellipsoids show 50% probability.

Overlay of Macrocycle components of Host/Guest Crystal Structures

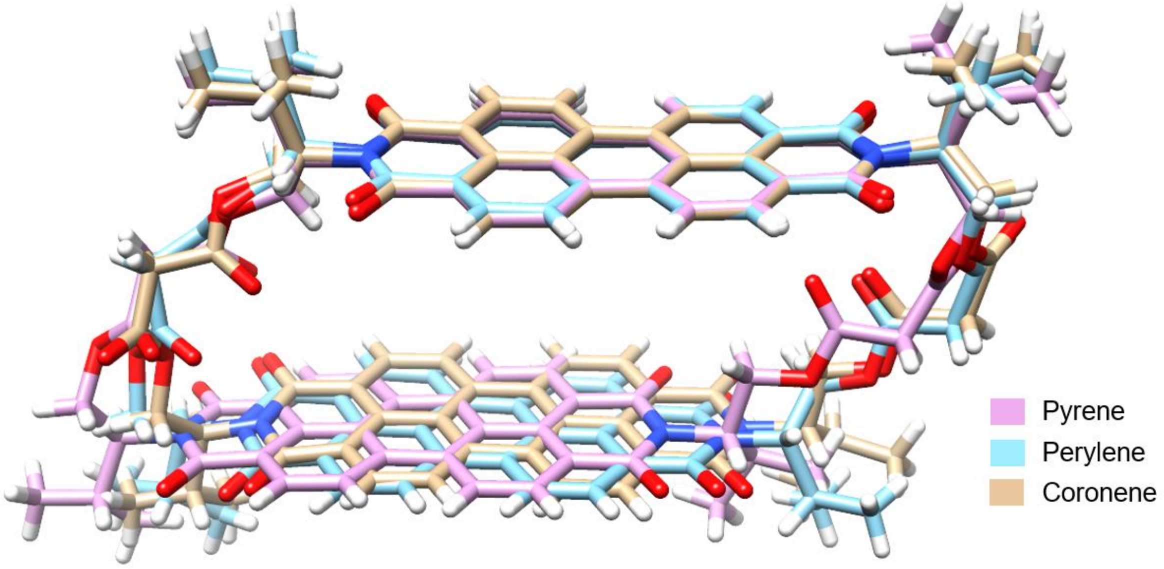

Supplementary Figure 5-9: Overlay of macrocycle parts of crystal structures with pyrene, perylene and coronene, with guest species removed.

6) Density Functional Theory

a) DFT optimisation of crystal structure

A conformation of macrocycle **5** was obtained by optimisation of a molecule taken from the experimental crystal structure. This calculation employed the B97-3c approach by Grimme and co-workers[7] and the COSMO solvation model[8] to describe solvation of the macrocycle in $CHCl_3$ (ε_r 4.81). This DFT optimised conformation is isoenergetic to that obtained from a conformer search and shows an intramolecular PDI dimer with M-helicity (**Supplementary Figure 6-1**). The .xyz file is provided in the 'DFT structures' folder.

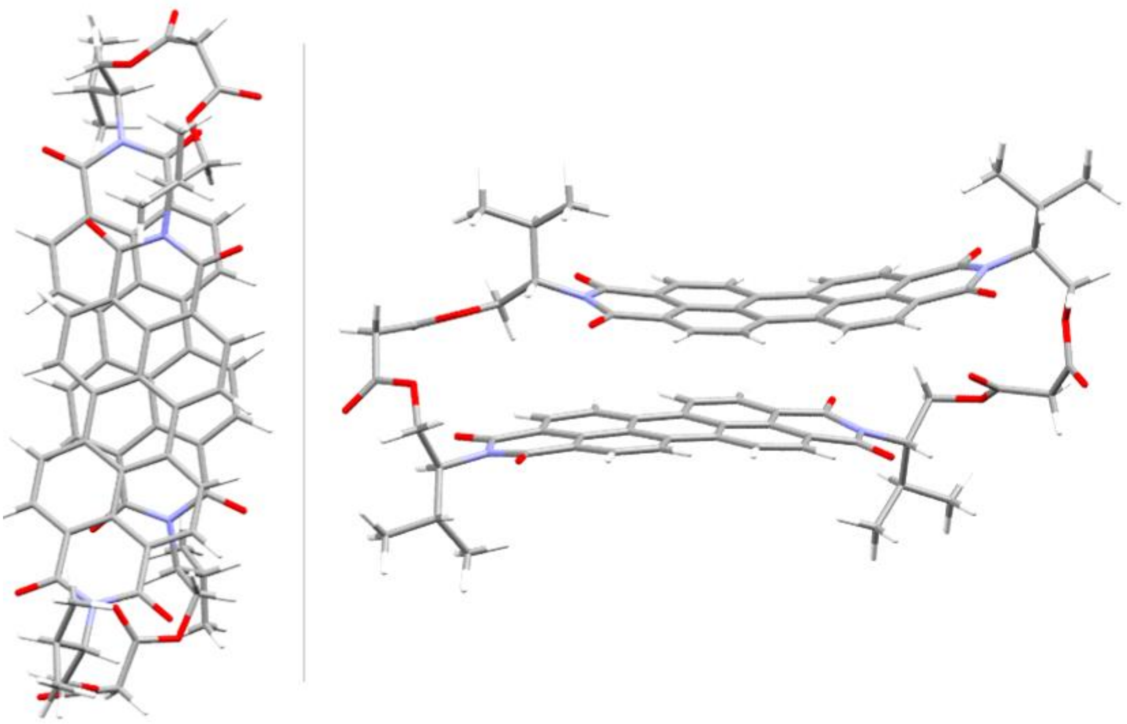

Supplementary Figure 6-1: Top and side view of the structure obtained by DFT optimisation of the macrocycle from the crystal structure with implicit solvent modelling in $CHCl_3$. The left-handed (M) helical arrangement of the PDIs is consistent with experimental results in organic solvents.

b) Conformer search

A conformer search in $CHCl_3$ for macrocycle **5** was performed using the combination of the CREST code[9] and the GFN2-xTB semiempirical tight-binding method.[10,11] The lowest energy conformer found using CREST is shown in **Supplementary Figure 6-2** and provided as a .xyz file in the 'DFT structures' folder. After reoptimization of this structure using DFT this conformer is isoenergetic to the conformer obtained from the experimental crystal structure.

Lowest Energy Conformer predicted by CREST

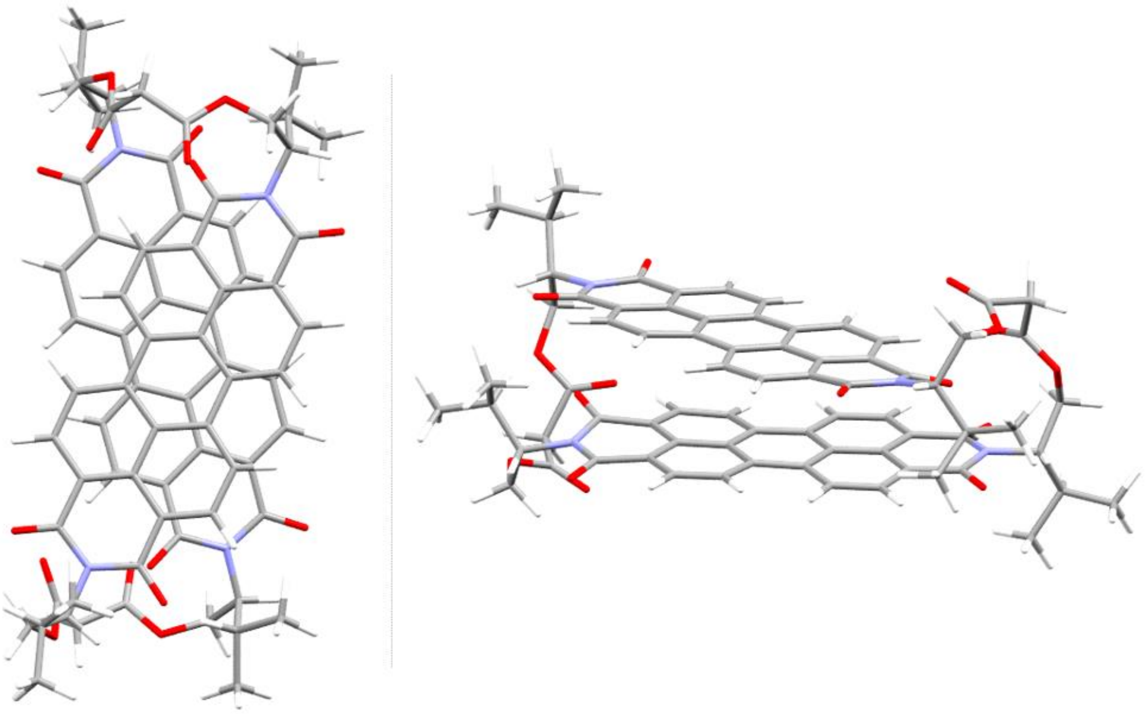

Supplementary Figure 6-2: Top and side view of the lowest energy conformer of macrocycle **5** as predicted by CREST. There is a left-handed (M) helical arrangement of the PDIs which is consistent with experimental results in organic solvents.

c) Predicted UV-vis and CD spectra of left-handed (M) conformer

Vertical excitation and circular dichroism spectra of all conformations of macrocycle **5** were predicted using TD-DFT in $CHCl_3$. These calculations employed the TD-ωB97x[12] functional, the def2-SVP basis-set,[13] and the COSMO solvation model,[8] and were performed on top of the B97-3c optimised structures.

Supplementary Table 6-1: Predicted UV-vis absorption spectrum and rotary strengths in the CD spectrum of the conformer of the macrocycle obtained by optimisation of the crystal structure in $CHCl_3$ by time dependent-DFT.

Excitation	Energy (nm)	Oscillator strength	Rotary strength
1	473.671	0.15656	-292.867
2	470.961	1.51597	398.690
3	375.793	0.03336	1.311
4	359.272	0.28569	-37.251
5	328.747	0.00122	-17.817
6	318.765	0.00034	-0.758

Supplementary Table 6-2: Predicted UV-vis absorption spectrum and rotary strengths in the CD spectrum of the lowest energy conformer of the macrocycle from the CREST conformer search in $CHCl_3$ by time dependent-DFT.

Excitation	Energy (nm)	Oscillator strength	Rotary strength
1	482.141	0.19137	-888.541
2	449.404	1.45677	1092.275
3	388.463	0.00604	-34.375
4	388.186	0.04236	9.602
5	321.628	0.00327	5.859
6	319.055	0.00001	0.286

Comparison of predicted and experimental CD spectra

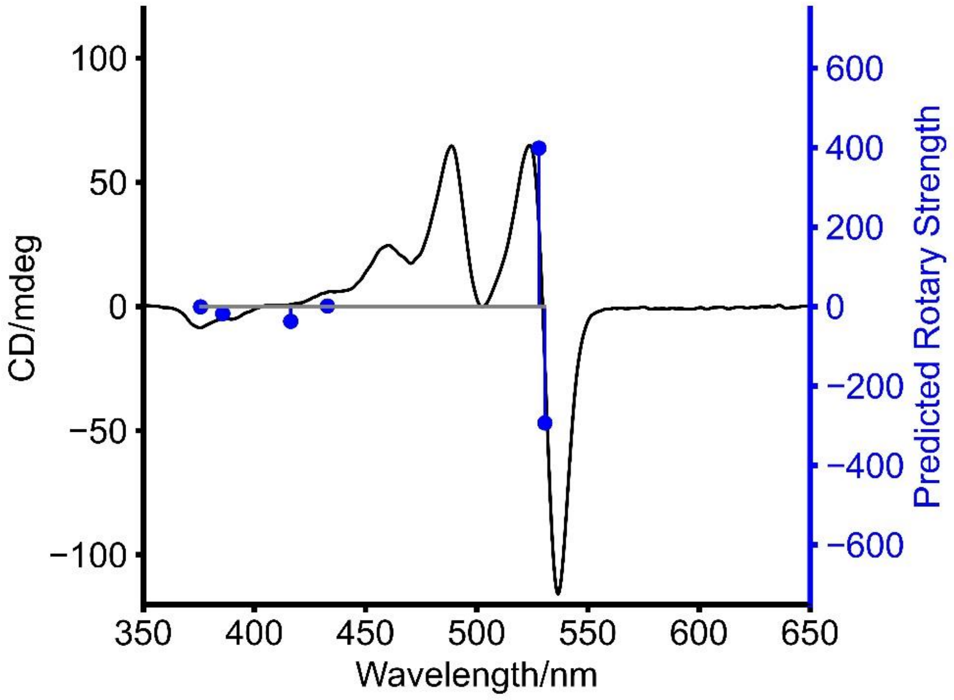

Supplementary Figure 6-3: Predicted (optimisation of the crystal structure in $CHCl_3$) and experimental CD spectra for macrocycle **5**. The calculated rotatory maxima (**Supplementary Table 6-1**) have been shifted by +57 nm since predicted CD spectra are blue-shifted, which is a side-effect of the range-separated functional used.

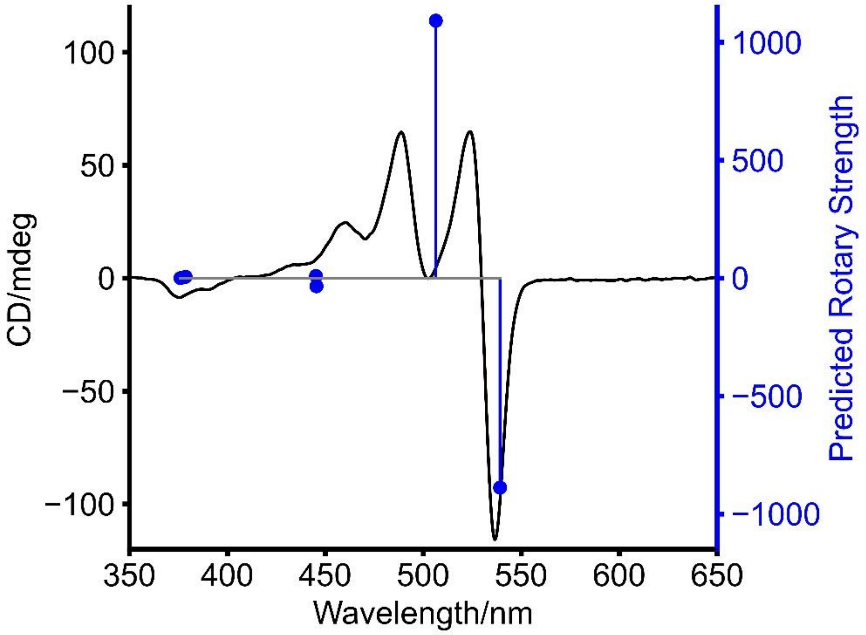

Supplementary Figure 6-4: Predicted (lowest energy conformer of the macrocycle from the CREST conformer search in $CHCl_3$) and experimental CD spectra for macrocycle **5**. The calculated rotatory maxima (**Supplementary Table 6-2**) have been shifted by +57 nm since predicted CD spectra are blue-shifted, which is a side-effect of the range-separated functional used.

d) Optimisation of geometry of right-handed (P) conformer by DFT

The initial structure of the right-handed (P) conformer was obtained through manual rotation of one of the PDI units in the crystal structure, followed by force-field optimisation. The resulting structure was then optimised by DFT in implicit H_2O using the B97-3c[7] method and the COSMO solvation model.[8]

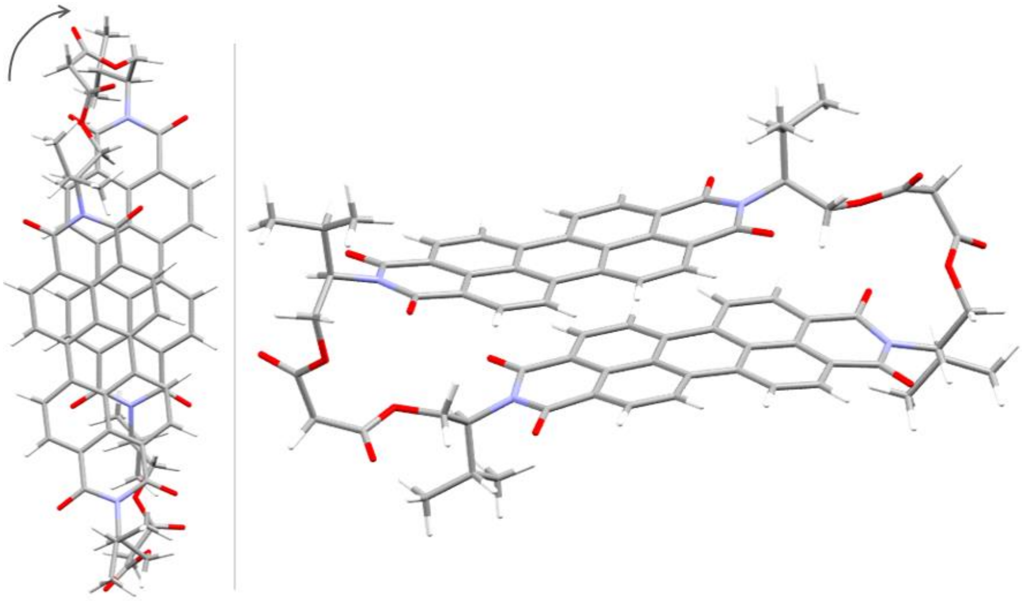

Supplementary Figure 6-5: Top and side view of the structure obtained by DFT optimisation of the macrocycle with a right-handed rotation in H_2O.

e) Predicted UV-vis and CD spectra of right-handed (P) conformer

Vertical excitation and circular dichroism spectra of the right-handed (P) conformer were predicted using TD-DFT in H_2O. These calculations employed the TD-ωB97x[12] functional, the def2-SVP basis-set,[13] and the COSMO solvation model,[8] and were performed on top of the B97-3c optimised structure.

Supplementary Table 2-3: Predicted UV-vis absorption spectrum and rotary strengths in the CD spectrum of the right-handed conformer of the macrocycle in H_2O by time dependent-DFT.

Excitation	Energy (nm)	Oscillator strength	Rotary strength
1	472.648	0.00393	107.456
2	450.467	2.06201	-123.631
3	370.085	0.00617	-14.549
4	368.050	0.00727	16.593
5	318.678	0.00006	5.597
6	317.746	0.00011	0.152

Comparison of predicted and experimental CD spectra

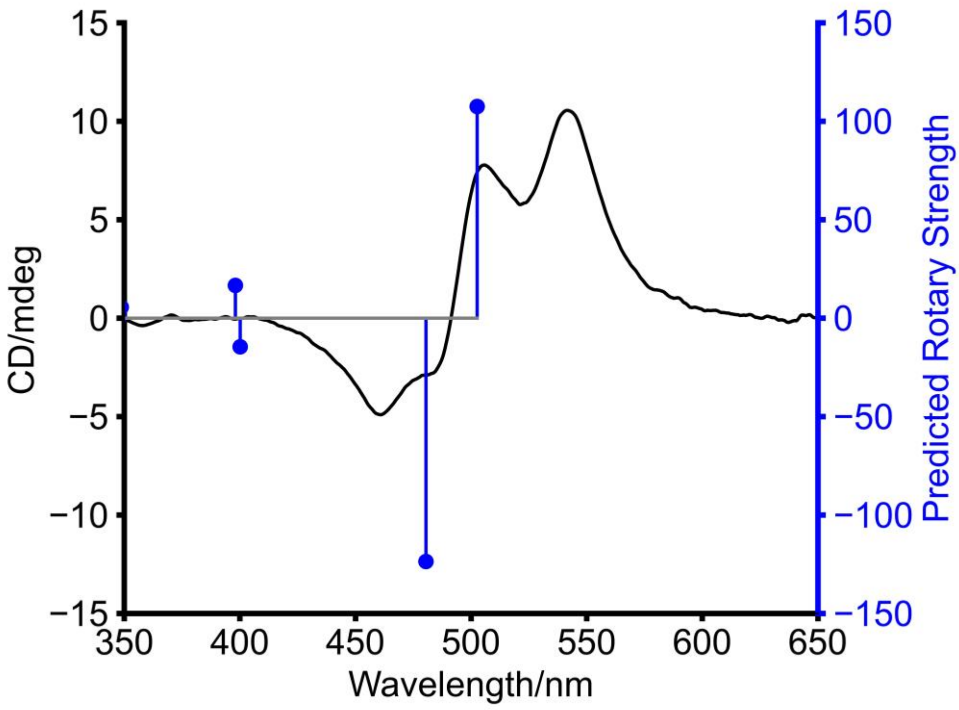

Supplementary Figure 6-6: Predicted (DFT optimised right-handed structure) and experimental CD spectra for macrocycle **5** in H_2O. The calculated rotatory maxima (**Supplementary Table 6-3**) have been shifted by +30 nm since predicted CD spectra are blue-shifted, which is a side-effect of the range-separated functional used.

7) NMR Spectra

L-Valinol-PDI 1

^{1}H-NMR (400 MHz, $CDCl_3$)

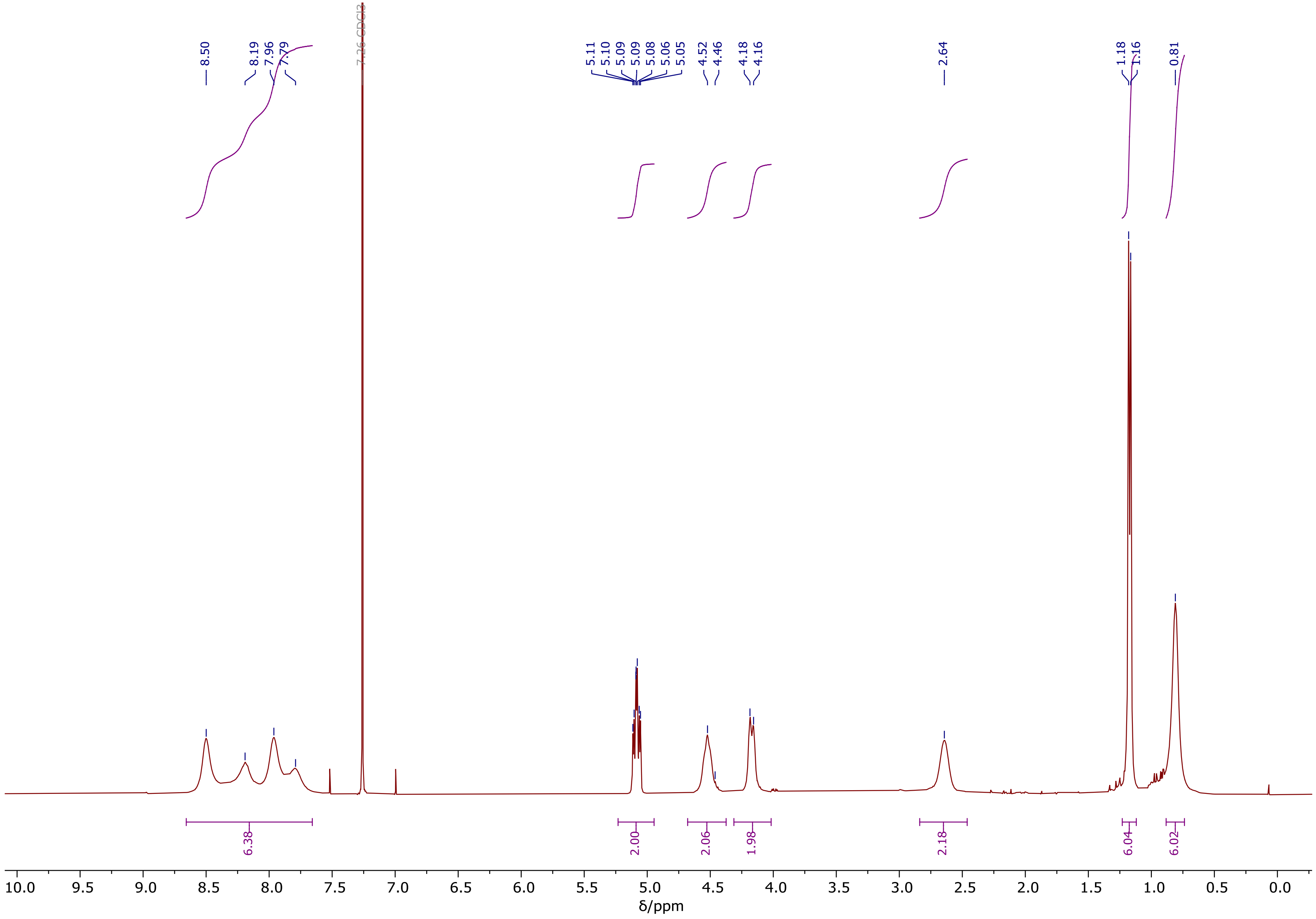

Di-TBDMS-L-Valinol-PDI 2

^{1}H-NMR (400 MHz, $CDCl_3$)

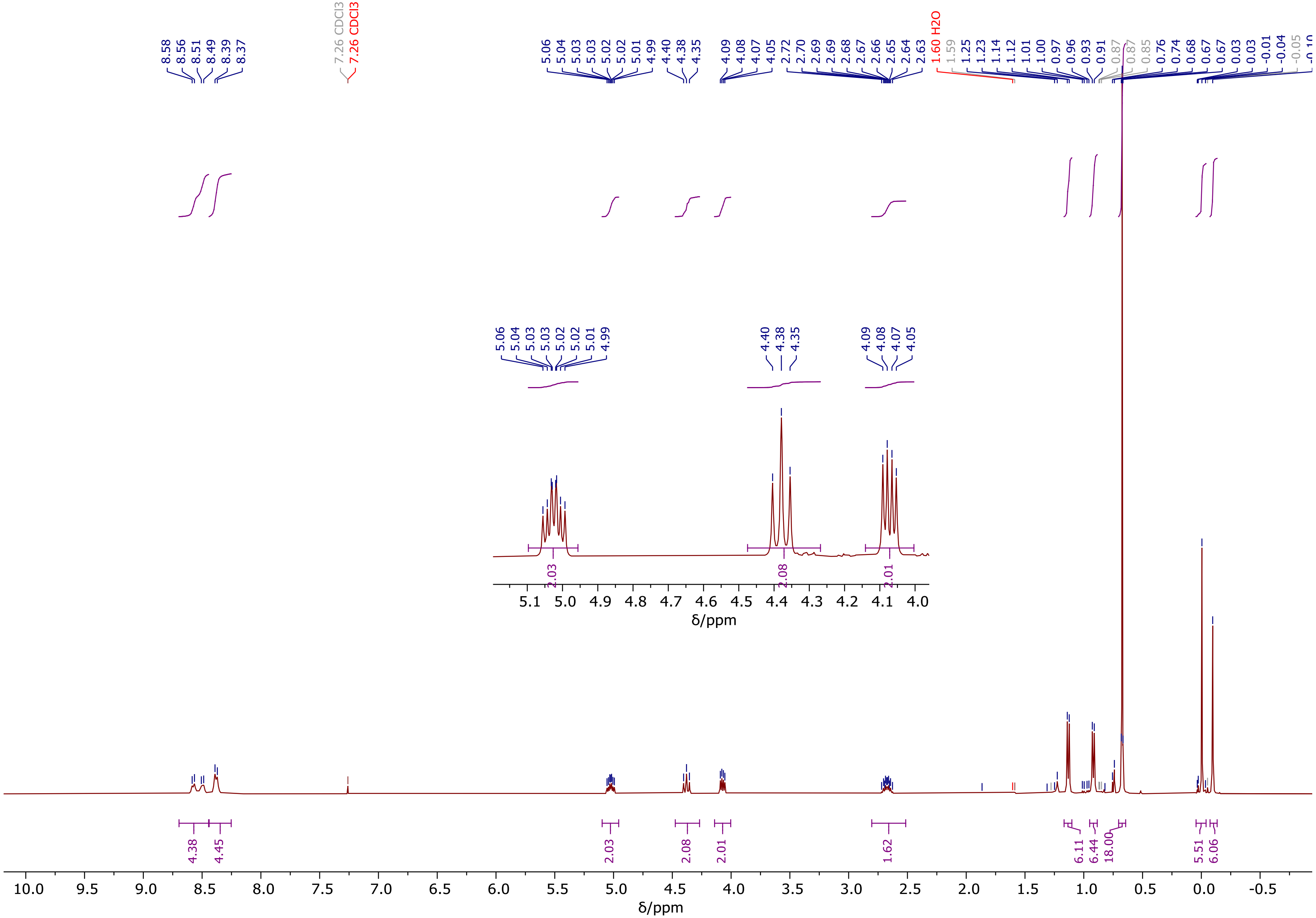

^{13}C-NMR (101 MHz, $CDCl_3$)

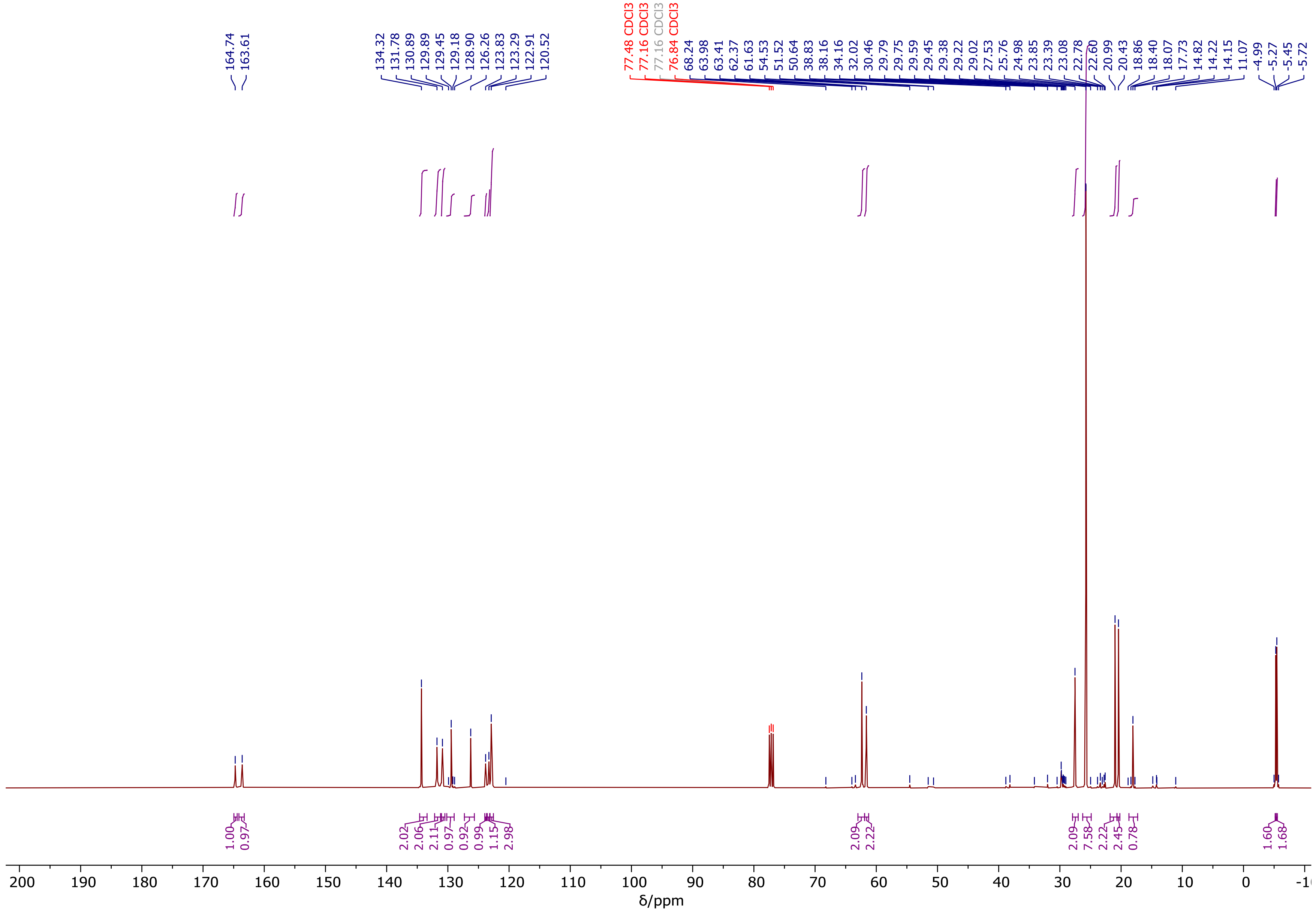

Mono-TBDMS-LValinol-PDI 3

^{1}H-NMR (300 MHz, $CDCl_3$)

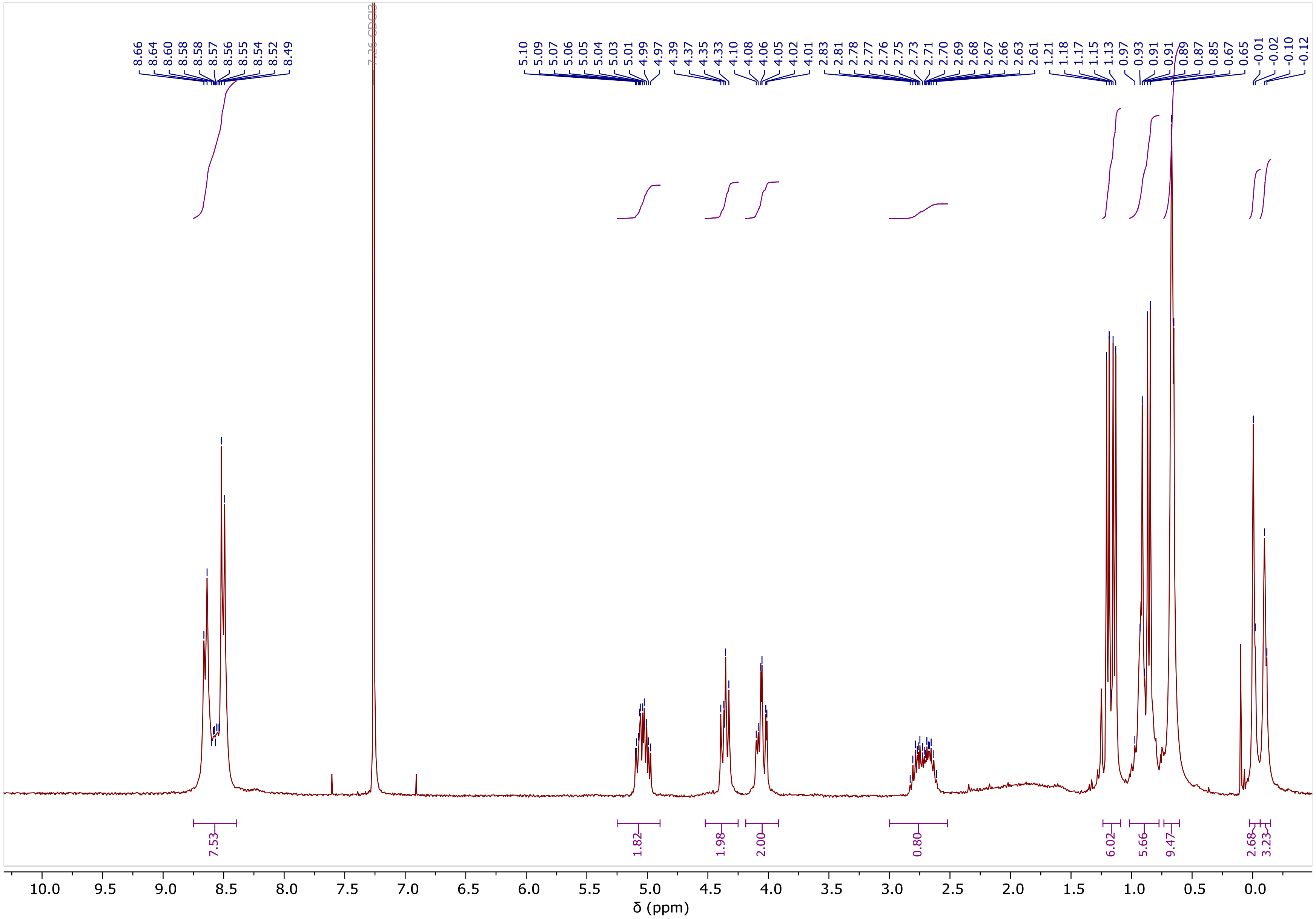

^{13}C-NMR (126 MHz, $CDCl_3$)

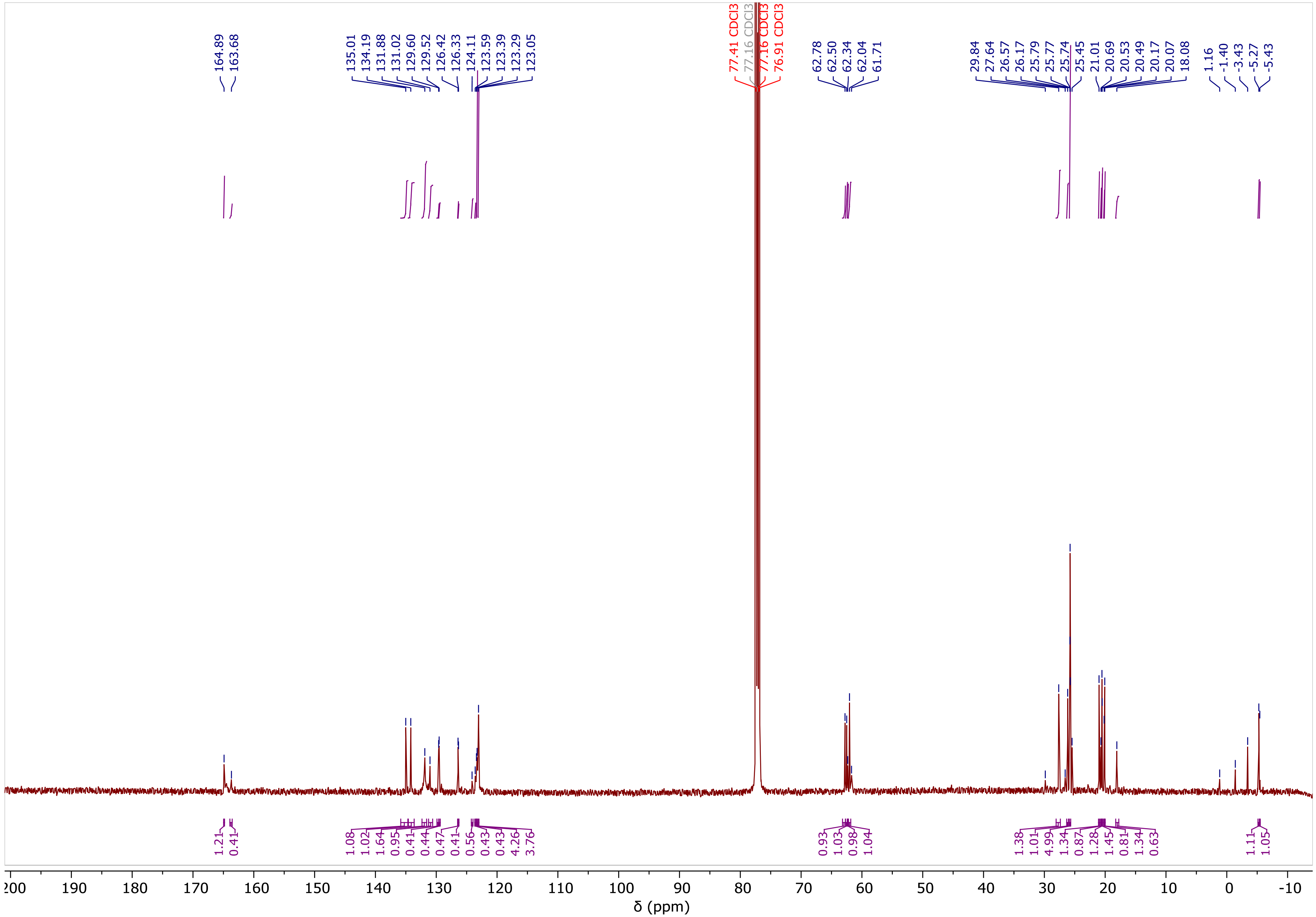

Di-TBDMS-LValinol-PDI Malonate 4

^{1}H-NMR (500 MHz, $CDCl_3$)

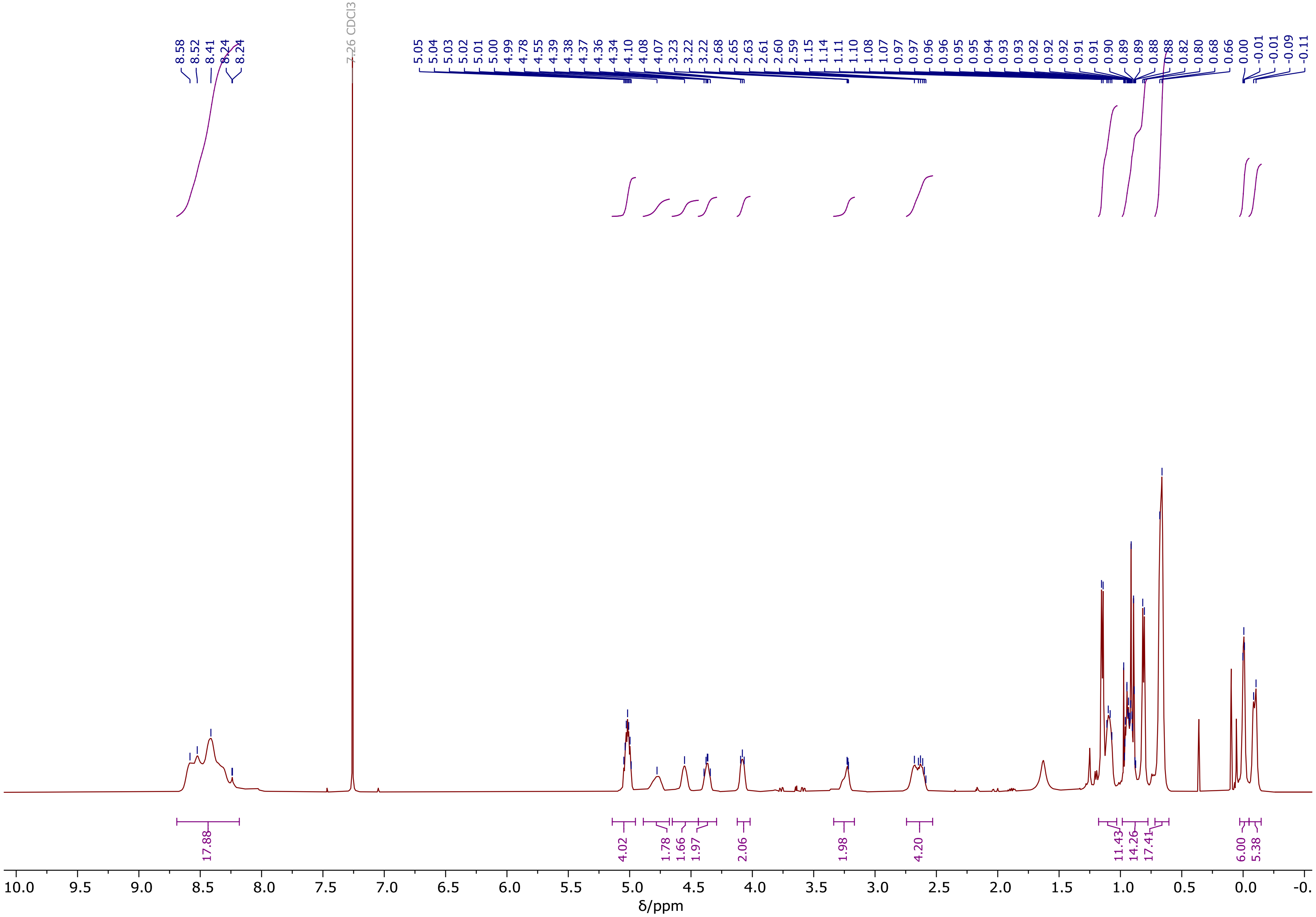

^{13}C-NMR (101 MHz, $CDCl_3$)

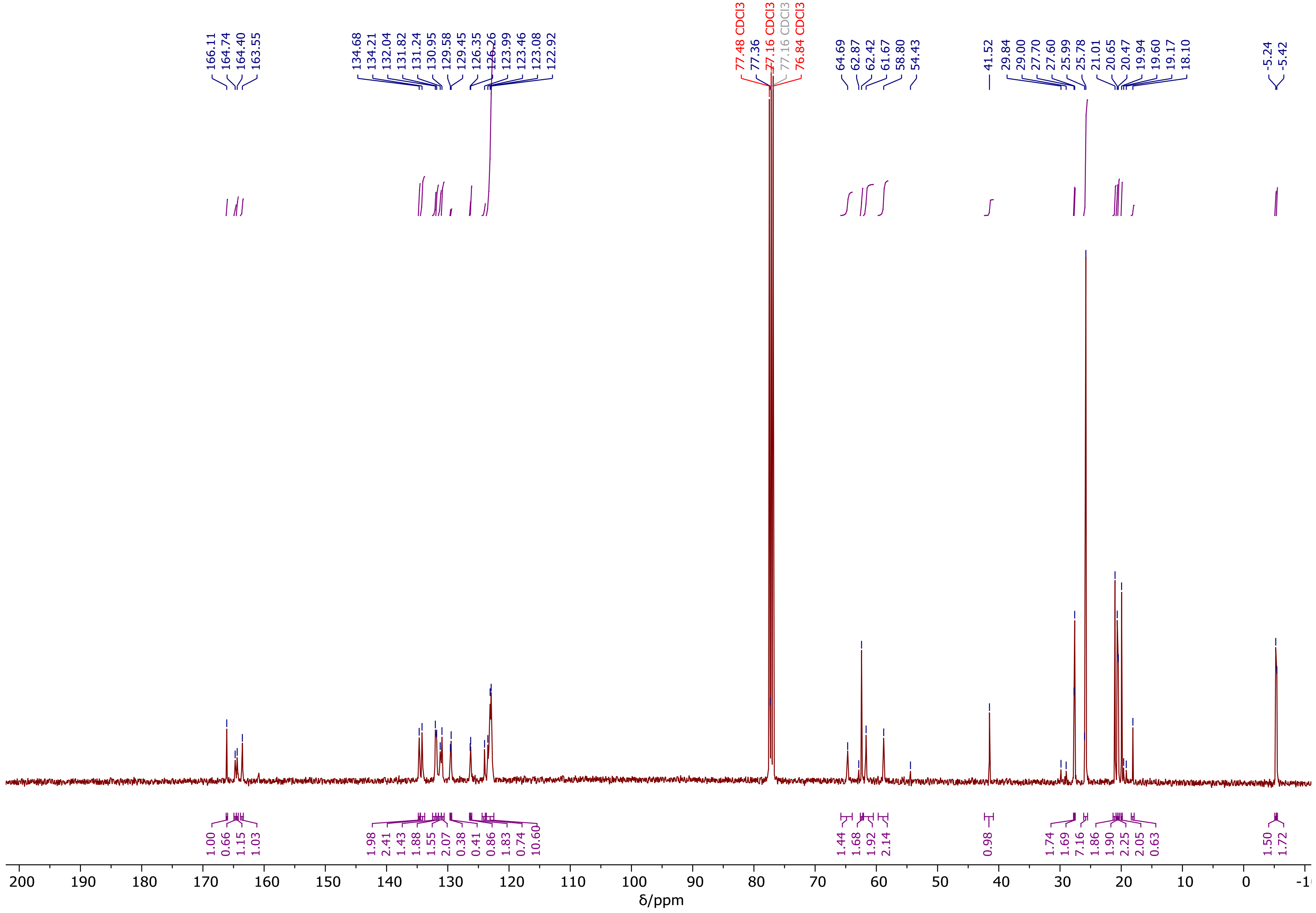

L-Valinol-Perylene-Diimide-Malonate Macrocycle 5

^{1}H-NMR (400 MHz, $CDCl_3$)

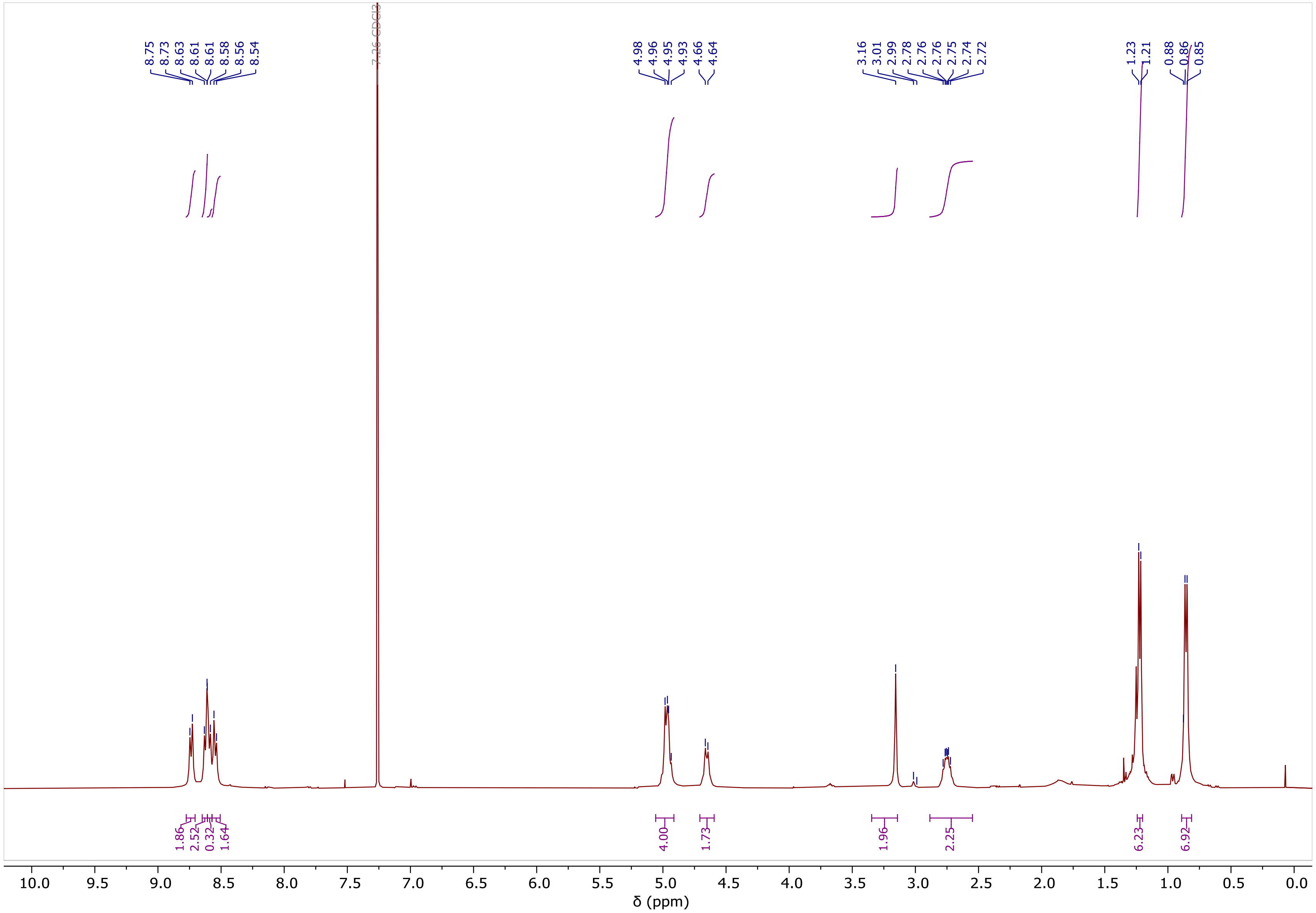

^{13}C-NMR (126 MHz, $CDCl_3$)

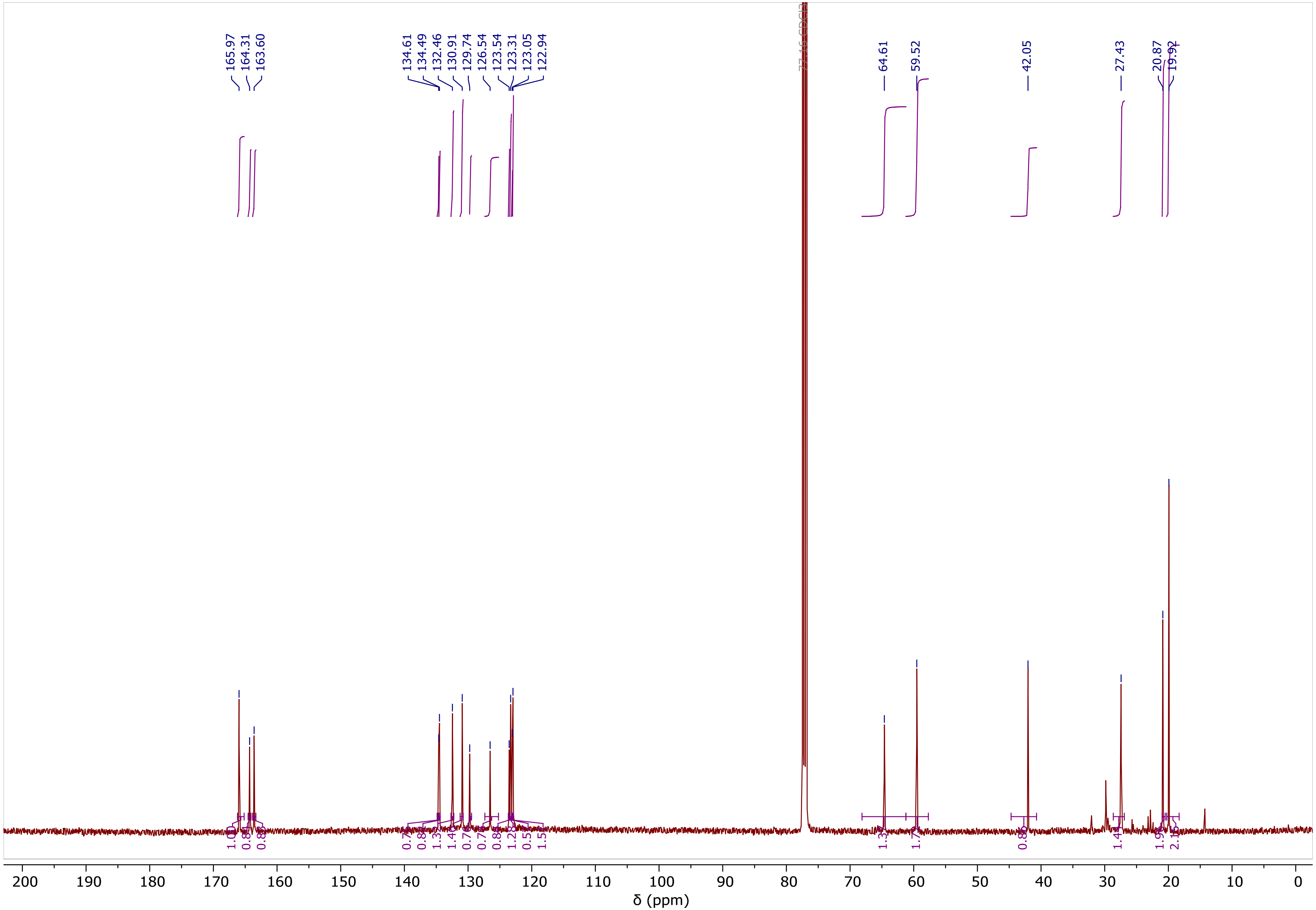

D-Valinol-Perylene Diimide (PDI) 6

^{1}H-NMR (400 MHz, $CDCl_3$)

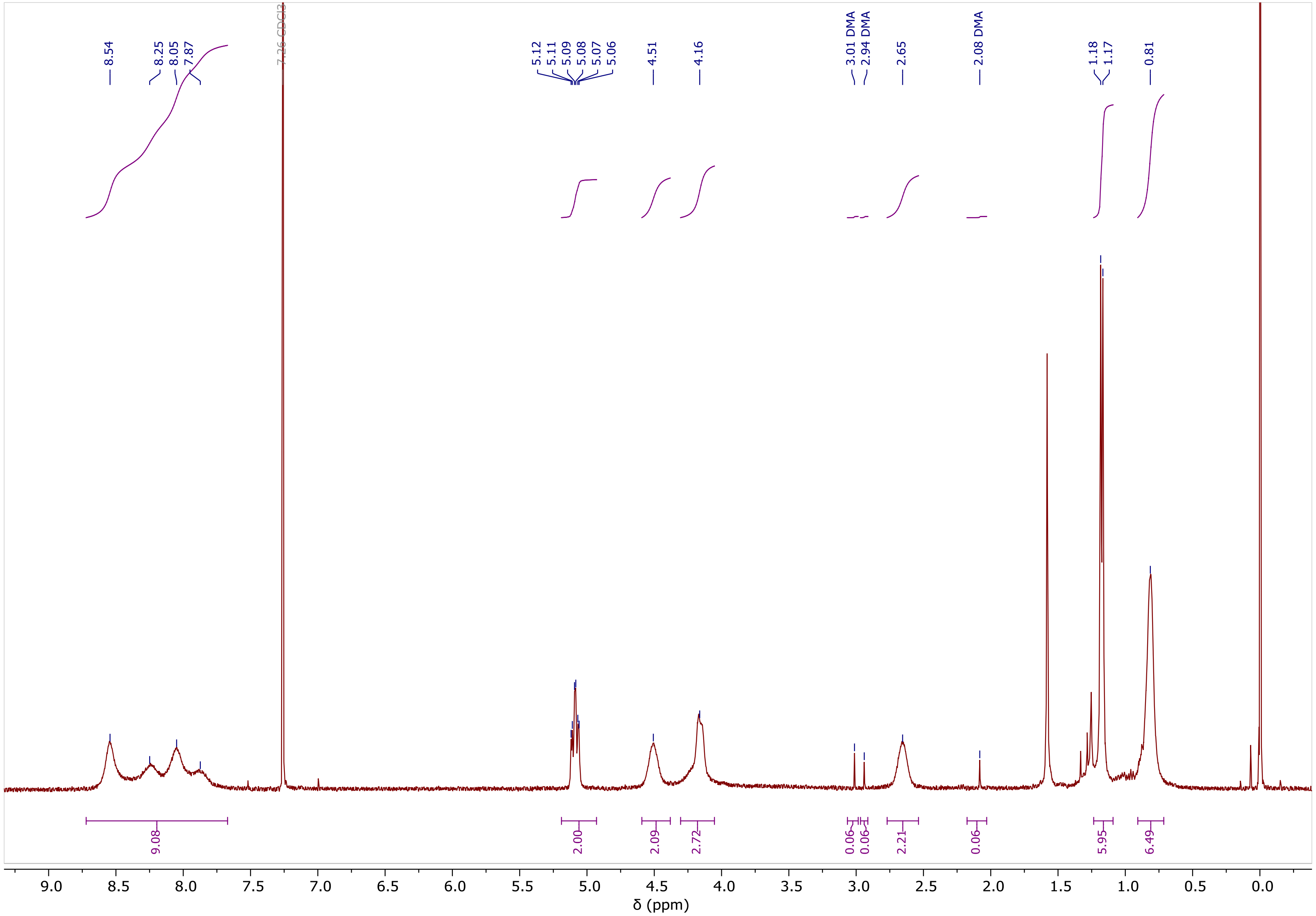

D-Valinol-Perylene Diimide Macrocycle 7

^{1}H-NMR (400 MHz, $CDCl_3$)

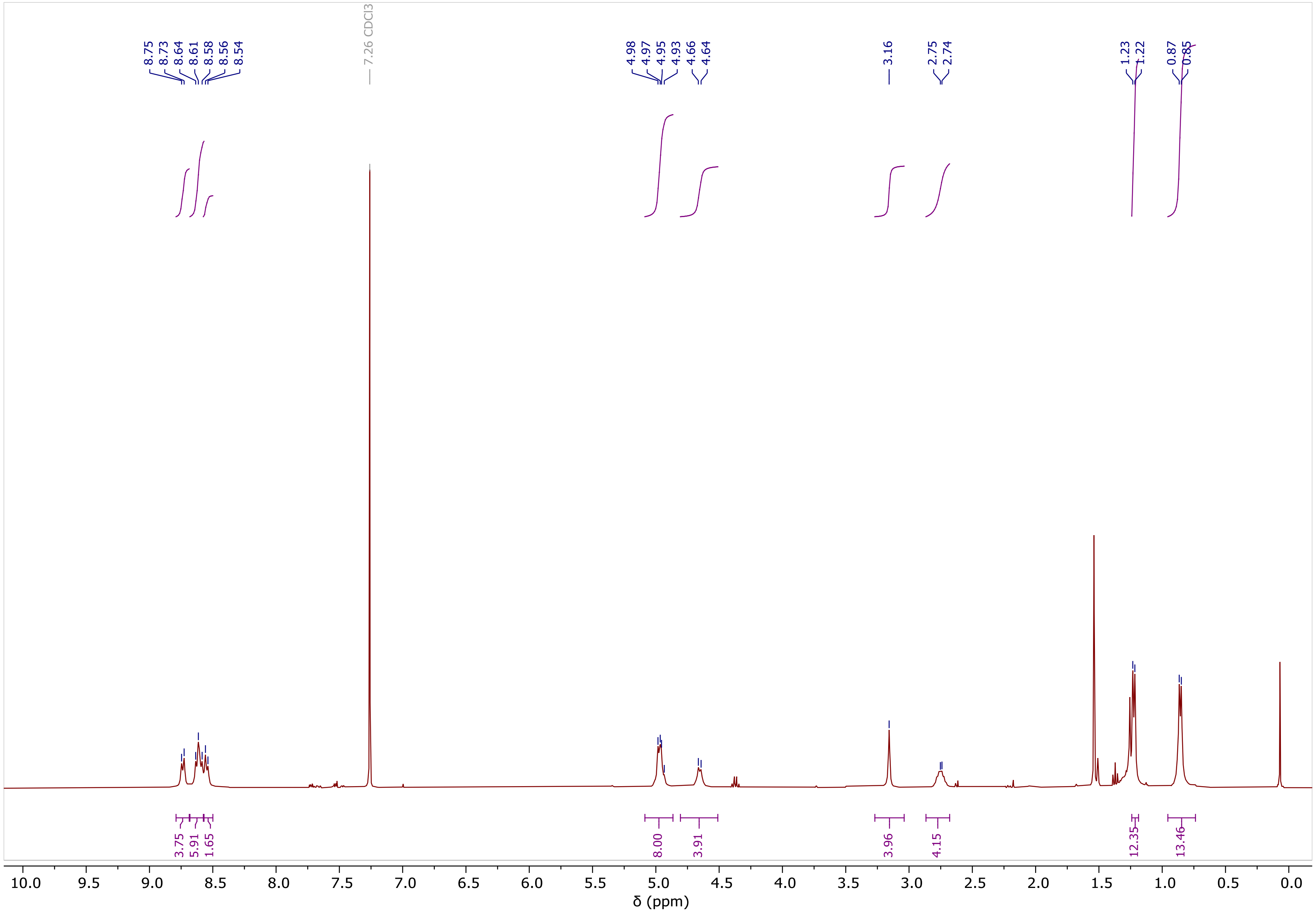

^{13}C-NMR (101 MHz, CDCl$_3$)

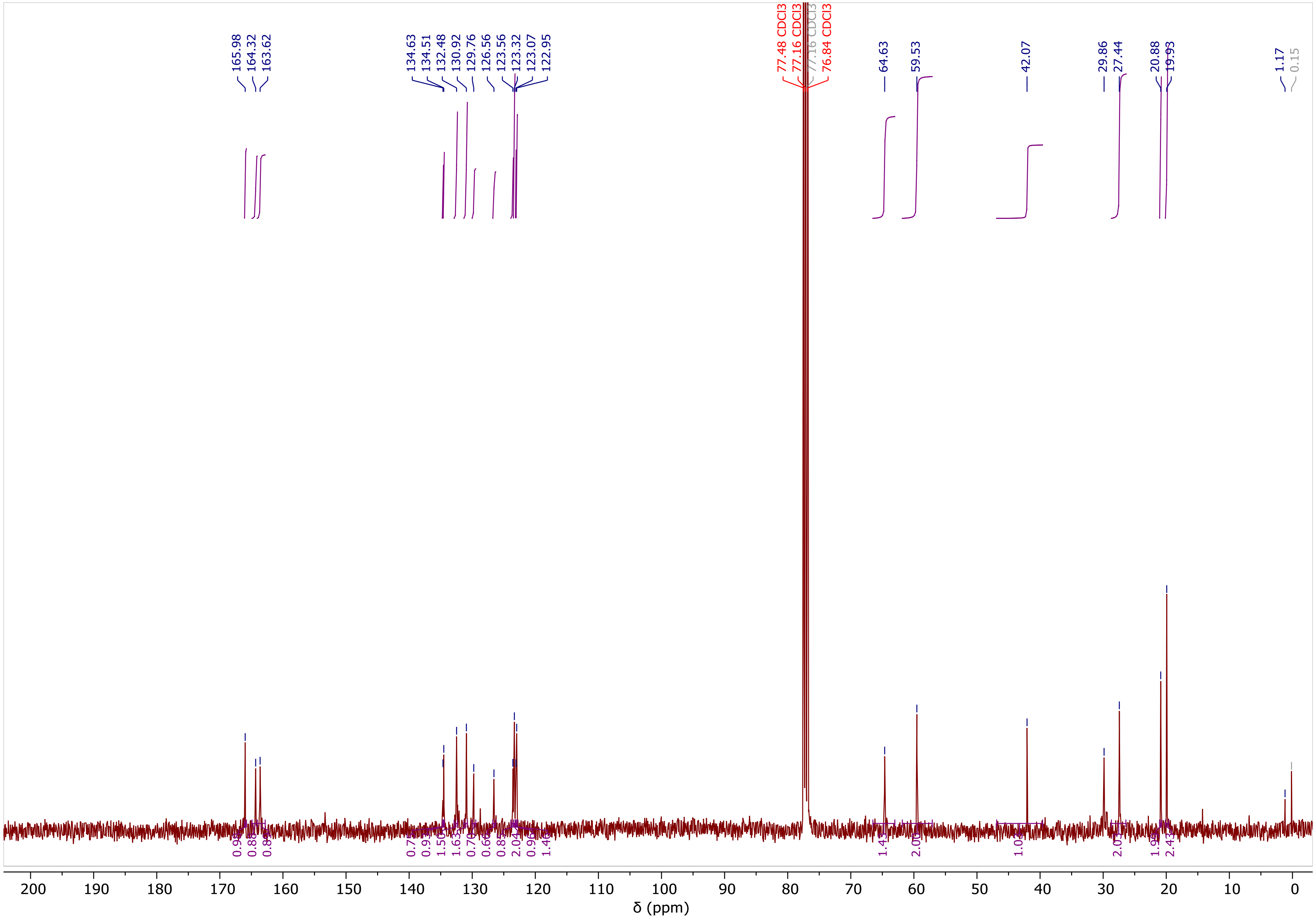

NMR Spectrum of Macrocycle 5 in DMSO

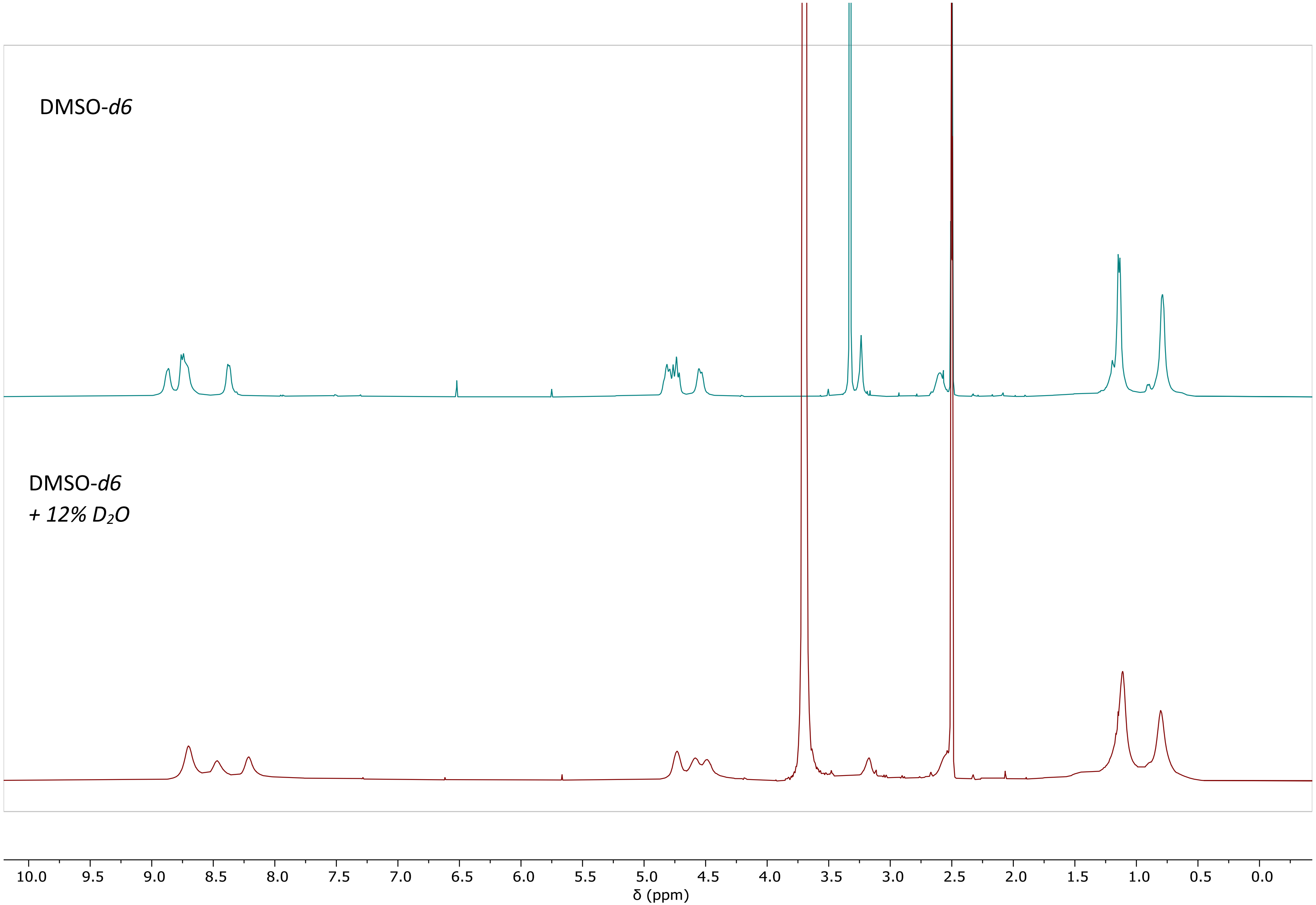

Supplementary Figure 7-1: NMR Spectra of macrocycle **5** in DMSO-*d6* and with 12% D_2O added

NOESY/EXSY in DMSO

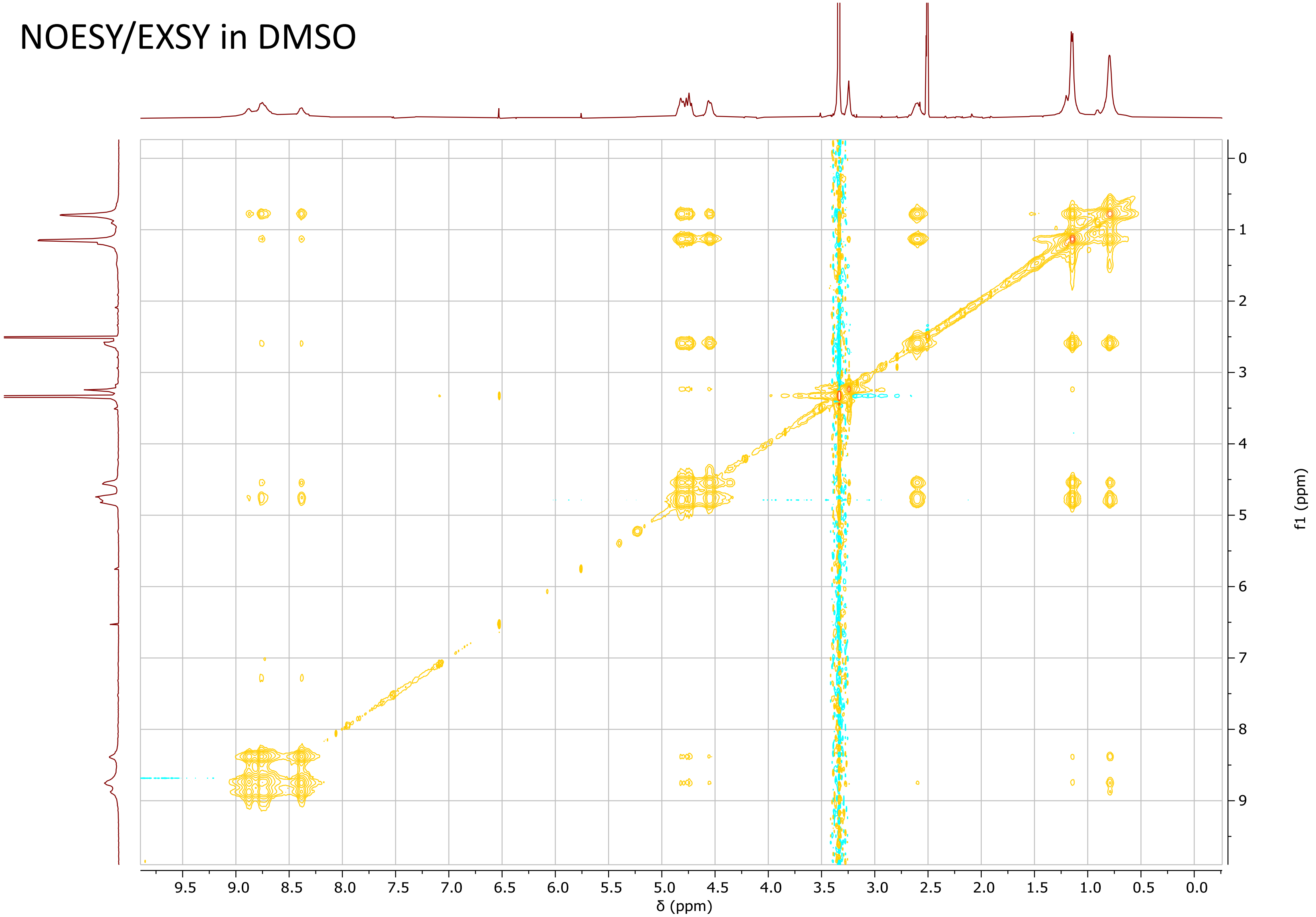

Supplementary Figure 7-2: NOESY/EXSY NMR Spectrum of macrocycle **5** in DMSO-*d6*

NOESY/EXSY in DMSO+D_2O

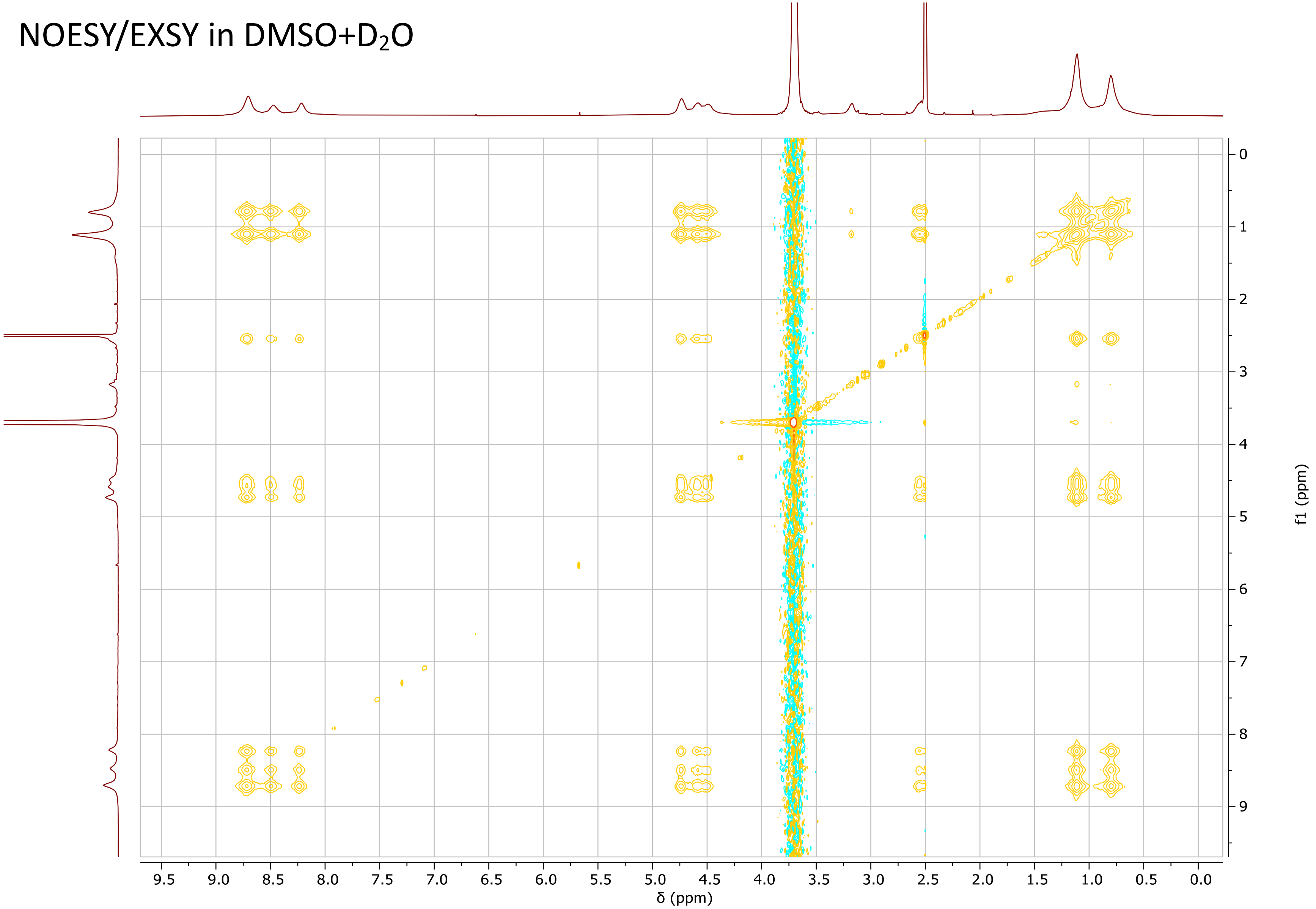

Supplementary Figure 7-3: NOESY/EXSY NMR Spectrum of macrocycle **5** in DMSO-*d6* + 12% D_2O. Despite the dilution of the sample with D_2O, the peak intensities increased, when scaled to the maximum peak.

NMR Spectrum of Macrocycle 5 in $CHCl_3$ without and with Coronene as Guest

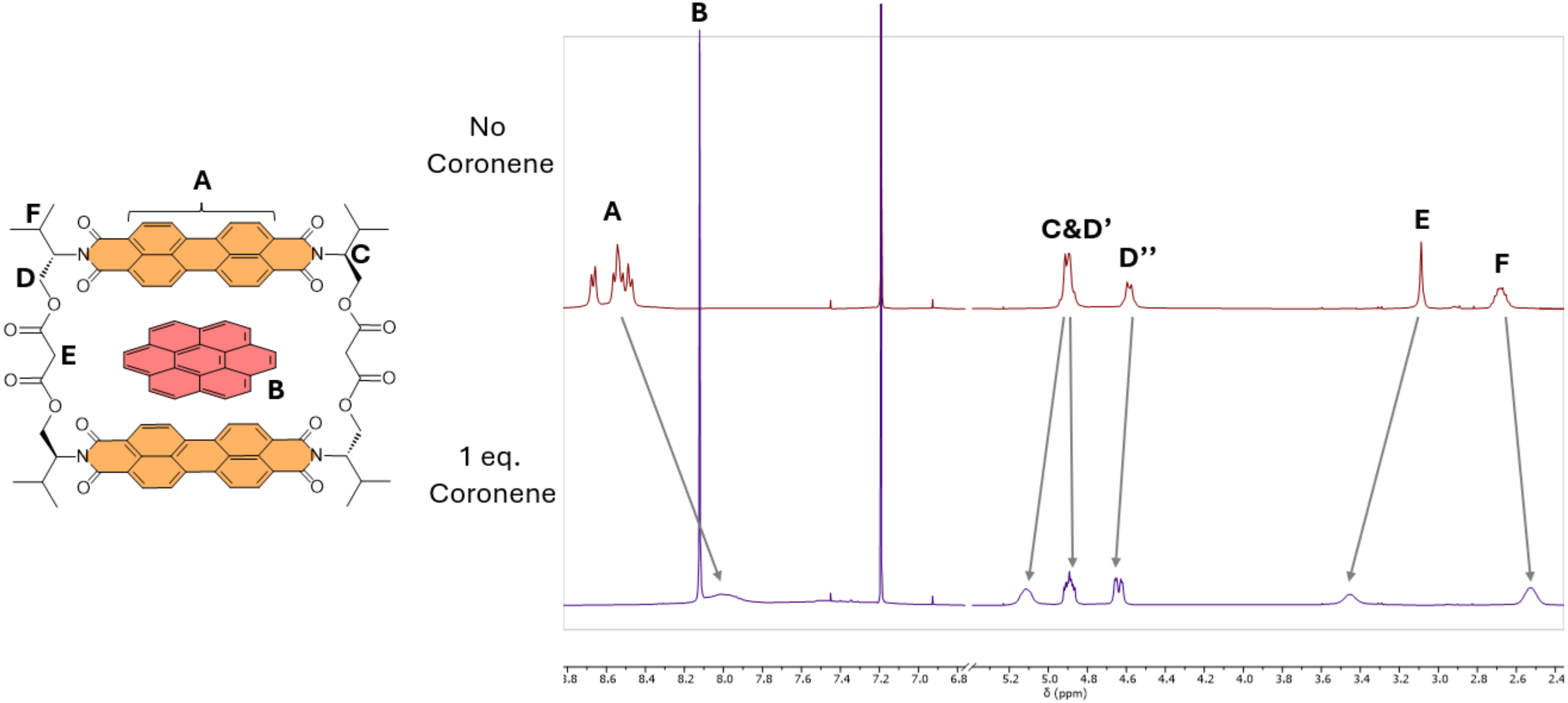

Supplementary Figure 7-4: Changes in 1H NMR of macrocycle **5** in $CDCl_3$ upon addition of 1 eq. of coronene

8) Mass Spectra

L-Valinol-PDI 1

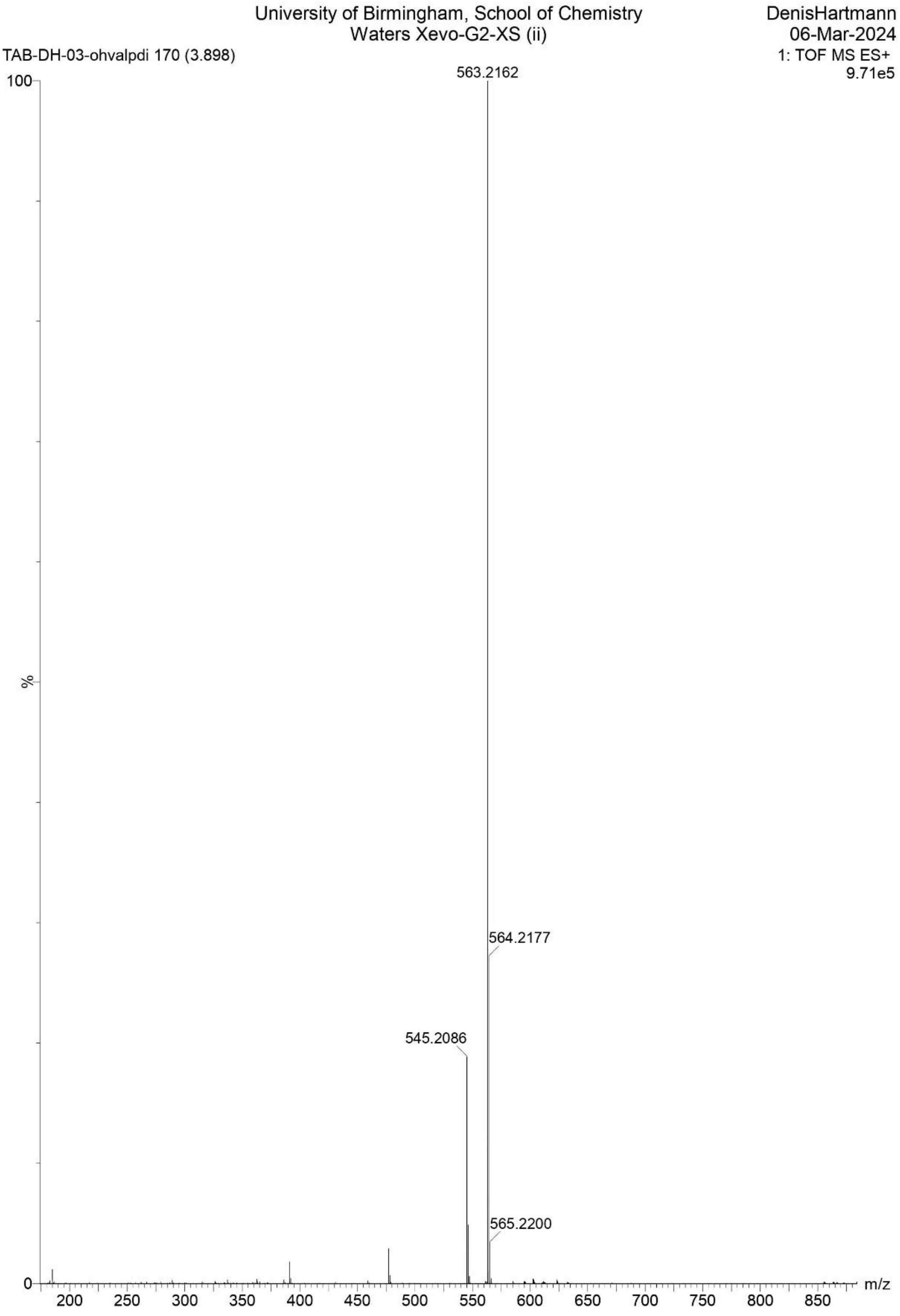

Di-TBDMS-LValinol-PDI 2

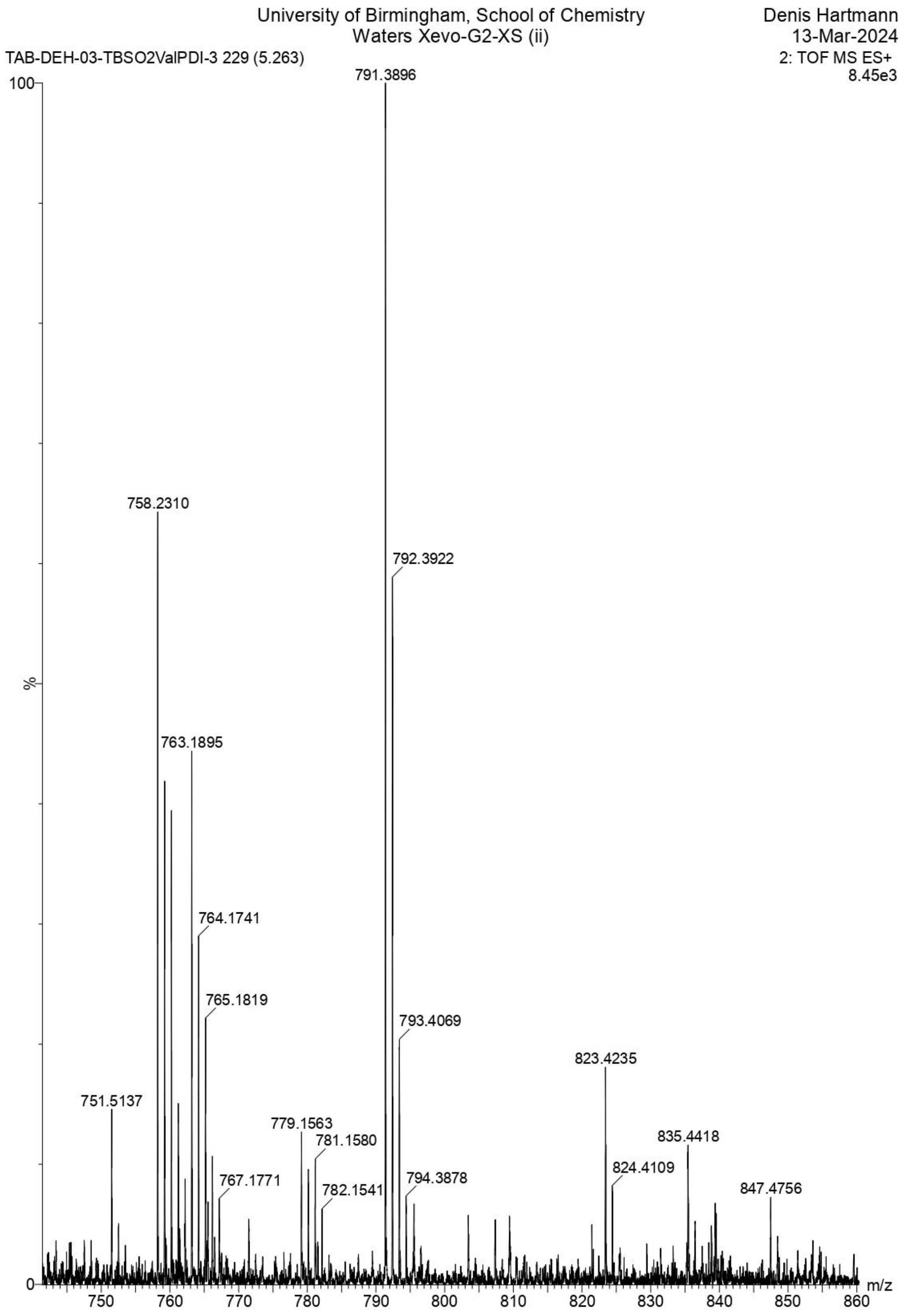

Mono-TBDMS-LValinol-PDI 3

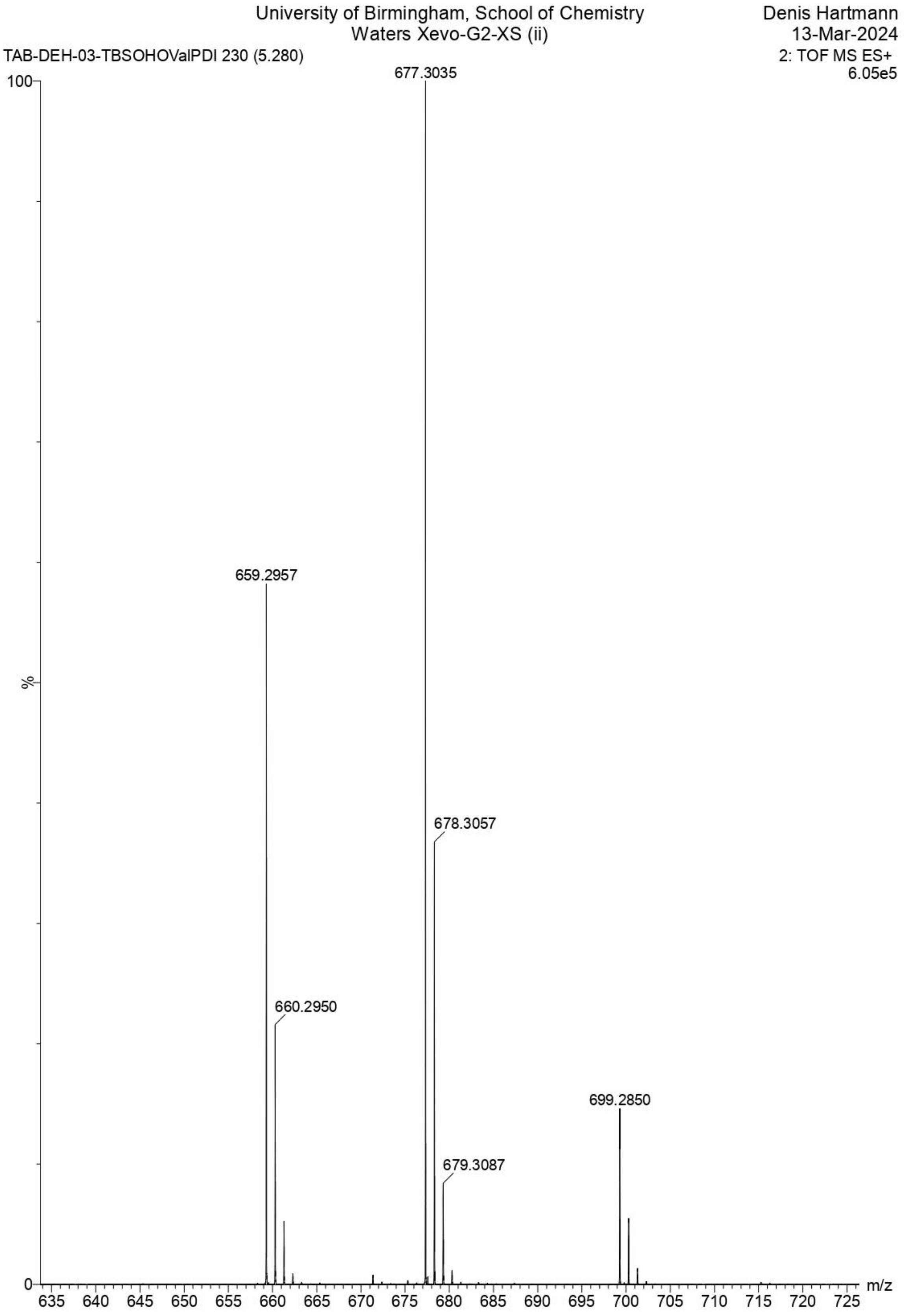

Di-TBDMS-LValinol-PDI Malonate 4

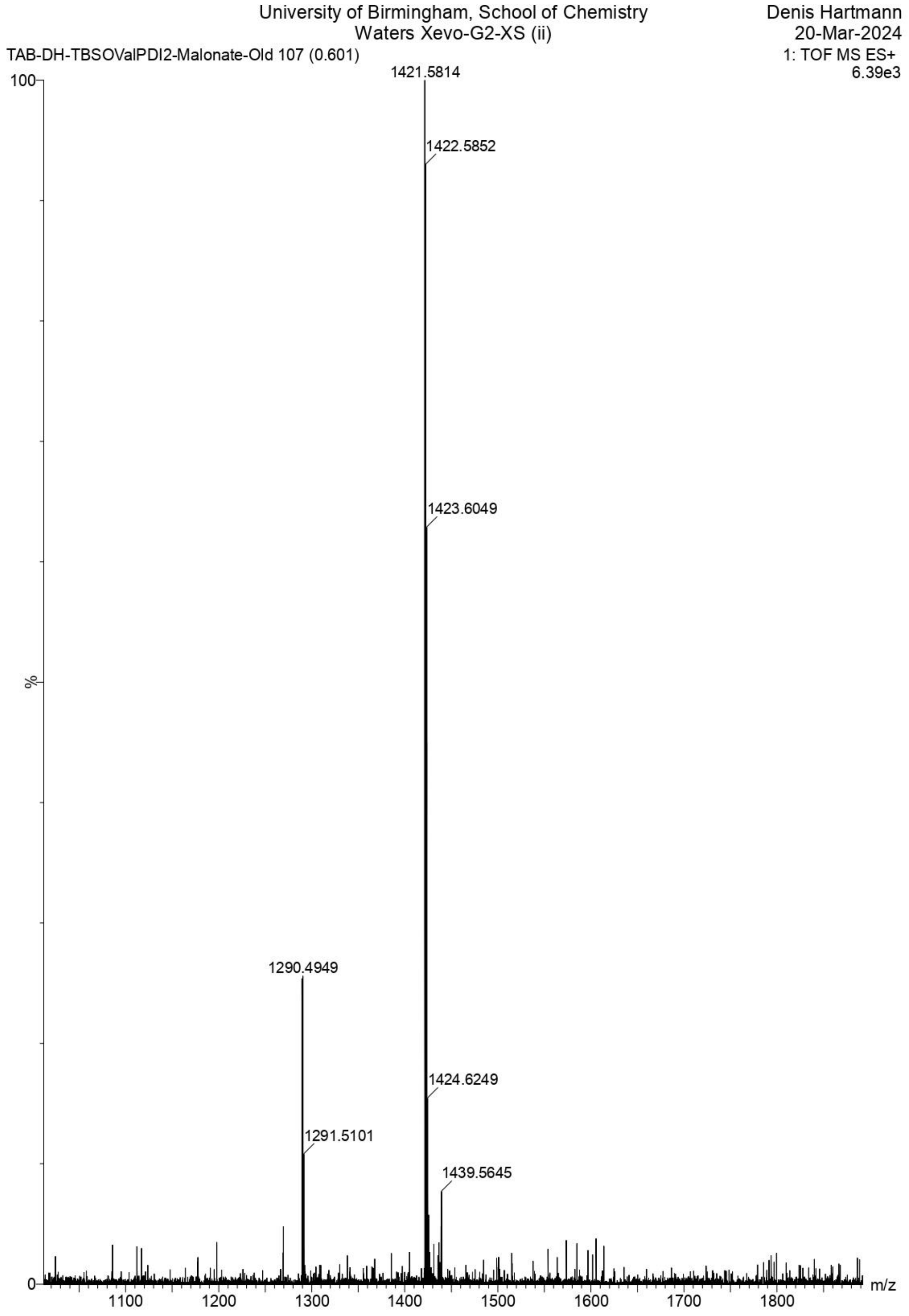

L-Valinol-Perylene-Diimide-Malonate Macrocycle 5

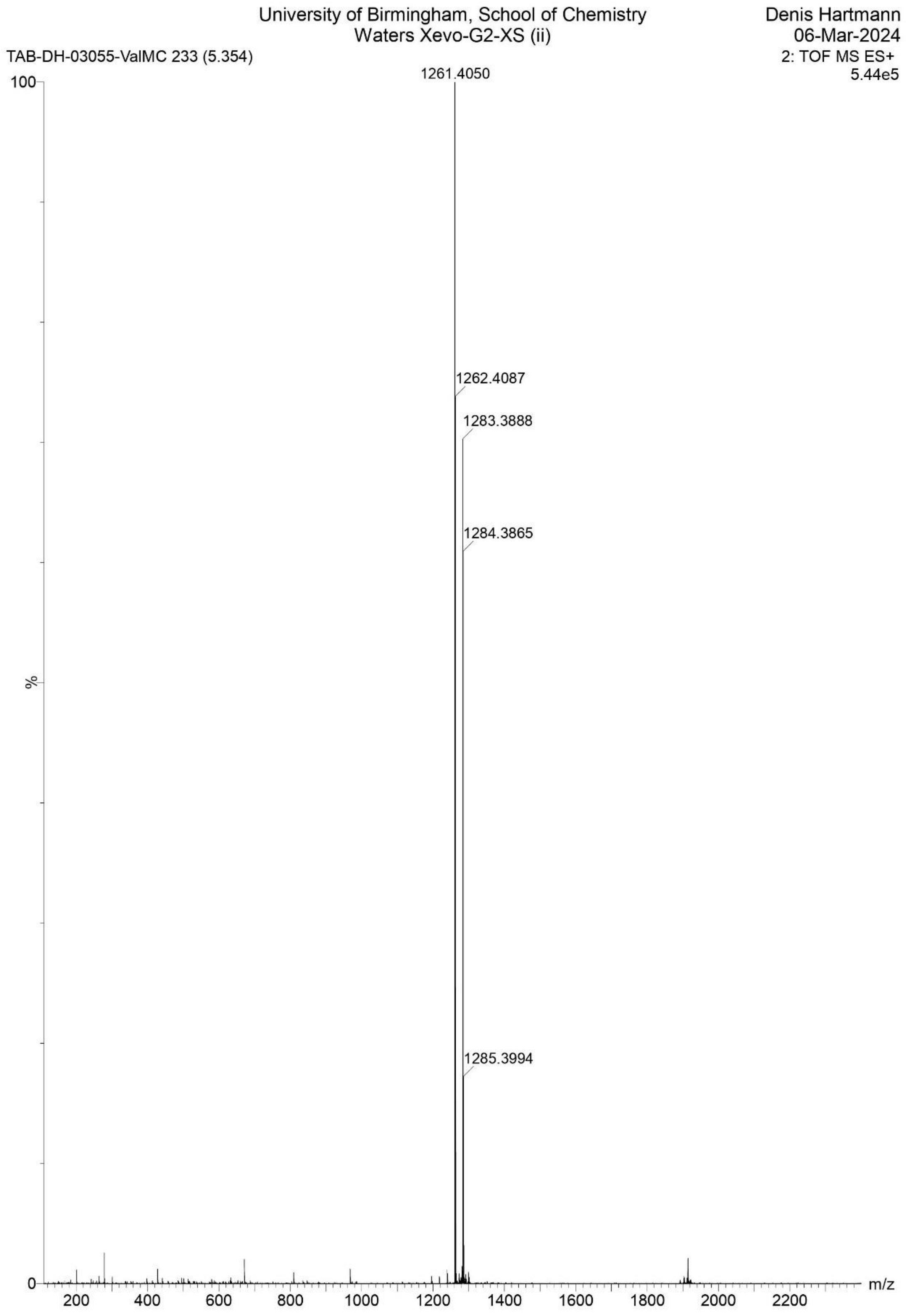

D-Valinol-Perylene Diimide (PDI) 6

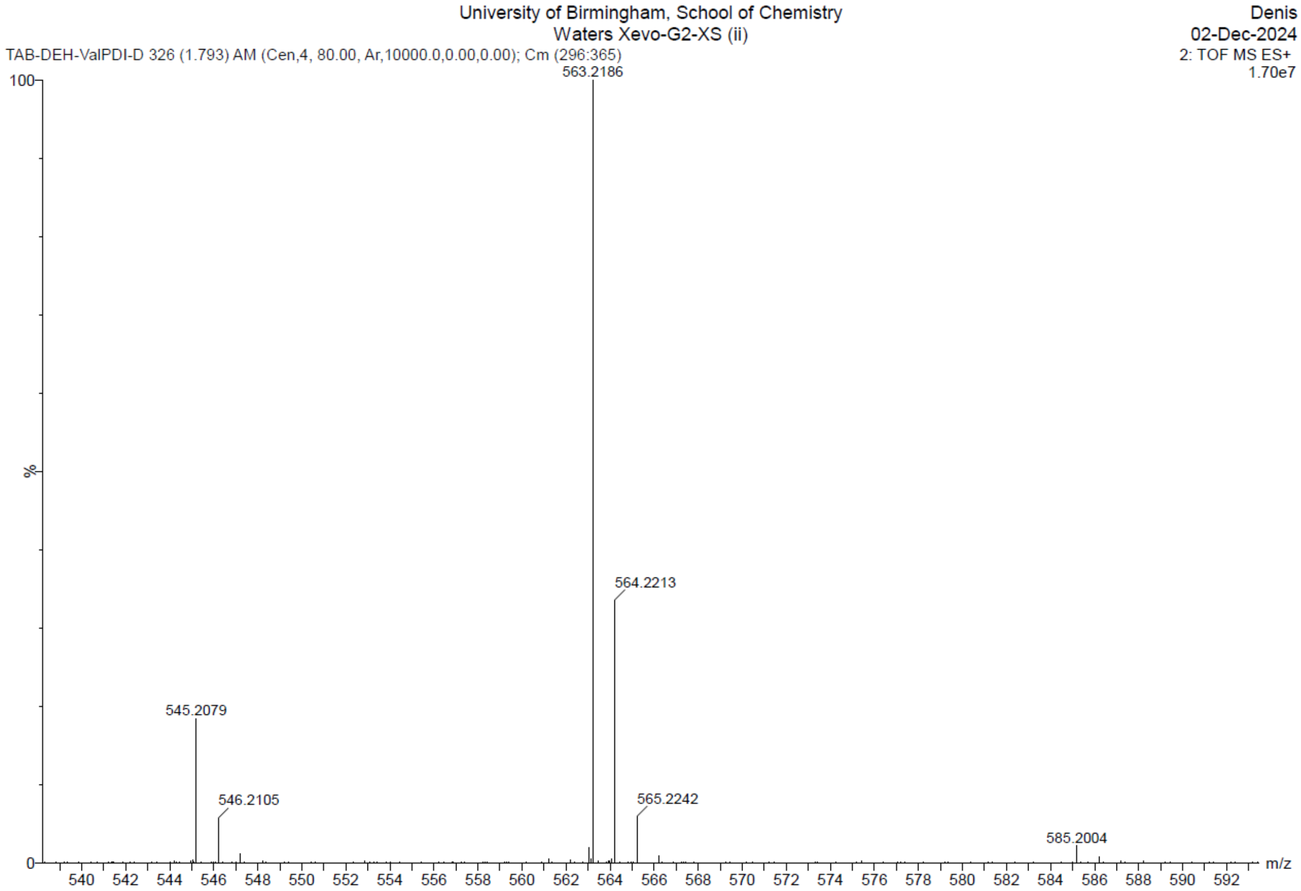

D-Valinol-Perylene-Diimide-Malonate Macrocycle 7

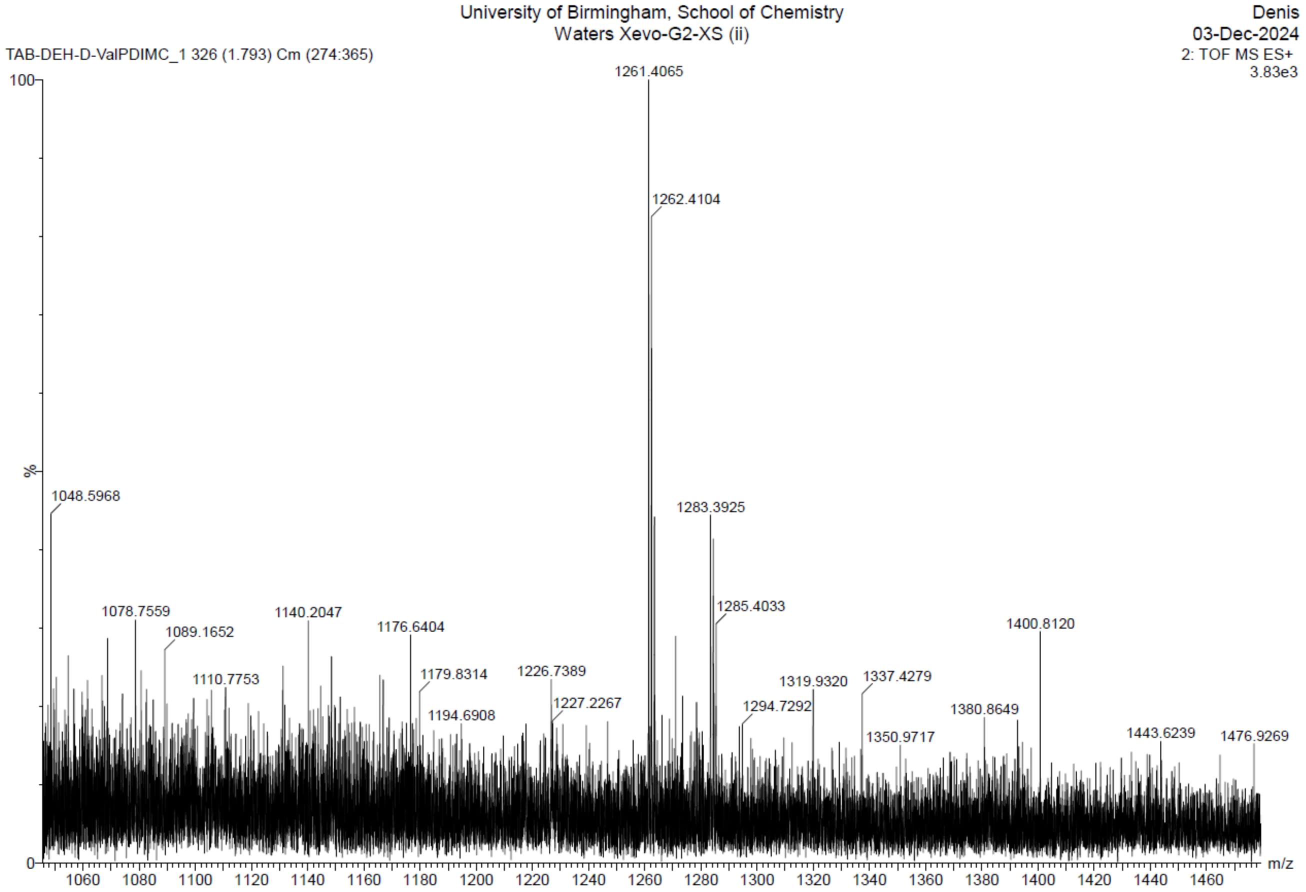

9) IR Spectrum

L-Valinol-Perylene-Diimide-Malonate Macrocycle 5

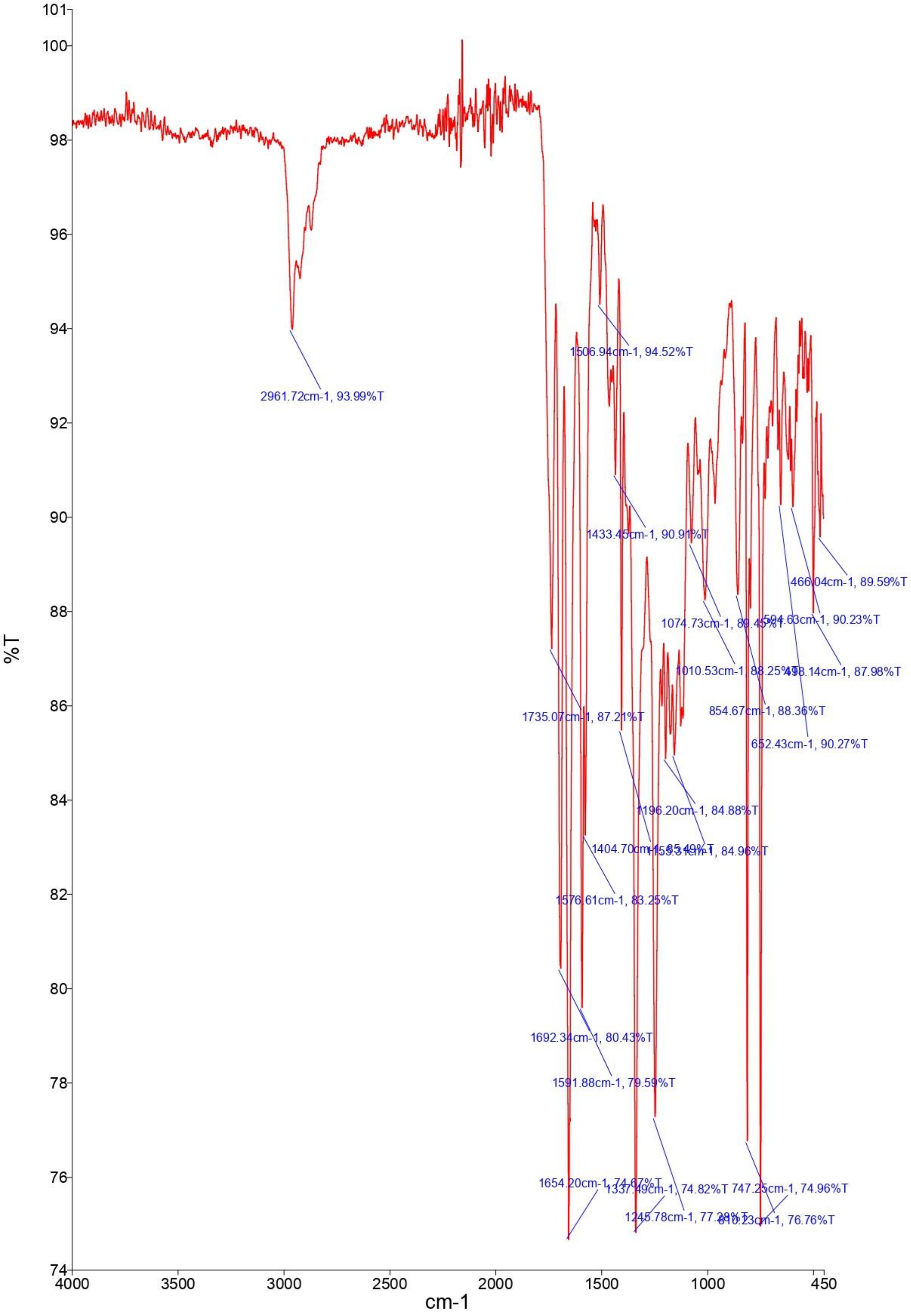

10) References

[1] M. Franceschin, N. Borbone, G. Oliviero, V. Casagrande, M. Scuotto, T. Coppola, S. Borioni, L. Mayol, G. Ortaggi, A. Bianco, J. Amato, M. Varra, *Bioconjugate Chem.* **2011**, *22*, 1309–1319.

[2] P. Spenst, R. M. Young, B. T. Phelan, M. Keller, J. Dostál, T. Brixner, M. R. Wasielewski, F. Würthner, *J. Am. Chem. Soc.* **2017**, *139*, 2014–2021.

[3] R. Carr, R. Puckrin, B. K. McMahon, R. Pal, D. Parker, L.-O. Pålsson, *Methods Appl. Fluoresc.* **2014**, *2*, 024007.

[4] G. M. Sheldrick, *Acta Cryst A* **2015**, *71*, 3–8.

[5] G. M. Sheldrick, *Acta Cryst C* **2015**, *71*, 3–8.

[6] O. V. Dolomanov, L. J. Bourhis, R. J. Gildea, J. a. K. Howard, H. Puschmann, *J Appl Cryst* **2009**, *42*, 339–341.

[7] J. G. Brandenburg, C. Bannwarth, A. Hansen, S. Grimme, *The Journal of Chemical Physics* **2018**, *148*, 064104.

[8] A. Klamt, *WIREs Computational Molecular Science* **2018**, *8*, e1338.

[9] P. Pracht, F. Bohle, S. Grimme, *Phys. Chem. Chem. Phys.* **2020**, *22*, 7169–7192.

[10] C. Bannwarth, S. Ehlert, S. Grimme, *J. Chem. Theory Comput.* **2019**, *15*, 1652–1671.

[11] S. Ehlert, M. Stahn, S. Spicher, S. Grimme, *J. Chem. Theory Comput.* **2021**, *17*, 4250–4261.

[12] J.-D. Chai, M. Head-Gordon, *The Journal of Chemical Physics* **2008**, *128*, 084106.

[13] F. Weigend, R. Ahlrichs, *Phys. Chem. Chem. Phys.* **2005**, *7*, 3297–3305.
